# Construction and validation of a novel cuproptosis-mitochondrion prognostic model related with tumor immunity in osteosarcoma

**DOI:** 10.1371/journal.pone.0288180

**Published:** 2023-07-05

**Authors:** Jinyan Feng, Jinwu Wang, Yao Xu, Feng Lu, Jin Zhang, Xiuxin Han, Chao Zhang, Guowen Wang

**Affiliations:** 1 Department of Bone and Soft Tissue Tumors, Tianjin Medical University Cancer Institute and Hospital, National Clinical Research Center for Cancer, Tianjin, China; 2 Key Laboratory of Cancer Prevention and Therapy, Tianjin, China; 3 Tianjin’s Clinical Research Center for Cancer, Tianjin, China; The First Hospital of Jilin University, CHINA

## Abstract

**Background:**

The purpose of this study was to develop a new prognostic model for osteosarcoma based on cuproptosis-mitochondrion genes.

**Materials and methods:**

The data of osteosarcoma were obtained from TARGET database. By using Cox regression and LASSO regression analysis, a novel risk score was constructed based on cuproptosis-mitochondrion genes. Kaplan-Meier, ROC curve and independent prognostic analyses were performed to validate the risk score in GSE21257 dataset. Then, a predictive nomogram was constructed and further validated by calibration plot, C-index and ROC curve. Based on the risk score, all patients were divided into high-risk and low-risk group. GO and KEGG enrichment, immune correlation and drug sensitivity analyses were performed between groups. Real-time quantitative PCR verified the expression of cuproptosis-mitochondrion prognostic model genes in osteosarcoma. And we explored the function of FDX1 in osteosarcoma by western blotting, CCK8, colony formation assay, wound healing assay and transwell assays.

**Results:**

A total of six cuproptosis-mitochondrion genes (FDX1, COX11, MFN2, TOMM20, NDUFB9 and ATP6V1E1) were identified. A novel risk score and associated prognostic nomogram were constructed with high clinical application value. Strong differences in function enrichment and tumor immune microenvironment were shown between groups. Besides, the correlation of cuproptosis-mitochondrion genes and drug sensitivity were revealed to search for potential therapeutic target. The expression of FDX1, COX11, MFN2, TOMM20 and NDUFB9 at mRNA level was elevated in osteosarcoma cells compared with normal osteoblast hFOB1.19. The mRNA expression level of ATP6V1E1 was decreased in osteosarcoma. Compared with hFOB1.19, western blotting revealed that the expression of FDX1 was significantly elevated in osteosarcoma cells. Functional experiments indicated that FDX1 mainly promoted the migration of osteosarcoma rather than proliferation.

**Conclusions:**

We developed a novel prognostic model of osteosarcoma based on cuproptosis-mitochondrion genes, which provided great guidance in survival prediction and individualized treatment decision making for patients with osteosarcoma.

## Introduction

Osteosarcoma, originating from mesenchymal tissue, is the most frequent primary malignant bone tumor and accounts for around 15% of all bone cancers [1, 2]. The annual incidence of osteosarcoma is around 4.0 to 5.0 occurrences per million, with majority of cases occurring in children and adolescents [3–5]. The lung is the most common site of recurrence and distant metastasis, and nearly 20% of patients presented lung metastasis when diagnosed with osteosarcoma [6, 7]. Although neoadjuvant chemotherapy has greatly improved the survival of osteosarcoma, patients with lung metastasis or recurrent disease presented a dismal survival outcome [8, 9]. Therefore, developing an accurate prognostic predictive model and discovering new therapeutic targets for patients with osteosarcoma are critical.

Different from apoptosis, necrosis, pyroptosis and ferroptosis, cuproptosis is a novel type of programmed cell death triggered by copper ions [10, 11]. The process of cuproptosis mainly depends on excessive accumulation of copper ions. Copper ions interacts with the lipoylated components of the tricarboxylic acid cycle (TCA cycle), which causes the aggregation of lipoylated proteins as well as the loss of iron-sulfur cluster proteins. Then, the TCA cycle is disrupted, which eventually causing protein toxic stress and cell death [10, 12]. In addition, the TCA cycle is an important part of mitochondrial respiration and TCA-related metabolites have been found to be involved in regulating cuproptosis during mitochondrial respiration [13]. Mitochondrion is the primary site of aerobic respiration and delivers energy to the cell, which is essential for cell survival. Furthermore, mitochondrion is the primary location of oxidative phosphorylation and ATP production, which contains all of the enzymes necessary for the TCA cycle. Copper ion carriers and their chelators have been discovered to be promising therapeutic molecules for tumor treatment in several studies [14, 15]. However, there was few comprehensive studies on cuproptosis-mitochondrion genes in osteosarcoma.

In the present study, we carried out a comprehensive evaluation of osteosarcoma using Therapeutics Applied Research to Generate Effective Therapies (TARGET) and Gene Expression Omnibus (GEO) database. Based on six cuproptosis-mitochondrion genes, a novel risk score was constructed and validated. Furthermore, we created a nomogram to predict the prognosis of osteosarcoma with satisfactory discrimination and calibration. In addition, a series of analyses associated with function enrichment and tumor immune microenvironment were performed to explore the possible mechanisms of cuproptosis-mitochondrion genes in osteosarcoma. We conducted a series of in vitro experiments and found that FDX1 mainly promoted the migration of osteosarcoma rather than proliferation. It suggested that inhibition of FDX1 might be a viable therapeutic option for osteosarcoma. The present study could assist to forecast the prognosis of osteosarcoma and provide new perspectives in the clinical treatment.

## Materials and methods

### Data download and preprocessing

We used TARGET database (https://ocg.cancer.gov/programs/target) to obtain mRNA expression as well as corresponding clinical data of 88 osteosarcoma patients. To further corroborate the discoveries in the TARGET construction cohort, we downloaded data from the GEO database (https://www.ncbi.nlm.nih.gov/geo/) as validation cohort (GSE21257). Patients without complete clinical data (follow-up time, age, gender, metastasis and survival status) would be excluded. Finally, a total of 84 patients with osteosarcoma from TARGET database and 53 cases from GSE21257 were included. Ethical approval was not required for this study because there was no unethical behavior and no human clinical trials or animal experiments were involved in this study.

### Identification of cuproptosis-mitochondrion genes and calculation of risk score

After literature review, a total of 33 cuproptosis-mitochondrion genes were identified, which were summarized in S1 Table [10, 16–23]. Then, we used STRING database (https://string-db.org/) to analyze the protein-protein interactions (PPIs) between these cuproptosis-mitochondrion genes and the visualization was achieved by Cytoscape software [24]. The “igraph” package was used to show expression correlation networks of these genes. In the construction cohort from TARGET database, the univariate Cox regression analysis was performed to identify specific cuproptosis-mitochondrion genes related to the prognosis of osteosarcoma. And the Kaplan-Meier method was performed to compare the survival outcome among cuproptosis-mitochondrion genes using R package "survival". Meanwhile, the least absolute shrinkage and selection operator (LASSO) regression analysis was used with R package “glmnet” for genes selection to evaluate the risk score [25]. For each sample, the cuproptosis-mitochondrion-based risk score was determined as following: Risk Score = (Coefficient gene1 × expression of gene1) + (Coefficient gene2 × expression of gene2) + ⋯ + (Coefficient genen × expression genen). The total construction cohort was divided into two cohorts based on the expression of each gene.

### The construction and validation of the risk score

Because there were so few osteosarcoma samples in public databases, we chose to divide the high- and low-risk groups using the median in order to ensure that there was enough of a sample size for comparisons between the two groups. The median value of risk score in construction cohort was used as the cut-off point to separate patients into high-risk and low-risk group. We used Kaplan-Meier method to observe the difference of survival between high- and low-risk groups. The area under the curve (AUC) was calculated using receiver operating characteristic (ROC) curve analysis to assess the prediction ability of risk score. The “stats” and “Rtsne” packages of R were used to create PCA and t-SNE based on cuproptosis-mitochondrion genes. Meanwhile, GSE21257 dataset was used to validate the predictive performance of risk score.

### Independent prognostic analysis and subgroup analysis of risk score

In order to confirm the prediction role of risk score, univariate and multivariate Cox regression analysis were performed. Patients with different clinical characteristics (age, gender, metastasis) were divided for further subgroup analysis. Kaplan Meier method was performed to examine the prognostic difference between high-risk group and low-risk group in subgroups.

### Construction and assessment of the prognostic nomogram

Most people with osteosarcoma were children and adolescents. And the prevalence of osteosarcoma was more in men than in women. Therefore, although gender and age were not correlated with osteosarcoma prognosis, adding these two factors to the nomogram could make the nomogram more comprehensive and accurate. Based on predictive factors including age, gender, metastasis and risk score, the prognostic nomogram was constructed using the R packages "rms" and "regplot" to predict the 1 -, 3 -, and 5-year overall survival rates of osteosarcoma. To evaluate the predictive performance of the nomogram, the calibration analysis and ROC curve were drawn.

### Function and pathway enrichment analysis

In order to explore the potential molecular mechanism of cuproptosis-mitochondrion genes, the enrichment items between high-risk and low-risk group were determined using GSEA and GSVA analysis [26]. The GSEA was carried out by Java GSEA v. 4.2.3, with GO terms in C5 and KEGG pathways in C2. The "GSVA" package of R was used to conduct the GSVA analysis. Genes with P<0.05 were considered significantly enriched after 1,000 permutations.

### Immune infiltration analysis and immune gene correlation

Based on CIBERSORT algorithm, the variability in 22 kinds of tumor-infiltrating immune cells (TIICs) abundance profile was calculated [27]. In addition, we used ssGSEA method to analyze changes in immune cells and immunological pathways [28]. The immune activity of 16 immune cells and 13 immunological functions were evaluated using the "gsva" package and the immune activity was favorably connected with the ssGSEA score. To explore the role of cuproptosis-related hub gene FDX1 in immune microenvironment, we divided all samples into high group and low group based on expression of FDX1 to identify different expression of TIICs [10]. Besides, the analysis of immune correlation was conducted between two groups.

### Immune checkpoint therapy

It has been found that the expression of immune checkpoint genes can predict the therapeutic effect of immune checkpoint inhibitors (ICIs) to a certain extent [29]. Thus, several ICIs (CD274, CTLA4, CD200, TNFSF14, NRP1, TNFSF4, CD244, LAG3, ICOS, CD40LG, CD48, CD200R1, HAVCR2, ADORA2A, CD276, KIR3DL1, CD80, PDCD1, LGALS9, CD160, TNFRSF14, IDO2, ICOSLG, TMIGD2, VTCN1, IDO1, PDCD1LG2, TNFSF18, BTNL2, CD70, TNFSF9, TNFRSF8, CD27, TNFRSF25, VSIR, TNFRSF4, CD40, TNFRSF18, TNFSF15, TIGIT, CD86, CD44, TNFRSF9, CD28 and CD47) were analyzed in the present study [30–33]. The expression of ICIs between high- and low-risk group was analyzed and its correlation with risk score was assessed by Spearman correlation analysis.

### Drug sensitivity analysis

In the current treatment of osteosarcoma, in addition to surgical treatment, it is necessary to combine drugs to treat osteosarcoma more effectively. Therefore, drug sensitivity is particularly important for the treatment of osteosarcoma. The CellMiner database (https://discover.nci.nih.gov/cellminer/home.do) provided drug sensitivity data. In the CellMiner database, we selected drugs with FDA approved status (218 compounds remaining after screening). To better guide therapeutic usage of clinical medicine, we investigated the association between six model genes and drug sensitivity. According to the screening condition P ≤ 0.01, sensitive drugs were ultimately obtained. The R packages “limma”, “ggplot2”, “ggpubr”, “ggExtra” and “impute” were used to examine the correlation between genes and drug sensitivity by Spearman method. To further evaluate the correlation between the risk and drug sensitivity, we conducted another drug sensitivity analysis using the R package " pRRophetic", which includes 251 drug information. To determine the half maximal inhibitory concentration (IC50) of common chemotherapeutic medicines, the R package "pRRophetic" was utilized [34]. The IC50 values between the two risk groups are compared using the Wilcoxon signed-rank test. p-value < 0.001 is used as the filtering condition.

### Cell culture

Human normal osteoblasts (hFOB1.19) were cultured in a 34°C incubator containing 5% CO_2_ using special medium (Cat#: CC0353, Shanghai Yuecun Biotechnology Co., LTD.). In an incubator at 37°C and 5% CO_2_, human osteosarcoma cells (MG63, U2OS, and 143B) were cultured with Dulbecco’s modified Eagle’s medium (DMEM; Gibco, Grand Island, NY, USA) containing 1% penicillin/streptomycin (Gibco) and 10% fetal bovine serum (FBS) in an incubator at 37°C and 5% CO2.

### Quantitative real-time fluorescence quantitative PCR (qRT-PCR)

To investigate the relative expression of genes in the prognostic model, we synthesized primers for FDX1, COX11, MFN2, TOMM20, NDUFB9 and ATP6V1E1 genes through Tianjin Zhonghe Gene Technology Co (Tianjin, China). Table 1 displayed the sequences of each primer.

**Table 1:** Primer sequences for RT-qPCR.

Genes	Forward (5‘→3’)	Reverse (5‘→3’)
GAPDH	ACAACTTTGGTATCGTGGAAGG	GCCATCACGCCACAGTTTC
ATP6V1E1	AACATAGAGAAAGGTCGGCTTG	GACTTTGAGTCTCGCTTGATTCA
COX11	TGGAGGTGCGTTCCTTTCTG	GAAACGGCTCTACCCTCTCTG
FDX1	TTCAACCTGTCACCTCATCTTTG	TGCCAGATCGAGCATGTCATT
MFN2	CTCTCGATGCAACTCTATCGTC	TCCTGTACGTGTCTTCAAGGAA
NDUFB9	CTGGGAACGAGAGGTTAAGCA	GGGTCTGGTCACAATATACCACC
TOMM20	GGTACTGCATCTACTTCGACCG	TGGTCTACGCCCTTCTCATATTC

We used TRIzol reagent (Invitrogen, Carlsbad, CA, USA) to extract total RNA from hFOB1.19, MG63, U2OS and 143B cells according to the manufacturer’s protocol. Reverse transcription of cDNA first-strand synthesis was carried out using a cDNA first-strand synthesis kit that included a step to remove gDNA (Cat#:11139ES60, Yisheng Biotechnology Co., Ltd., Shanghai, China). RT-qPCR assays were performed using a LightCycler 480 Fluorescence Quantification System (Roche, Basel, Switzerland). The relative expression levels of prognostic model genes were calculated using the 2-ΔΔCt method after normalizing all samples to GAPDH.

### Lentivirus infection

To further investigate the mechanism of FDX1 on osteosarcoma cells, we used pCDH lentiviral vector to construct FDX1 overexpression plasmid. Then, using Lipofectamine® 2000 (Invitrogen Life Technologies), pCDH-FDX1 was co-infected into 293T cells together with psPAX2 (Addgene, Inc.) and pMD2.G (Addgene, Inc.). After six hours of incubation, the 293T cell was grown in complete media (Gibco, 10% FBS) in place of DMEM. Following a further 48 hours of incubation, the supernatant was collected and concentrated using a 0.22 μm PES filter. pCDH-FDX1 packed lentiviral supernatant was used to infect U2OS cells. After infection, the medium was changed to fresh media 6 hours later, and U2OS cells were collected 48 hours later for subsequent stability screening.

### Western blotting

Total protein was extracted from osteosarcoma cells using RIPA buffer (Yeasen Biotechnology (Shanghai) Co., Ltd.) and quantified with the BCA Protein Assay Kit (Biosharp, Hefei, China). After that, protein samples were transferred onto 0.45 μm polyvinylidene fluoride (PVDF) membranes, sealed in 10% skim milk, and incubated with primary antibody (FDX1, ab108257, abcam; β-actin, #AF7018, affinity) at 4°C for an overnight period. Then incubate with secondary antibody (Beyotime Biotechnology) for 2 h at room temperature after washing with phosphate-buffered saline (PBS). Finally, the blots were visualized by enhanced chemiluminescence enhancement and analyzed by ImageJ software.

### Cell proliferation assay

We used cell counting kit‐8 assay kit (CCK-8; Yeasen Biotechnology (Shanghai) Co., Ltd.) and colony formation assay to evaluate the effect of FDX1 on the proliferation of osteosarcoma cells. For the CCK‐8 assay, Initially, U2OS and OE-FDX1-U2OS cells in the logarithmic growth phase were seeded into 96-well plates at a 2000 cells/well density and incubated. After 24, 48, and 72 hours, we added 10% CCK-8 solution and continued incubation for another 2 hours. Finally, the absorbance values at 450 nm for each well were measured using a microplate reader. For the colony formation assay, we first seeded 500 U2OS and OE-FDX1-U2OS cells in a six-well plate and incubated them for 2 weeks. Then, we fixed the samples with 4% paraformaldehyde for 15 minutes and washed them three times with phosphate-buffered saline (PBS). Next, we stained the colonies with 0.1% crystal violet for 30 minutes, captured images using a camera and calculated the number of colonies.

### Wound healing assay and transwell assay

By using the wound healing test and the transwell assay, we assessed the effect of FDX1 on the migratory capacity of osteosarcoma cells. For the wound healing assay, we cultivated U2OS and OE-FDX1-U2OS cells in 6-well plates until they achieved 95% confluence. The wound was then scraped with a 1ml pipette tip and washed with PBS to remove cellular debris and suspended cells. The scratched area was photographed at 0 and 24 hours, and the percentage of wound healing was calculated using the ratio of the healed area to the initial wound area. For the transwell assay, 4×10^5 U2OS or OE-FDX1-U2OS cells were added to the upper chamber of the transwell plate and the lower chamber was filled with 500ul of medium supplemented with 10% FBS. After 24 h of incubation, the migrated cells were fixed, stained with crystal violet (0.1%) and observed under a microscope at ×20 magnification.

### Statistical analysis

The statistical analysis was carried out using the R software (version 3.6.2). The T-test and independent-sample T test were performed to analyze the differences. The result was considered statistical significance when p-value was less than 0.05.

## Results

### Construction of the risk score for osteosarcoma based on cuproptosis-mitochondrion genes

**Fig 1:**
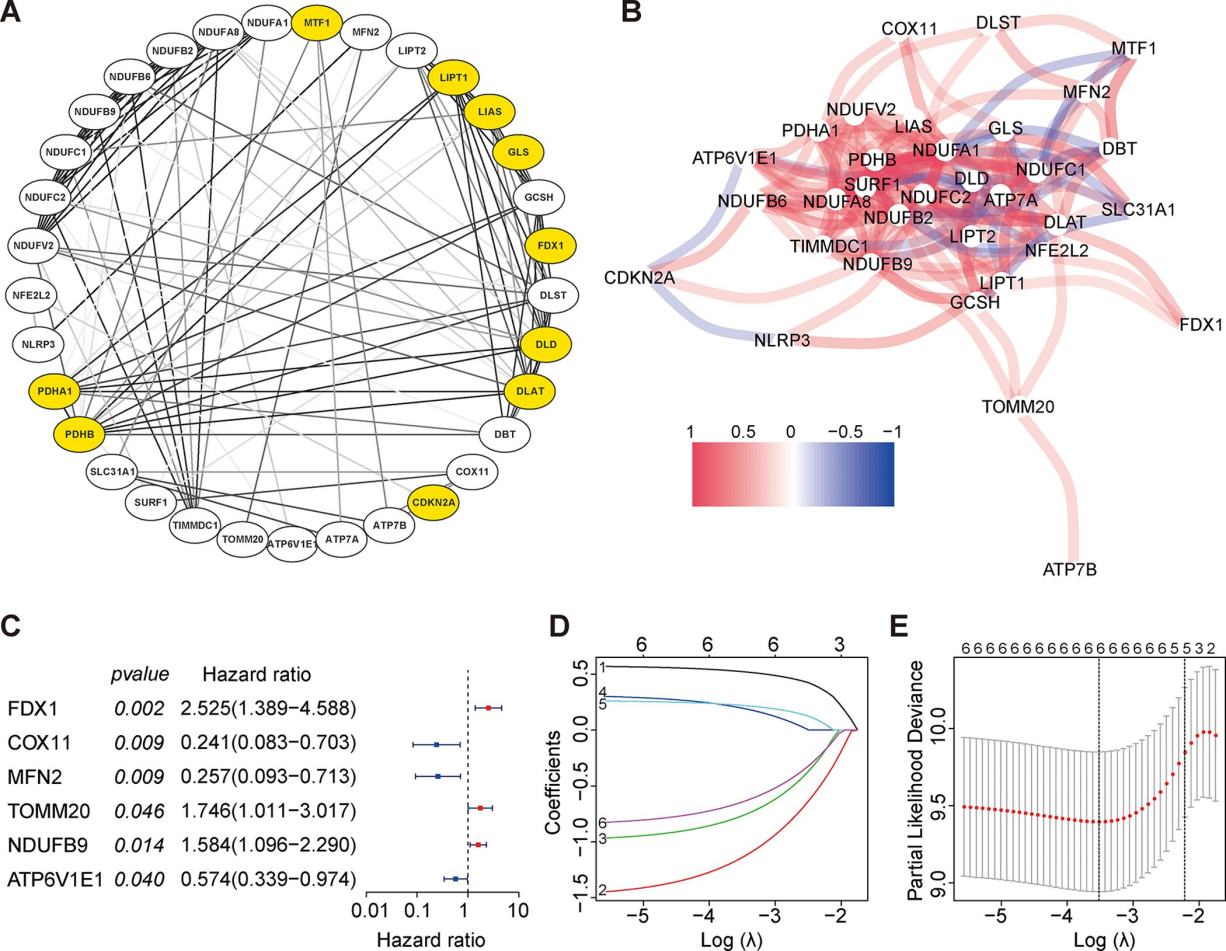
The selection of cuproptosis-mitochondrion genes for further construction of risk score. (A) Protein-protein interaction (PPI) network of the cuproptosis-mitochondrion genes. (B) Correlation network of cuproptosis-mitochondrion genes. (C) Six cuproptosis-mitochondrion genes were associated with prognosis of osteosarcoma in univariate Cox regression analysis. (D and E) After LASSO regression analysis, all of the six cuproptosis-mitochondrion genes were selected to construct risk score.

Cuproptosis is a novel type of cell death triggered by increased accumulation of copper ions in the intracellular compartments, which mainly depends on FDX1-mediated mitochondrial proteotoxic stress [11]. Thus, to clarify the connection among cuproptosis-mitochondrion genes, we created a PPI network using STRING database (Fig 1A). The number of nodes was displayed in S1 Fig. As shown in Fig 1B, these cuprotosis-mitochondrion genes were all linked by varying degrees of interaction. Then, based on the construction cohort, the univariate Cox regression analysis was adopted. A total of six cuproptosis-mitochondrion genes (FDX1, COX11, MFN2, TOMM20, NDUFB9 and ATP6V1E1) were identified to be associated with the prognosis of osteosarcoma (Fig 1C). The survival curves of six cuproptosis-mitochondrion genes were shown based on their expression (S2 Fig). Patients with high FDX1 expression presented significant lower survival rate than those with low expression (S2A Fig). Meanwhile, the high expression of COX11 (S2B Fig), MFN2 (S2C Fig) and ATP6V1E1 (S2F Fig) were associated with better survival outcome. The six genes were further selected using the LASSO regression analysis and all of these genes were included into the final calculation of the risk score (Fig 1D and 1E). The risk score was calculated as followed: Risk score = (0.524041688634354 × expression of FDX1) + (-1.1457013255643 × expression of COX11) +(-0.770737892454771 × expression of MFN2) + (0.198383018312821 × expression of TOMM20) + (0.221880303088796 × expression of NDUFB9) + (-0.6471909471031 × expression of ATP6V1E1). At last, the risk score based on the expression of six cuproptosis-mitochondrion genes was constructed and used for further analyses. The corresponding clinical data of 84 osteosarcoma cases from the TARGET database and 53 osteosarcoma patients from the GEO database are shown in Table 2.

**Table 2:** The osteosarcoma patient data in training and validation sets.

Item	Training cohort (TARGET, n = 84)	Validation cohort (GSE21257, n = 53)
Age, n (%)		
≤16	51 (60.7)	25 (47.2)
> 16	33 (39.3)	28 (52.8)
Gender, n (%)		
Male	47 (56.0)	34 (64.2)
Female	37 (44.0)	19 (35.8)
Metastasis, n (%)		
Nonmetastatic	63 (75.0)	19 (35.8)
Metastasis	21 (25.0)	34 (64.2)
Status, n (%)		
Alive	57 (67.9)	30 (62.3)
Dead	27 (32.1)	23 (37.7)

### The construction and validation of the risk score and independent prognostic analysis

**Fig 2:**
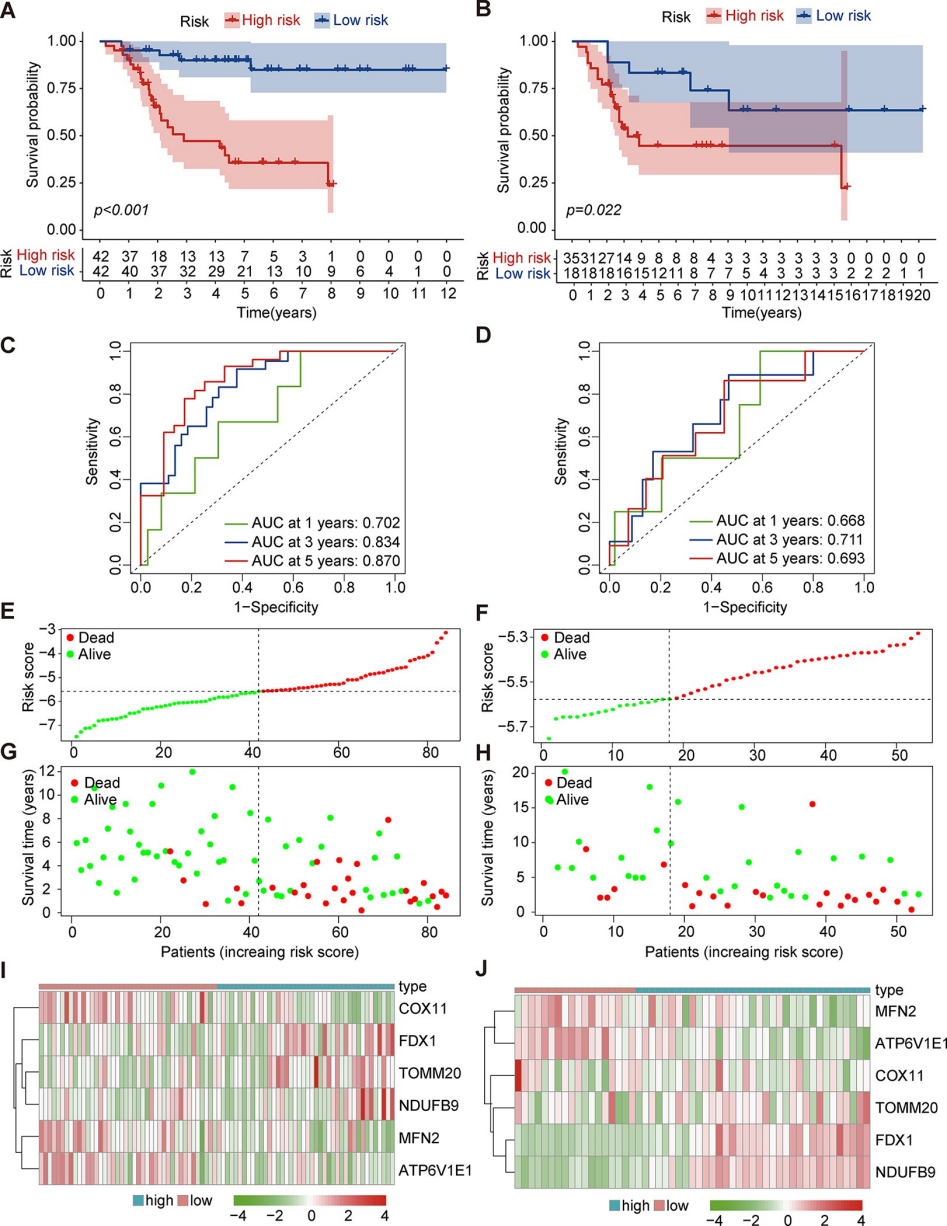
The construction and validation of the prognostic risk score based on six cuproptosis-mitochondrion genes (A, C, E, G and I for construction cohort from TARGET database; B, D, F, H and J for validation cohort from GSE21257). The Kaplan-Meier curve of patients in high-risk and low-risk group (A and B). The ROC curve of the 1-, 3- and 5- year overall survival (C and D). The distribution of risk score for each osteosarcoma patient (E and F). The overall survival rate and its survival status of osteosarcoma patients (G and H). The expression of these six cuproptosis-mitochondrion genes in the low-risk group and the high-risk group. Cool color represents low expression while warm color represents high expression (I and J).

According to the median value of risk score in the construction cohort, all of patients were divided into two groups, namely high-risk group and low-risk group. The Kaplan-Meier procedure was adopted to assess the predictive value of the risk score. In the construction cohort, high-risk group was associated with poor overall survival outcome (Fig 2A), which was verified in validation cohort from GSE21257 (Fig 2B). Then, the ROC curve was plotted and the AUC of 1-year, 3-year and 5-year overall survival were 0.702, 0.834 and 0.870, respectively in construction cohort (Fig 2C). The risk score and survival status of each sample were displayed in risk curve and scatter plot (Fig 2E–2G). In addition, the heatmap showed the different expression of six cuproptosis-mitochondrion genes in high-risk group and low-risk group (Fig 2I). Besides, all of samples were distinguished into two main components by PCA and t-SNE analysis (S3A and S3C Fig). When validating these results in validation cohort, the similar findings were achieved (Fig 2, S3B and S3D Fig). These above results confirmed that the risk score could predict survival of osteosarcoma accurately.

**Fig 3:**
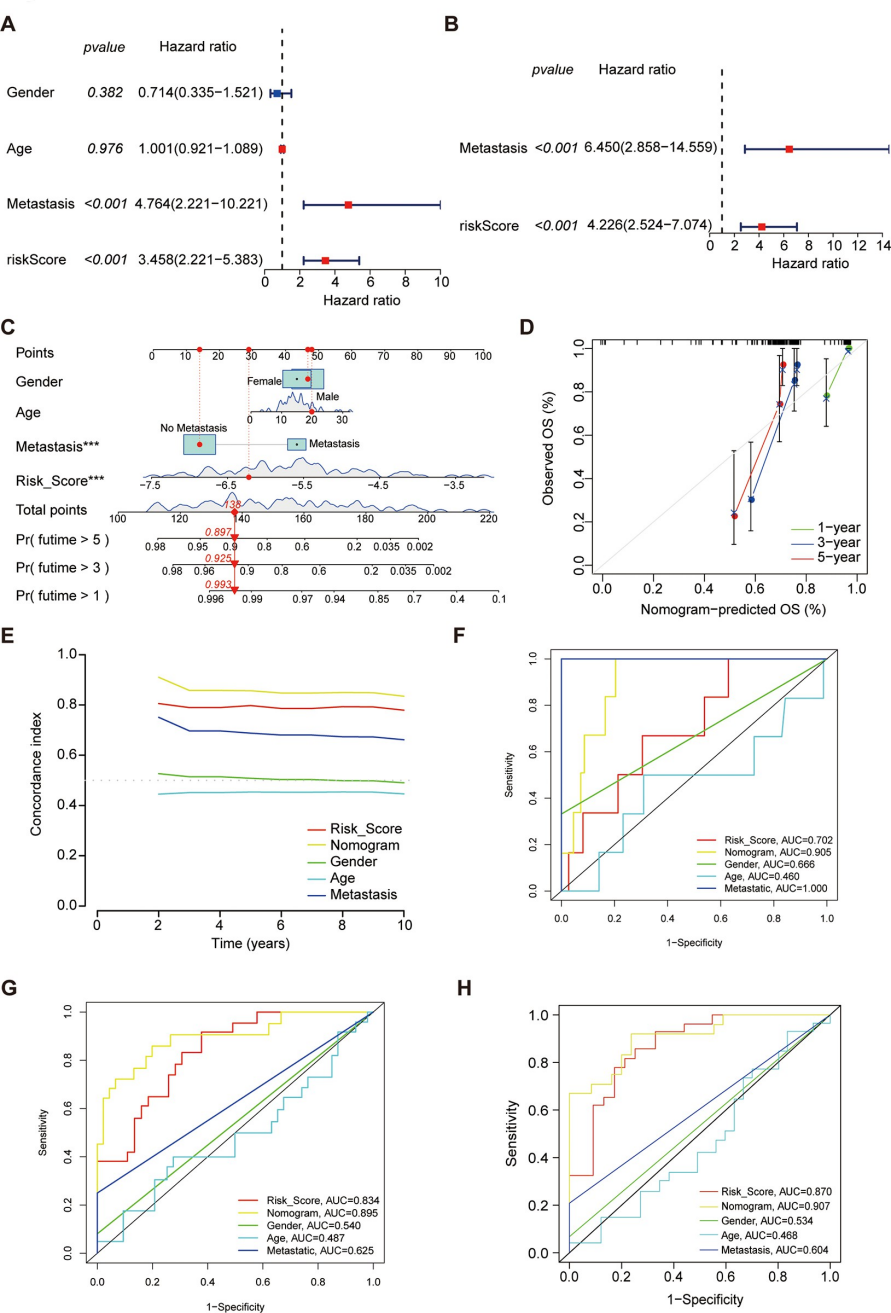
Independent prognostic analysis and the nomogram predicting the survival outcome of osteosarcoma. The univariate (A) and multivariate Cox (B) regression analyses were performed. (C) The nomogram was constructed based on gender, age, metastasis and risk score. (D) The calibration curve for predicting 1-,3‐, and 5-year overall survival. (E) The C-index of predictive factors in the nomogram according to times. The 1-year (F), 3-year (G), and 5-year (H) ROC curves of predictive factors are used to calculate AUC values.

We performed Cox regression analysis to investigate the link of prognosis between risk score and other clinical characteristics (age, gender and metastasis). The results revealed that risk score and metastasis were two independent prognostic factors for osteosarcoma (Fig 3A and 3B). Furthermore, the predictive performance of risk score was clarified in subgroup analysis (S4 Fig). Overall, the risk score showed satisfactory performance of prediction in clinical subgroup analysis.

### Development and evaluation of the prognostic nomogram

After integration risk score and three clinicopathological features (gender, age, metastasis), we created a prognostic nomogram to predict the 1-, 3-, and 5-year survival probabilities of osteosarcoma (Fig 3C). The calibration chart demonstrated excellent calibration of the nomogram (Fig 3D). When comparing C-index in several combination of predictive factors, the concrete value of both risk score and nomogram was more than 0.7, indicating favorable performance of risk score and the nomogram in the present study (Fig 3E). The nomogram’s five-year AUC was up to 0.907, which confirmed that the nomogram was highly effective in predicting survival of osteosarcoma (Fig 3F). Overall, our findings attested good accuracy and practicality of the predictive nomogram.

### Function and pathway enrichment analysis

**Fig 4:**
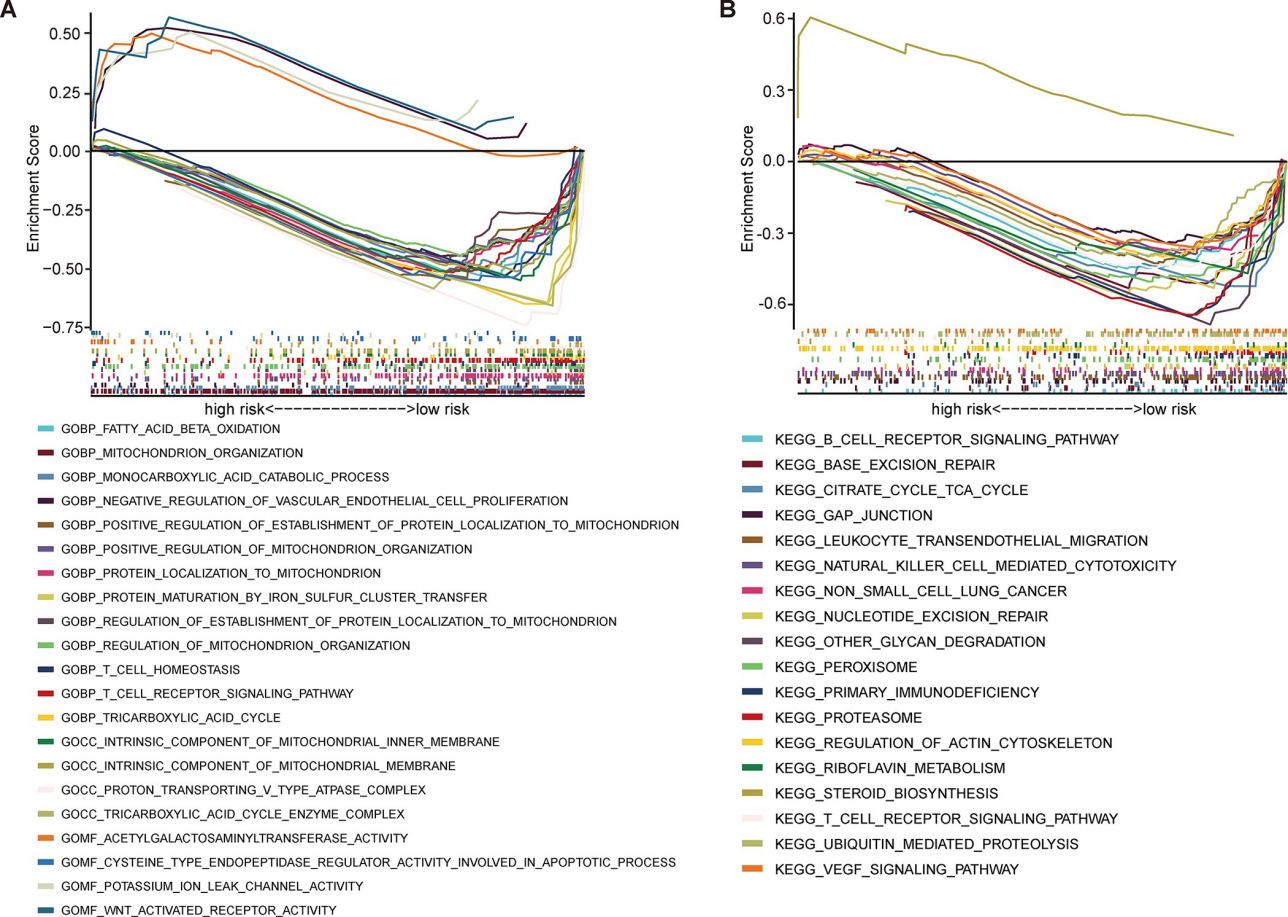
Gene set enrichment analysis between the low-risk group and high-risk group. (A) Enriched GO terms between high-risk group and low-risk group. (B) Enriched KEGG pathways between high-risk and low-risk group.

To deeply specify the potential molecular mechanism and biology pathways of the six cuproptosis-mitochondrion genes, the GO and KEGG enrichment analysis were performed. In the GO enrichment analysis, the gene set was shown to be enriched in tricarboxylic acid cycle, tricarboxylic acid cycle enzyme complex, intrinsic component of mitochondrial membrane, T cell receptor signaling pathway, protein maturation by iron-sulfur cluster transfer, positive regulation of establishment of protein localization to mitochondrion (Fig 4A). Meanwhile, the six genes were enriched in citrate cycle TCA cycle, T cell receptor signaling pathway, nucleotide excision repair, peroxisome, regulation of actin cytoskeleton and VEGF signaling pathway according to KEGG enrichment analysis. (Fig 4B). In addition, we further studied key pathways in different risk groups using GSVA. As shown in S5 Fig, similar KEGG enrichment results of GSEA were obtained. These results indicated that our cuproptosis-mitochondrion genes might be involved in regulation of tumor immunity.

### Immune landscape and immune checkpoint inhibitor

**Fig 5:**
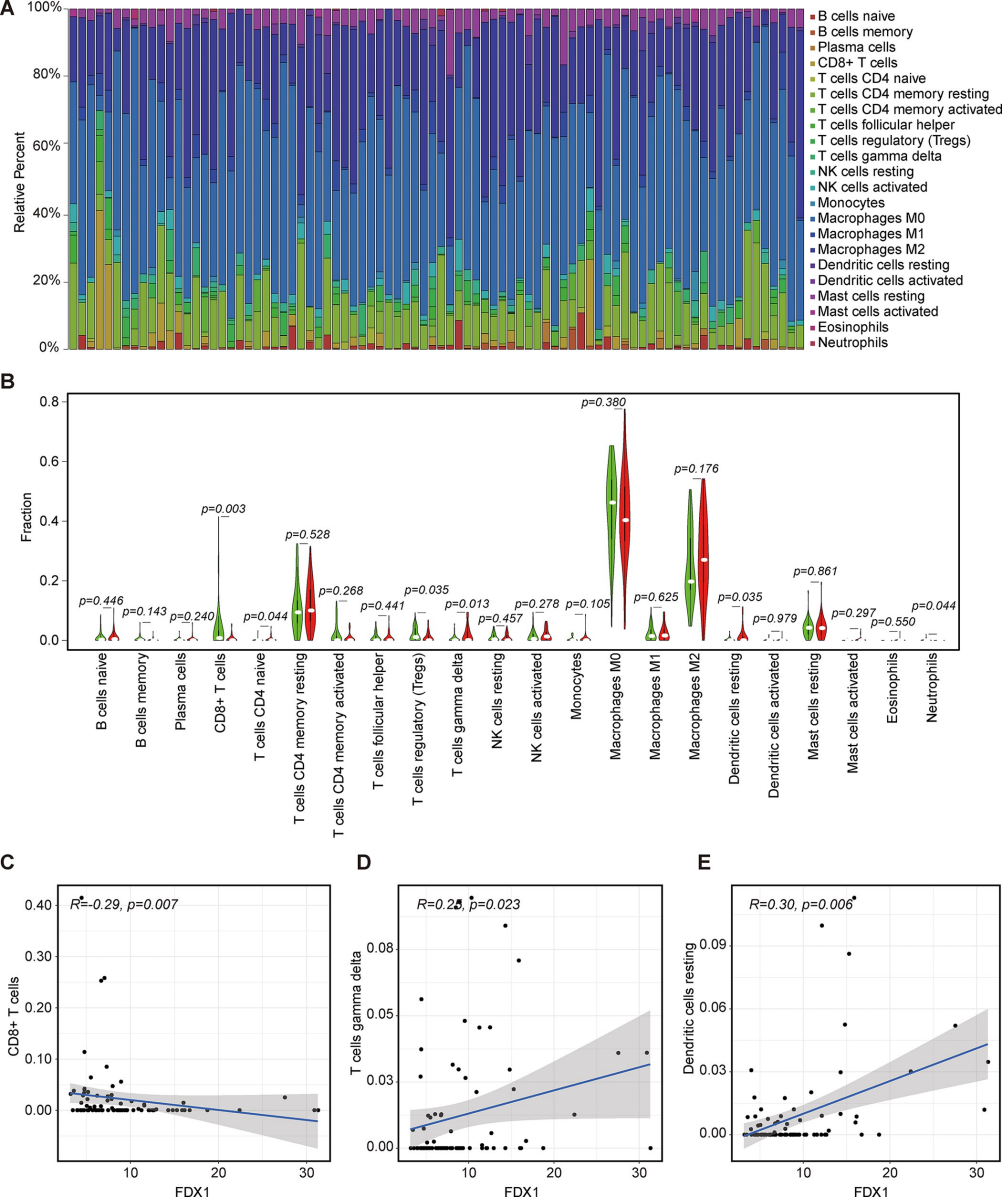
Immune correlation analysis. (A) The bar plot of 22 immune cell infiltrations in CIBERSORT. (B) Violin plot showed the ratio differentiation of 22 kinds of immune cells between osteosarcoma samples with low or high expression of FDX1, and Wilcoxon rank sum was used for the significance test. (C-E) T cells gamma delta and Dendritic cells resting were positively connected with the expression of FDX1 while CD8+ T cells was negatively correlated with the expression.

The infiltration of immune cells in tumor microenvironment was associated with the response to immunotherapy, and it was reported that cell death can trigger cancer-related immune response [35, 36]. Using the CIBERSORT algorithm, we investigated the proportion of immune cells among osteosarcoma samples. As shown in Fig 5A, the heterogeneity of osteosarcoma is obviously reflected in infiltration ratio of various immune cell. The relationship of 22 kinds of immune cells was illustrated in S6 Fig. Furthermore, the results of ssGSEA method revealed significant differences in immune cells (CD8+ T cells, DCs, Tfh and Th1 cells) between high-risk and low-risk group (S7A Fig). As for immunological function, there were significant differences between groups in cytolytic activity, inflammation promotion and T cell co-stimulation (S7B Fig). The difference analysis revealed that six types of TIICs (CD8+ T cells, T cell CD4 naïve, Tregs, T cells gamma delta, Dendritic cells resting and neutrophils) present different expression between FDX1 high-expression group and FDX1 low-expression group (Fig 5B). After immune correlation analysis, three TIICs were associated with expression of FDX1. Among them, T cells gamma delta and Dendritic cells resting were positively connected with the expression of FDX1, whereas CD8+ T cells was found to be negatively correlated with the expression. These results indicated that FDX1 might affect immune activity in tumor immune environment (Fig 5C–5E).

**Fig 6:**
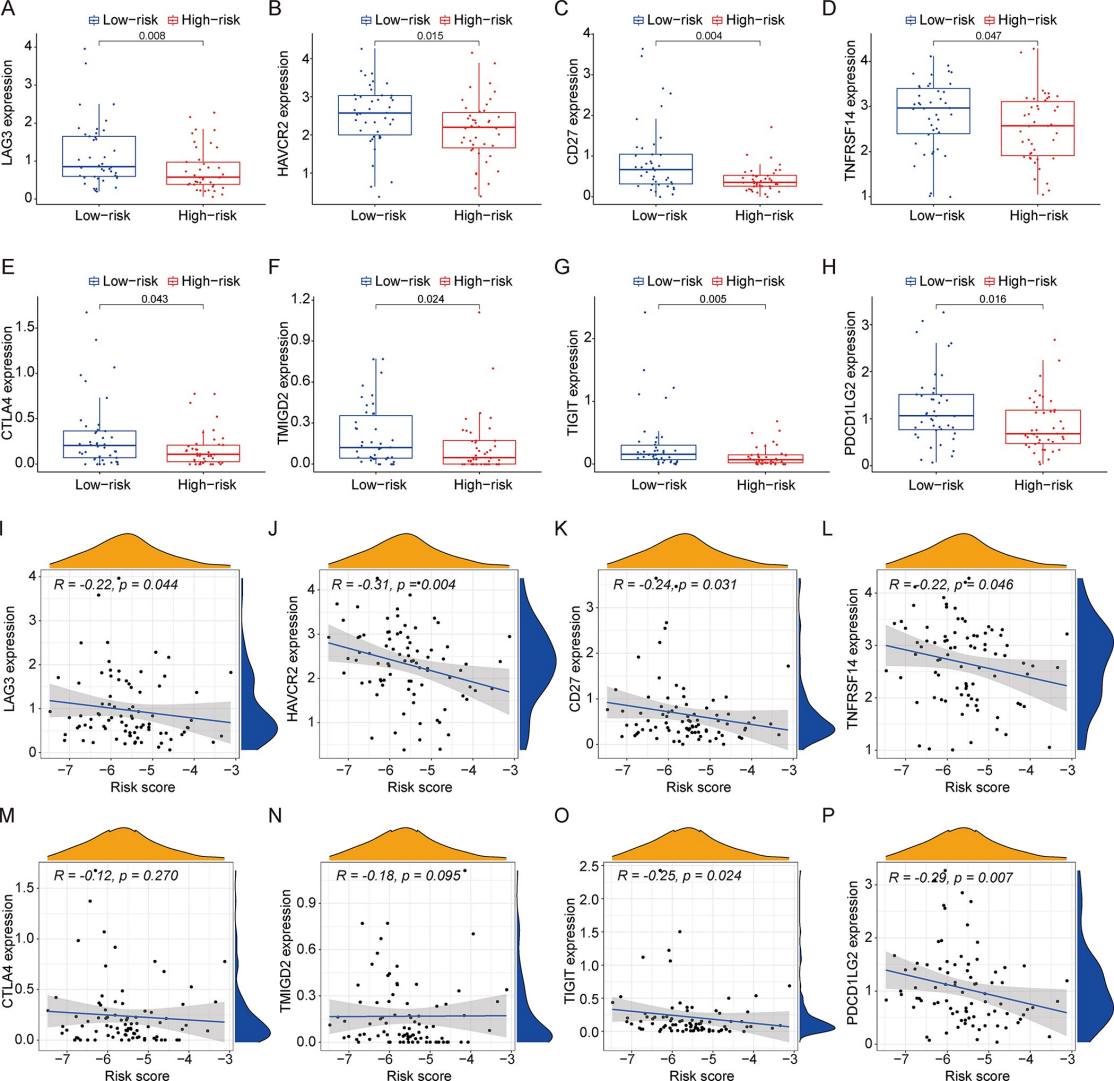
The expression of immune checkpoint genes between low-risk and high-risk group and its correlation with risk score. (A-H) The comparison of the expression levels of LAG3, HAVCR2, CD27, TNFRSF14, CTLA4, TMIGD2, TIGIT and PDCD1LG2 between high-risk and low-risk group. (I-P) Significant association between immune checkpoint genes and risk score were identified.

Immune checkpoint inhibitors played an important role in treatment of osteosarcoma, so we discussed the different expression of immune checkpoint genes between low-risk group and high-risk group. Our results showed that the expressions of LAG3, HAVCR2, CD27, TNFRSF14, CTLA4, TMIGD2, TIGIT and PDCD1LG2 in low-risk osteosarcoma group were higher than those in high-risk group (Fig 6A–6H). Meanwhile, the expression of LAG3 (P = 0.044, Fig 6I), HAVCR2 (P = 0.004, Fig 6J), CD27 (P = 0.031, Fig 6K), TNFRSF14 (P = 0.046, Fig 6L), TIGIT (P = 0.024, Fig 6O) and PDCD1LG2 (P = 0.007, Fig 6P) was negatively with risk score by spearman correlation analysis. Therefore, osteosarcoma patients in low-risk group might be benefited from targeted-therapy of immune checkpoint genes.

### The relationship between cuproptosis-mitochondrion genes and drug sensitivity

**Fig 7:**
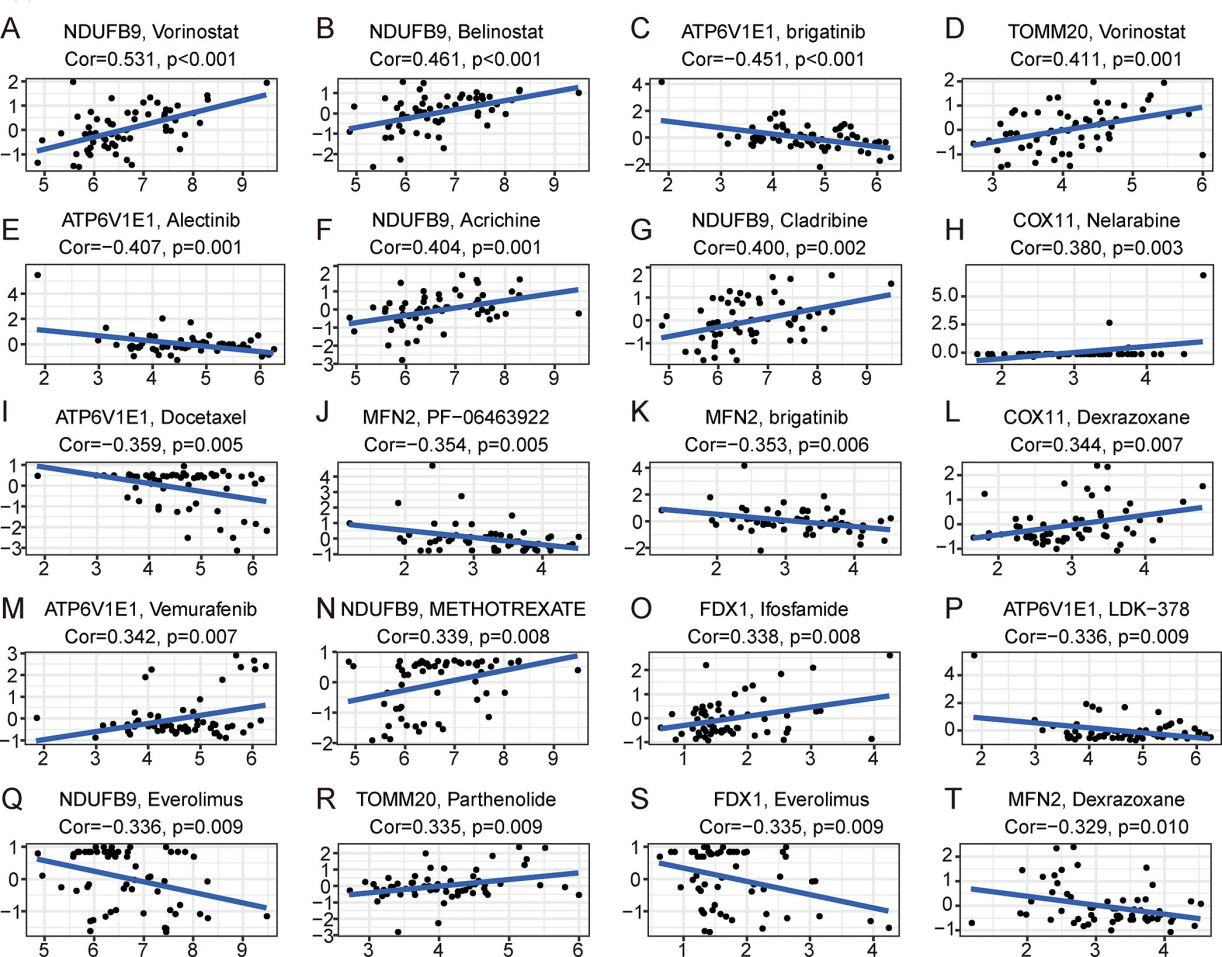
The correlation between six cuproptosis-mitochondrion genes and sensitivity of drug. Gene expression is depicted horizontally while drug sensitivity is depicted vertically. Correlation coefficient R>0 was considered as a positive correlation, and *P<0*.*05* was considered as a significant difference.

In order to explore the potential targets, we evaluated the correlation between cuproptosis-mitochondrion genes and drug sensitivity. FDX1 was shown to be positively correlated with drug sensitivity of ifosfamide (Fig 7O) but negatively correlated with everolimus (Fig 7S). Then, TOMM20 was identified to enhance the sensitivity to vorinostat (Fig 7D) and parthenolide (Fig 7R) while COX11 enhanced the sensitivity to nelarabine (Fig 7H) and dexrazoxane (Fig 7L). Besides, NDUFB9 was found to be positively associated with the drug sensitivity of several anticancer drugs including vorinostat (Fig 7A), belinostat (Fig 7B), acrichine (Fig 7F), cladrbine (Fig 7G) and methotrexate (Fig 7N) while negatively associated with everolimus (Fig 7Q). Moreover, MFN2 weakened the sensitivity of PF-06463922 (Fig 7J), brigatinib (Fig 7K) and dexrazoxane (Fig 7T). The expression of ATP6V1E1 was negatively correlated with brigatinib (Fig 7C), alectinib (Fig 7E), docetaxel (Fig 7I) and LDK-378 (Fig 7P), indicating that with the increase of the expression level of ATP6V1E1, the sensitivity to drug decreased. Meanwhile, the expression of ATP6V1E1 was positively associated with vemurafenib (Fig 7M), indicating that the sensitivity of vemurafenib increased along with increased expression of ATP6V1E1. Furthermore, we assessed the chemotherapy sensitivity between two groups of patients with high and low risk scores. We compared the IC50 levels of 251 chemotherapy drugs between the high and low risk groups. Based on screening criteria (P<0.001), we identified eight sensitive drugs (Cyclopamine, GNF-2, CGP-082996, I-BET-762, KIN001-244, THZ-2-102-1, PF-4708671 and PFI-1) (S8 Fig). Notably, we observed that the IC50 values of all eight chemotherapy drugs were lower in osteosarcoma patients with high-risk scores than those with low-risk scores, indicating that osteosarcoma patients with high-risk scores may exhibit greater sensitivity to these eight chemotherapy drugs (S8 Fig). In conclusion, the correlation between cuproptosis-mitochondrion genes and drug sensitivity might instruct us in making individualized treatment decision for patients with osteosarcoma.

### The expression of cuproptosis-mitochondrion prognostic genes in osteosarcoma

**Fig 8:**
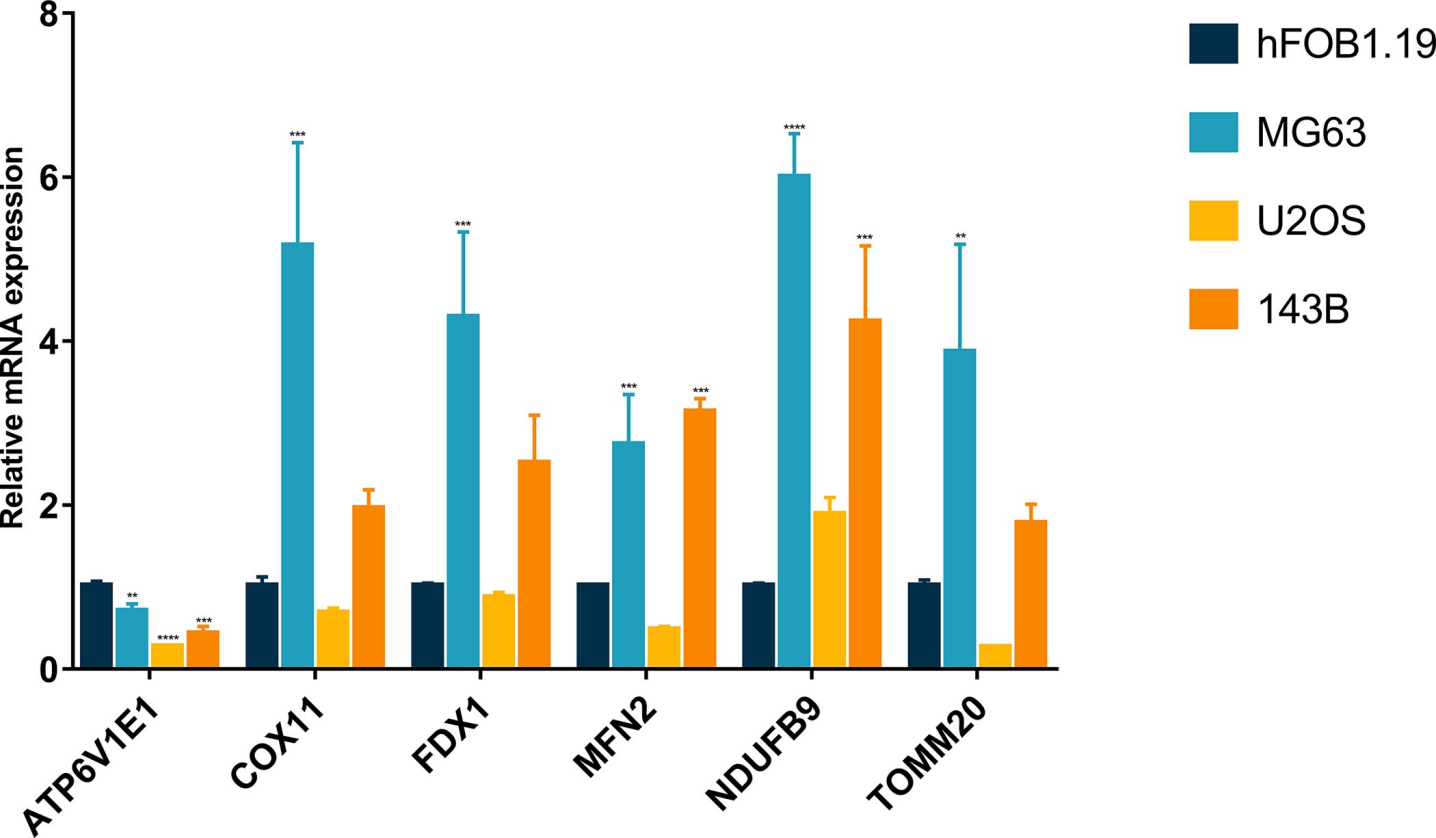
The mRNA expression level of cuproptosis-mitochondrion prognostic model genes. *p < 0.05, **p < 0.01, ***p < 0.001, ****p < 0.0001 each experiment was repeated three times.

To further evaluate the expression of cuproptosis-mitochondrion prognostic genes in osteosarcoma, three osteosarcoma cells (MG63, U2OS and 143B) were selected to detect their mRNA expression levels, and hFOB1.19 was selected as the control group. Compared with hFOB1.19, the mRNA expression of ATP6V1E1 was significantly down-regulated in MG63, U2OS and 143B osteosarcoma cells (Fig 8). Moreover, in comparison to hFOB1.19, the mRNA expression levels of COX11, FDX1, MFN2, NDUFB9 and TOMM20 were up-regulated in MG63 and 143B cells, and the up-regulation was most significant in MG63 cells (Fig 8). In U2OS cells, no statistical significance was found of the mRNA expression level of COX11, FDX1, MFN2, NDUFB9 and TOMM20 (Fig 8).

### FDX1 mainly leads to malignancy of osteosarcoma through migration, rather than proliferation

**Fig 9:**
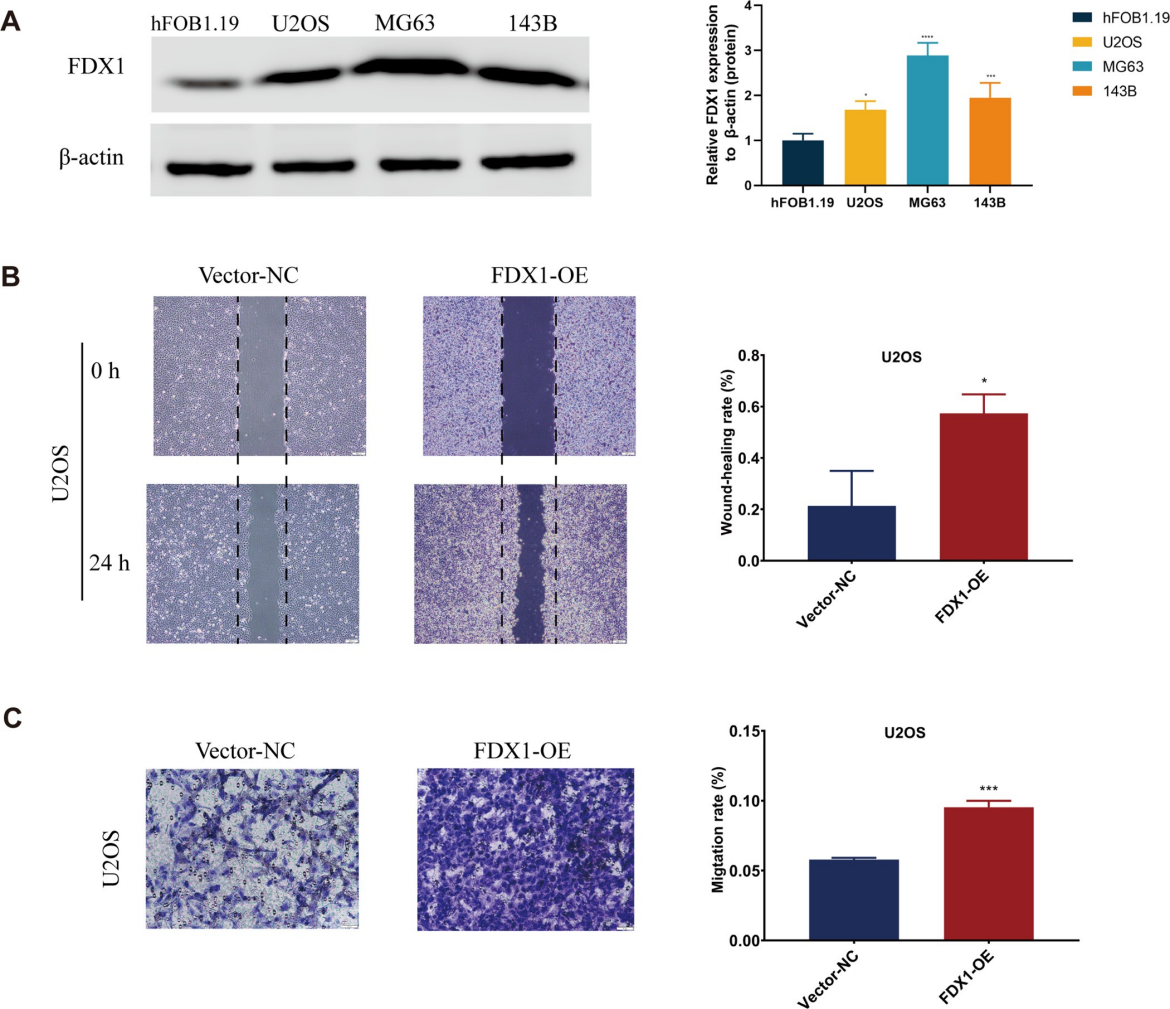
The expression and migration of FDX1 in osteosarcoma. (A)The protein expression levels of FDX1 in hFOB1.19, U2OS, MG63, and 143B. The migration ability of U2OS cell in Vector-NC group and FDX1-OE group by transwell assay without Matrigel (B) and wound healing assay (C). each experiment was repeated three times. *P<0.05, **P<0.01, ***P<0.001*p < 0.05, **p < 0.01, ***p < 0.001, ****p < 0.0001 each experiment was repeated three times.

The Western blotting results showed that compared to the normal osteoblast hFOB1.19, the protein expression level of FDX1 was significantly increased in osteosarcoma cell lines, with the most significant increase observed in MG63 cells (Fig 9A). colony formation assay and CCK-8 assay revealed that overexpression of FDX1 did not affect the viability of osteosarcoma cells (S9 Fig). The results of wound healing assay and transwell assay indicated that FDX1 promoted the migration of osteosarcoma cells (Fig 9B and 9C).

## Discussion

Cuproptosis is reported as a new form of programmed cell death [10]. Moreover, cuproptosis-related genes have been found to be associated with prognosis of malignant tumor and these genes might be potential therapeutic targets [10, 15, 37, 38]. In this study, a total of six key cuproptosis-mitochondrion genes (FDX1, TOMM20, NDUFB9, COX11, MFN2 and ATP6V1E1) associated with prognosis of osteosarcoma were finally identified. Based on these key genes, the risk score of osteosarcoma was developed and further validated. Then, a nomogram model including four predictive factors (gender, age, metastasis and risk score) was constructed and the model presented satisfactory discrimination and calibration. Based on median value of risk score, all patients were divided into two groups. The striking differences of function enrichment and immune status between groups was confirmed, which indicated critical role of cuproptosis-mitochondrion genes in osteosarcoma.

Since cuproptosis is a copper-triggered modality of mitochondrial cell death, 33 cuproptosis-mitochondrion genes were initially selected after literature review and analysis in STRING database. After Cox regression analysis, six cuproptosis-mitochondrion genes were identified. Among them, FDX1, TOMM20 and NDUFB9 were associated with poor survival of osteosarcoma while COX11, MFN2 and ATP6V1E1 predicted good. In tumor studies, FDX1 has been found to affect prognosis and mediate glucose metabolism, fatty acid oxidation and amino acid metabolism in lung adenocarcinoma, as well as to influence alterations in major pathways in renal cell carcinoma [39, 40]. Our study found that FDX1 was associated with poor prognosis, suggesting FDX1 might participate in tumorigenesis and development of osteosarcoma. According to some research, FDX1 was strongly expressed in osteosarcoma tissue, and patients who had high levels of FDX1 expression had a bad prognosis [41, 42]. Furthermore, there are no literature findings on FDX1 migration and proliferation in osteosarcoma. We conducted some functional experiments and found that FDX1 mainly involves the migration of osteosarcoma and didn’t affect the proliferation of osteosarcoma. Therefore, we speculate that the high expression of FDX1 may aggravate the malignancy of osteosarcoma by promoting the migration of osteosarcoma cells. These results indicate that FDX1 may play a certain role in osteosarcoma progress. The prognosis of patients with osteosarcoma may also be impacted by the high expression of FDX1 through a variety of mechanisms. Yao et al. revealed that high levels of FDX1 expression had been linked to increased tumor cell proliferation and metastasis as well as increased resistance to chemotherapy and radiation treatment [43]. In addition, FDX1 played an important role in regulating cellular oxidative stress response, and its high expression could lead to dysregulation of intracellular redox homeostasis, thus increasing cellular sensitivity to oxidative stress [44]. Together, these mechanisms may be involved in the prognostic impact of FDX1 on osteosarcoma. The expression level of FDX1 was also associated with the immune status. In the results of a study, we saw a correlation between FDX1 expression and infiltration of B cells, CD4T cells, CD8T cells, neutrophils, macrophages and dendritic cells (DCs) in a variety of cancers, suggesting a potential role in the tumor immune microenvironment [45]. And the high expression of FDX1 was associated with suppressive immune microenvironment [45]. However, we are not aware of any study on the immunological aspects of FDX1, TOMM20, NDUFB9, COX11, MFN2 and ATP6V1E1 in osteosarcoma for the time being, which is a direction we need to study subsequently. TOMM20 is the receptor and key subunit of the TOM complex, which has been implicated in many malignancies [46, 47]. A recent study reported that high expression of TOMM20 is associated with cell cycle dysregulation in colorectal cancer, which leading to increased cell proliferation and aggressiveness of cancer cells, indicating a correlation between TOMM20 and poor prognosis in colorectal cancer [48]. High expression of TOMM2 was also related to decreased overall survival and disease-free survival in gastric cancer patients [47]. Furthermore, overexpression of TOMM20 led to aggressiveness, resistance to treatment, and recurrence in spinal cord tumors, which was significantly associated with the prognosis of spinal cord tumors [49]. TOMM20 also had some influence on immune cells, such as resting CD4 memory T cell, monocytes and CD8 T cells [50]. These findings suggested that TOMM20 may played a role in tumor immune evasion and resistance to treatment, as well as contribute to the aggressiveness of cancer cells. TOMM20 upregulation may provide tumor cells the ability to avoid immune surveillance and encourage the development of cancer. NDUFB9 is a mitochondrial membrane respiratory chain NADH dehydrogenase accessory component [51]. Down-regulation of NDUFB9 was demonstrated to promote breast cancer cell proliferation and metastasis [19]. A substantial connection between high NDUFB9 expression and CD8+ T cell infiltration was discovered in a study [52]. These findings imply that NDUFB9 may be crucial in controlling the immune response to malignancies. COX11 is involved in the insertion and stabilization of copper ions in the active center of COX, and the deficiency of this gene can lead to a decrease in COX activity, thereby affecting cell energy metabolism and mitochondrial function [53, 54]. Besides, Vincenza Barresi et al. discovered that COX11 was upregulated in coordination with SLC31A1 and SCO1, which was associated with proliferation in colorectal cancer cell [16]. MFN2 and ATP6V1E1 are two genes that associated with mitochondrial dynamics and metal ion transport, which were attribute to cancer development and chemoresistance [55–58]. MFN2 has been identified as a prognostic biomarker associated with immune cell infiltration in kidney renal clear cell carcinoma (KIRC) [59]. Downregulation of MFN2 expression is correlated with poor prognosis in KIRC. And Cheng et al. also observed a significant correlation between MFN2 expression and immune cell infiltration as well as various markers of tumor infiltrating immune cells (TIICs) including multiple immune checkpoints in KIRC tissue [59]. Additionally, previous research has demonstrated that MFN2 inhibits the growth and migration of other human cancer cells by regulating the epithelial-to-mesenchymal transition [60, 61]. Therefore, MFN2 may impact the immune function of osteosarcoma and thus lead to a poorer prognosis. Further investigation is needed to fully understand the underlying mechanisms of MFN2 in tumor immunity and its potential as a therapeutic target in osteosarcoma. The relationship between ATP6V1E1 and immune infiltration levels is then shown by Li et al. [62]. The results showed that the correlation of mRNA expression of ATP6V1E1 with B cell was statistically significant and had significant correlations with infiltrating levels of CD4 T cells. And the mRNA expressions of ATP6V1E1 was obviously related to macrophage [62]. Therefore, dysfunction of ATP6V1E1 may lead to dysregulation of immune responses, contributing to the development of various autoimmune diseases and cancer. All of the aforementioned six cuproptosis-mitochondrion genes were reported to participate in mitochondrial metabolism and cancer progression at the same time. Finally, it was experimentally verified that the expression of FDX1, COX11, MFN2, TOMM20 and NDUFB9 at mRNA levels was elevated in osteosarcoma. Besides, the mRNA expression level of ATP6V1E1 was decreased in osteosarcoma.

In the present study, GO and KEGG enrichment analysis showed that the six cuproptosis-mitochondrion genes were enriched in TCA cycle and regulation of tumor immunity, which are crucial processes in the development and progress of osteosarcoma [63–66]. Therefore, we further conducted immune infiltration analysis and immune gene correlation analysis among different risk groups. Compared with low-risk group, the immune function of high-risk group was significantly suppressed, manifesting great tumor immune escape in high-risk group. Kenichiro et al. concluded that enhance of CD8+ T cells by activating TLR4 signaling pathway could prevent progression of osteosarcoma [67]. Since immune checkpoint inhibitors (ICIs) have been a prominent topic in cancer treatment, the expression of eight ICIs (LAG3, HAVCR2, CD27, TNFRSF14, CTLA4, TMIGD2, TIGIT, and PDCD1LG2) were analyzed between groups in the study. The results confirmed our risk score might be utilized to predict the effect of ICIs in patients with osteosarcoma.

Chemoresistance is the leading cause of the treatment failure in osteosarcoma, which can significantly reduce survival outcomes of patients [68]. To promote the clinical transformation of cuproptosis-mitochondrion genes, the correlation between expression of cuproptosis-mitochondrion genes and sensitivity to anticancer drugs was analyzed. FDX1 was found to be associated with the sensitivity of ifosfamide, which was one of the first-line chemotherapy agent in osteosarcoma [69]. The result provides a new reference for FDX1 in comprehensive treatment and chemotherapy resistance of osteosarcoma. The highlights of this article are as follows: First, a cuproptosis-mitochondrion prognosis model, rather than only a cuproptosis prognostic model, was constructed in our work. In our opinion, the prognostic model created by merging cuproptosis-mitochondrion genes has better validity and accuracy since cuproptosis includes the tricarboxylic acid cycle, which mostly takes place in mitochondria. Second, we have explained the function of cuproptosis hub gene FDX1 in osteosarcoma: FDX1 mainly promotes the migration of osteosarcoma rather than proliferation. There are still some limitations in the research. The sample of the study is relatively small. More data samples and multicenter clinical trials are urgently needed to confirm the clinical value of model. Moreover, further molecular biological experiments are needed to explore the possible mechanisms of cuproptosis-mitochondrion genes in osteosarcoma. We have only conducted functional experimental studies on FDX1 in relation to a prognostic model, while the roles of the other five genes in osteosarcoma have not been thoroughly analyzed. Additionally, we have not performed in vivo animal experiments to further investigate the functions of these genes in osteosarcoma. These limitations highlight the direction for future research, in which we aim to conduct in-depth analyses to explore the mechanisms of action of cuproptosis-mitochondrion genes in osteosarcoma.

## Conclusion

Our study developed a novel risk score with six cuproptosis-mitochondrion genes to predict the survival outcome of osteosarcoma. A nomogram model to predict prognosis was then constructed with good discrimination and calibration. Besides, the close relationship between cuproptosis-mitochondrion genes and immune landscape was revealed. These findings offer helpful insights on survival prediction and individual treatment of osteosarcoma in clinical practice.

## Supporting information

S1 Fig The interaction numbers of nodes between 33 candidate cuproptosis-mitochondrion genes. (TIF)

S2 Fig The survival analysis of six cuproptosis-mitochondrion genes. The survival curves were plotted based on high and low expression of FDX1 (A), COX11 (B), MFN2 (C), TOMM20 (D), NDUFB9 (E) and ATP6V1E1 (F), respectively.
(TIF)

S3 Fig PCA plot of the construction cohort (A) and the validation cohort (B). The t-SNE analysis of the construction cohort (C) and the validation cohort (D).
(TIF)

S4 Fig The patients were divided into high-risk group and low-risk group based on the risk score and subgroup analysis was performed. The survival curves for male patients (A), female patients (B), patients with metastasis (C), patients without metastasis (D), patients with age ≤ 16 (E) and patients with age > 16 (F).
(TIF)

S5 Fig GSVA of biological pathways between the high-risk and low-risk group. (TIF)

S6 Fig The relationship between 22 kinds of immune cells. (TIF)

S7 Fig The ssGSEA score between the high-risk and low-risk group in immune cells (A) and immunological function (B).
(TIF)

S8 Fig Immunotherapy response of risk groups. Sensitivity difference box plots (A-H) and correlation scatter plots (I-P) of drugs that are effective to osteosarcoma samples.
(TIF)

S9 Fig The proliferative effect of FDX1 in U2OS cells using colony formation assay (A) and CCK-8 assay (B). *p < 0.05, **p < 0.01, ***p < 0.001, ****p < 0.0001. each experiment was repeated three times.
(TIF)

S1 Table 33 cuproptosis-mitochondrion genes. (DOCX)

S1 File (ZIP)
