# Supplementary figures and images for: Construction and validation of a novel cuproptosis-mitochondrion prognostic model related with tumor immunity in osteosarcoma
Source: PLoS One. 2023 Jul 5;18(7):e0288180. doi: 10.1371/journal.pone.0288180 (PMC10321638; doi:10.1371/journal.pone.0288180)

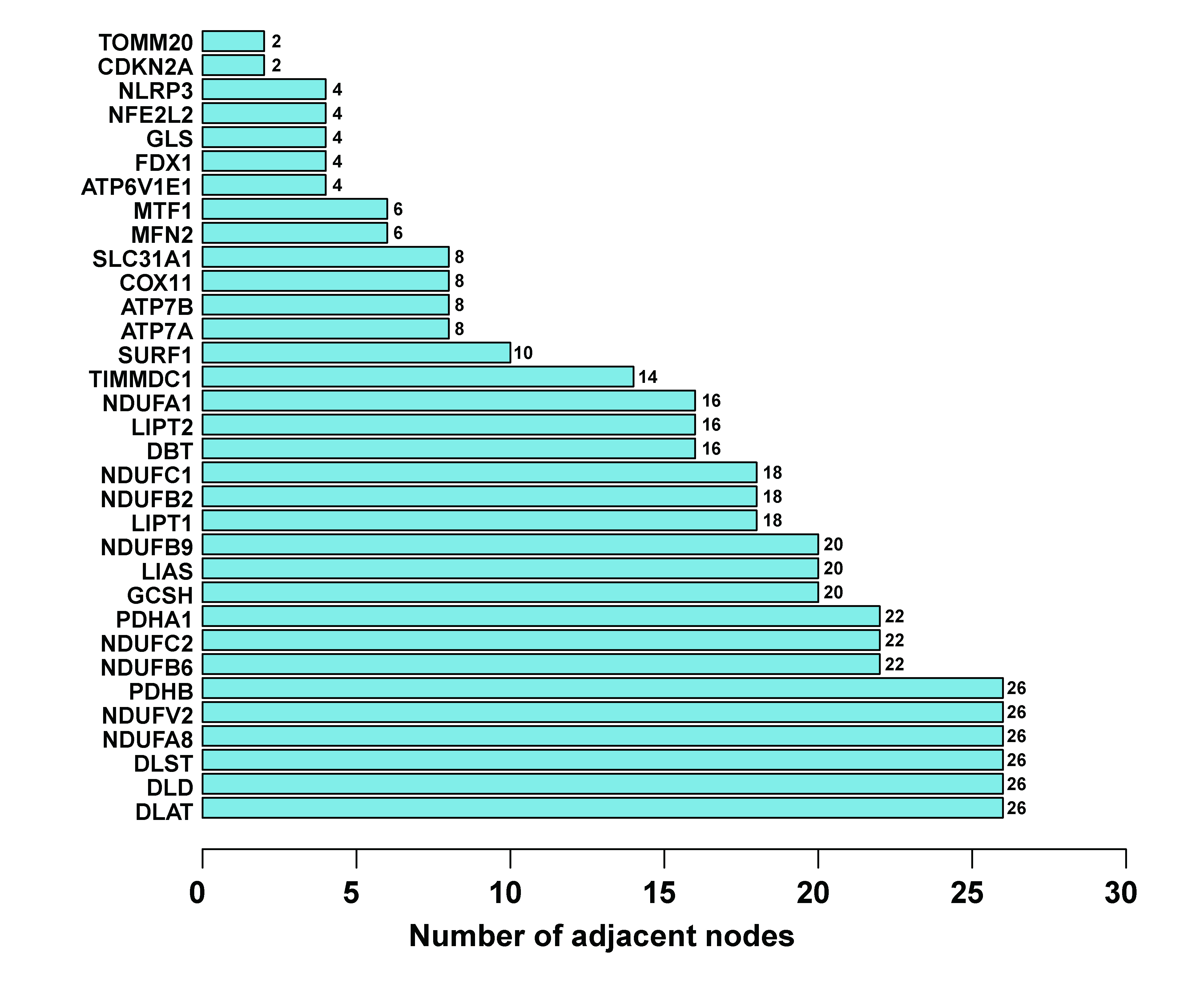

Supplement: S1 Fig — (TIF) [file pone.0288180.s001.tif]

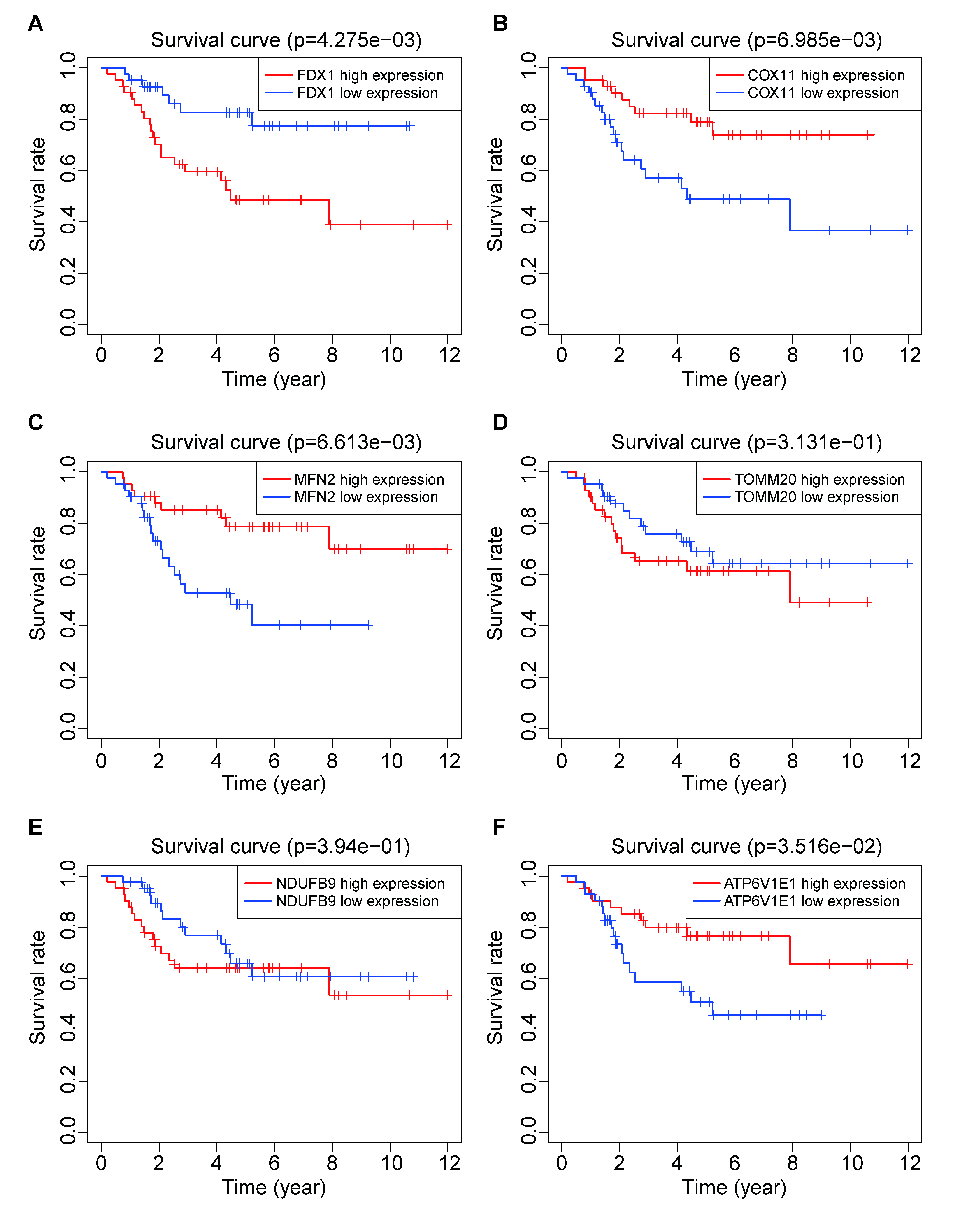

Supplement: S2 Fig — The survival curves were plotted based on high and low expression of FDX1 (A), COX11 (B), MFN2 (C), TOMM20 (D), NDUFB9 (E) and ATP6V1E1 (F), respectively. (TIF) [file pone.0288180.s002.tif]

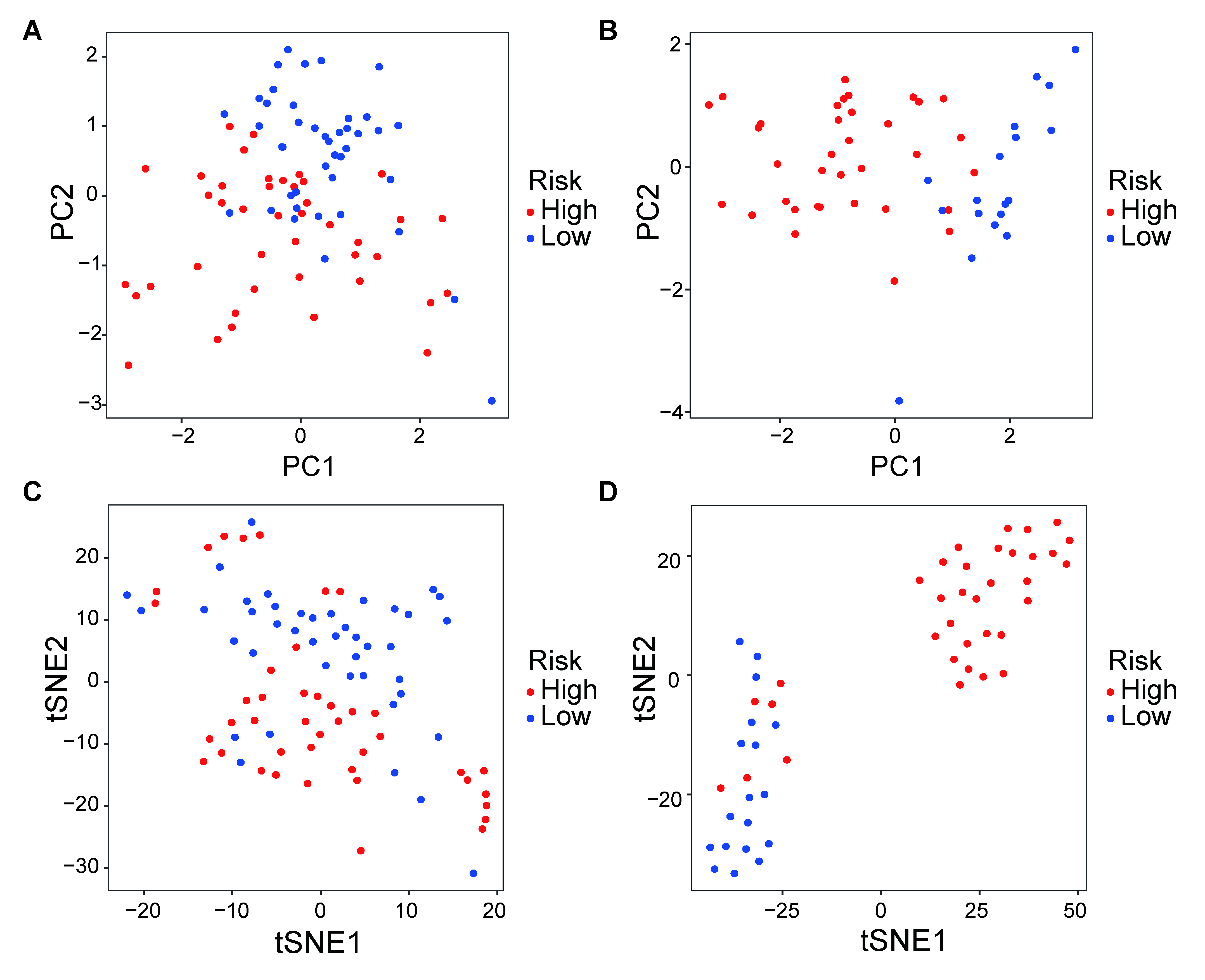

Supplement: S3 Fig — PCA plot of the construction cohort (A) and the validation cohort (B). The t-SNE analysis of the construction cohort (C) and the validation cohort (D). (TIF) [file pone.0288180.s003.tif]

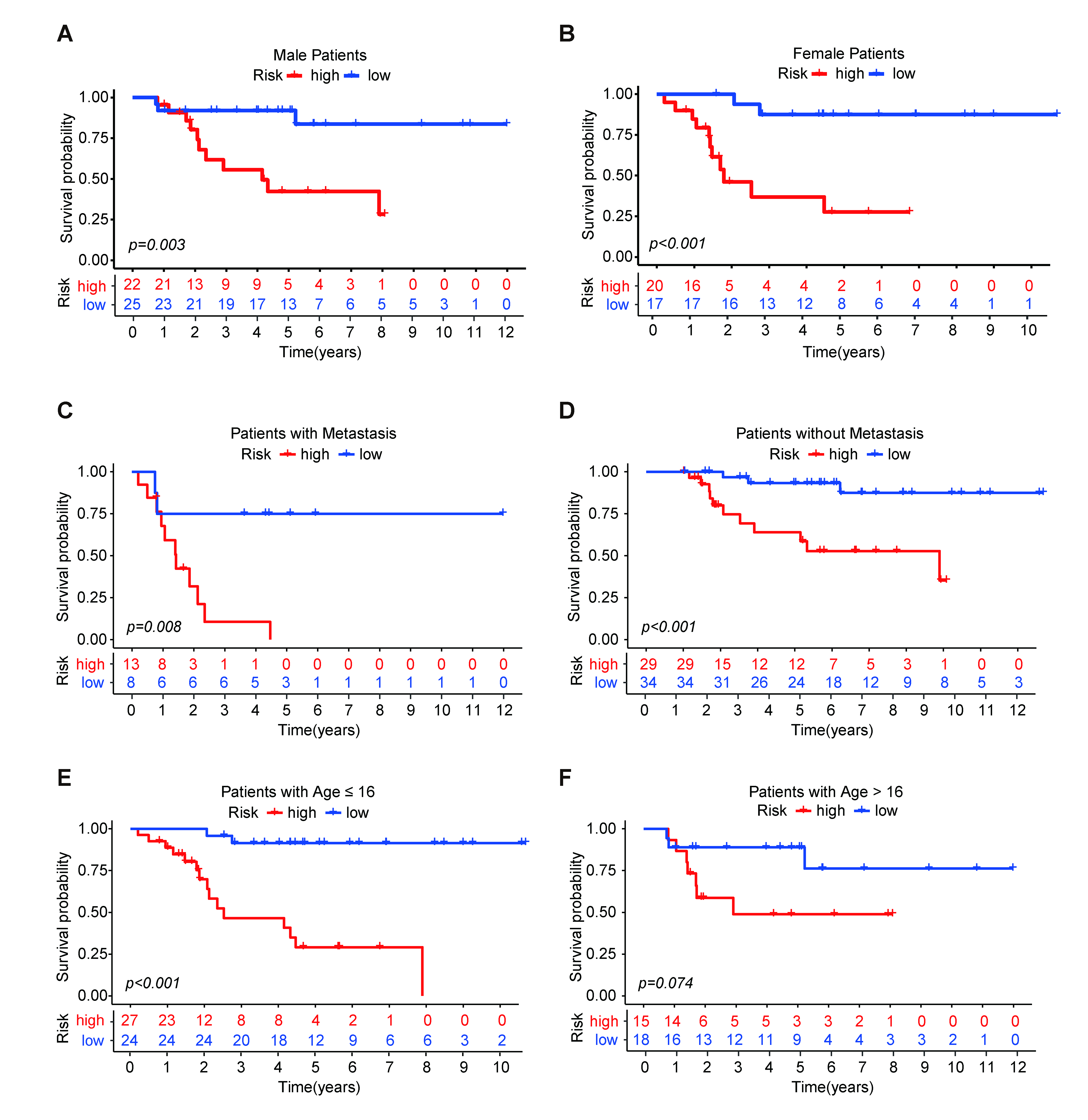

Supplement: S4 Fig — The survival curves for male patients (A), female patients (B), patients with metastasis (C), patients without metastasis (D), patients with age ≤ 16 (E) and patients with age > 16 (F). (TIF) [file pone.0288180.s004.tif]

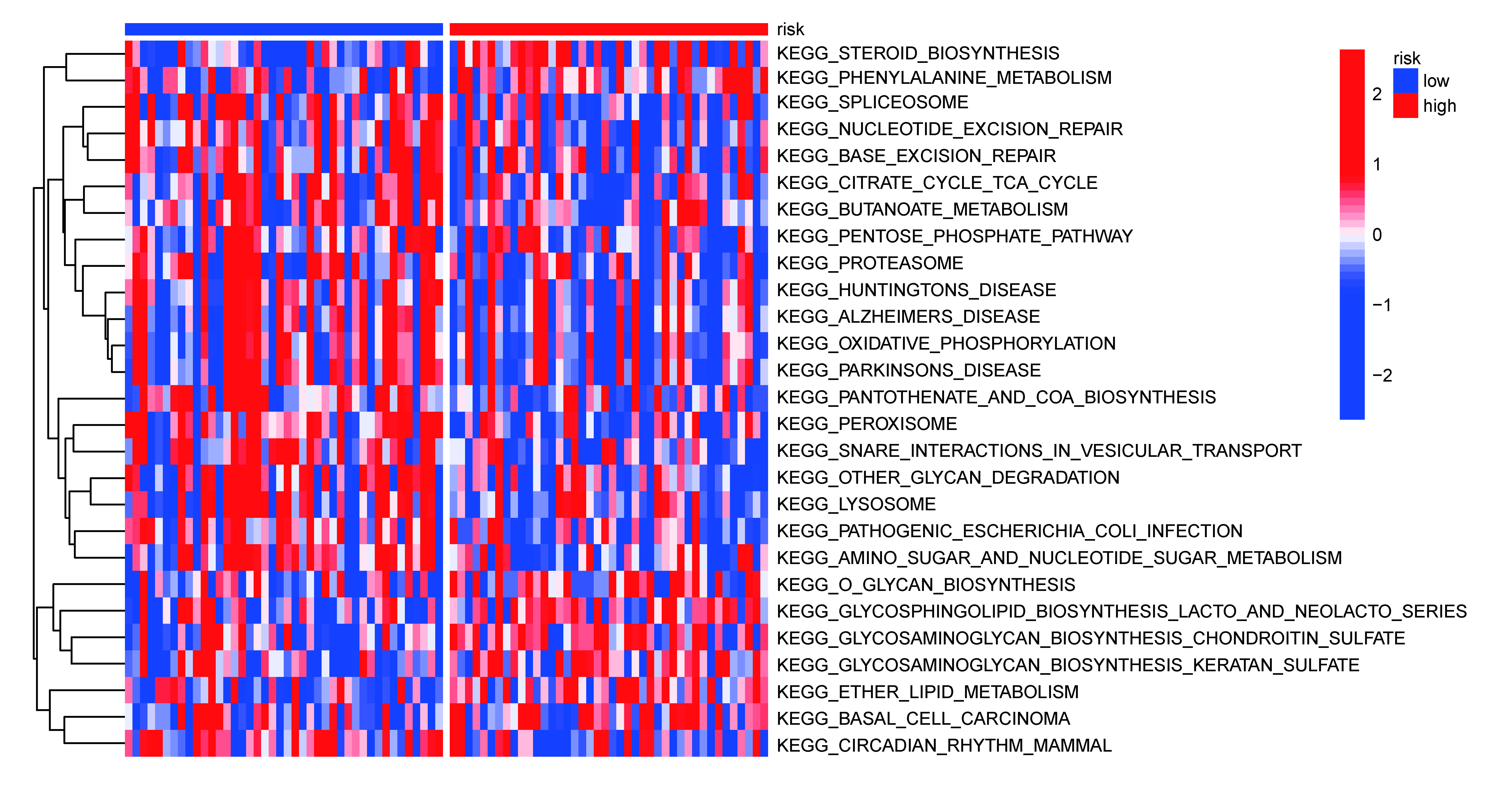

Supplement: S5 Fig — (TIF) [file pone.0288180.s005.tif]

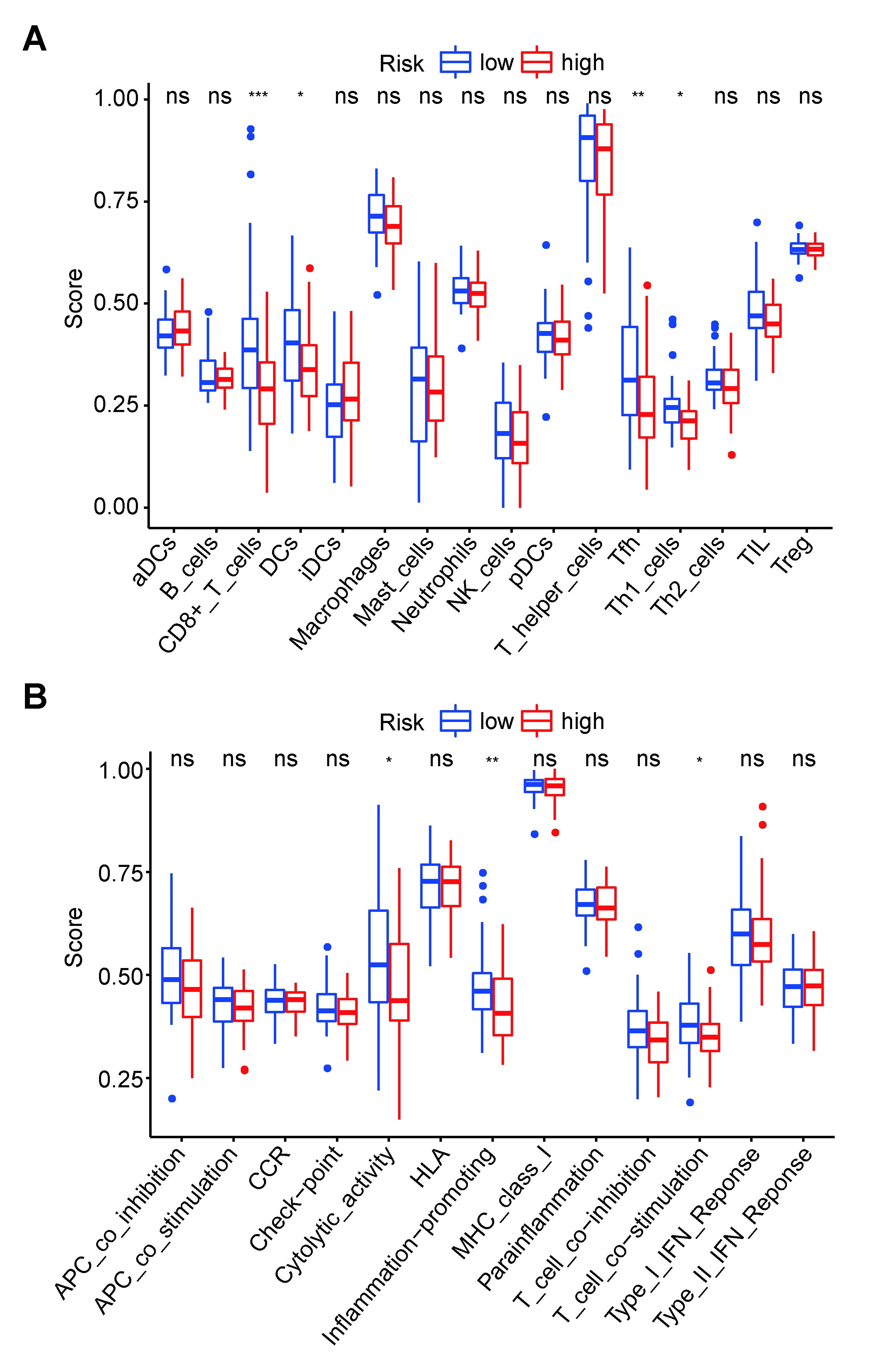

Supplement: S7 Fig — The ssGSEA score between the high-risk and low-risk group in immune cells (A) and immunological function (B). (TIF) [file pone.0288180.s007.tif]

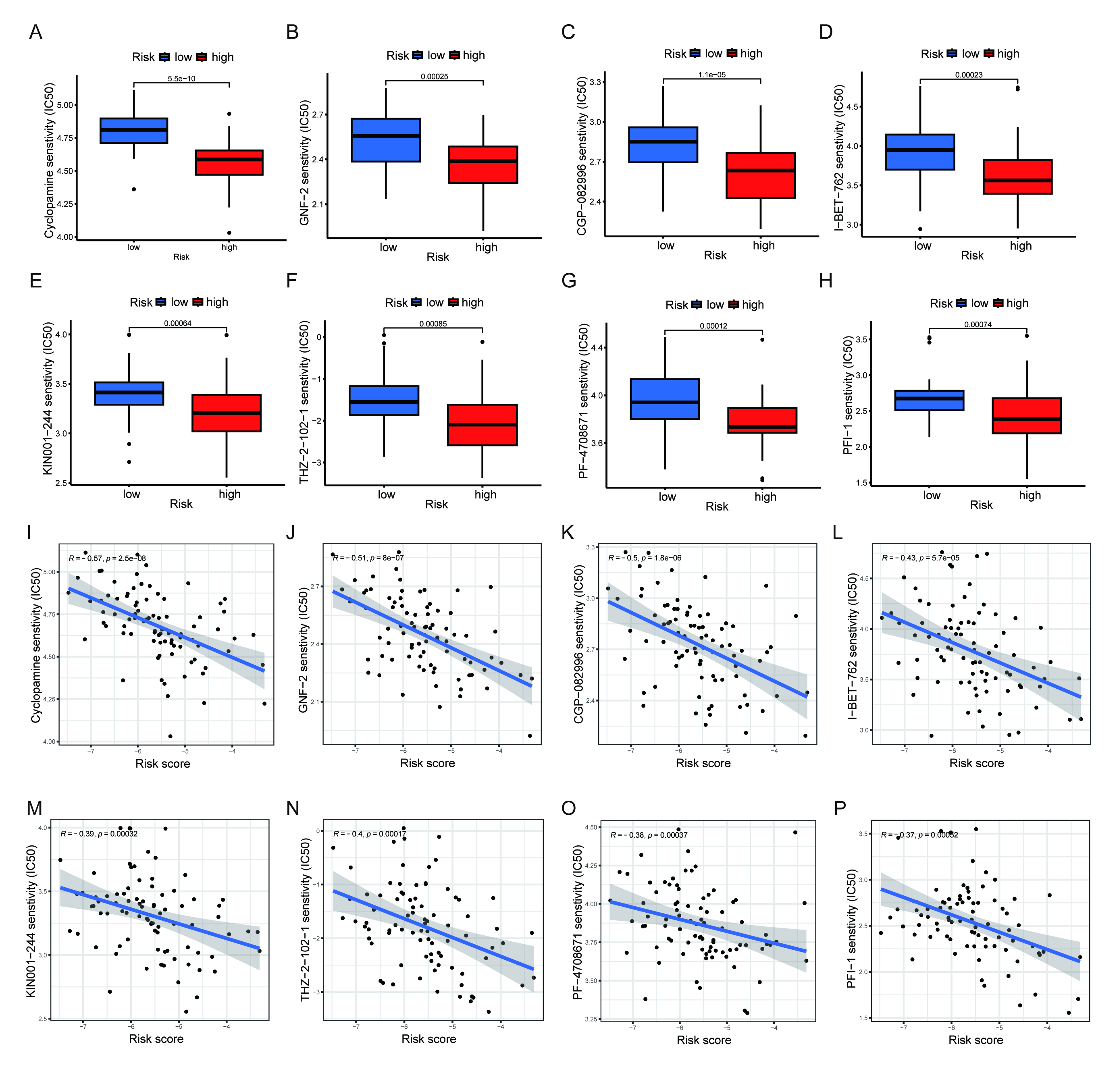

Supplement: S8 Fig — Sensitivity difference box plots (A-H) and correlation scatter plots (I-P) of drugs that are effective to osteosarcoma samples. (TIF) [file pone.0288180.s008.tif]

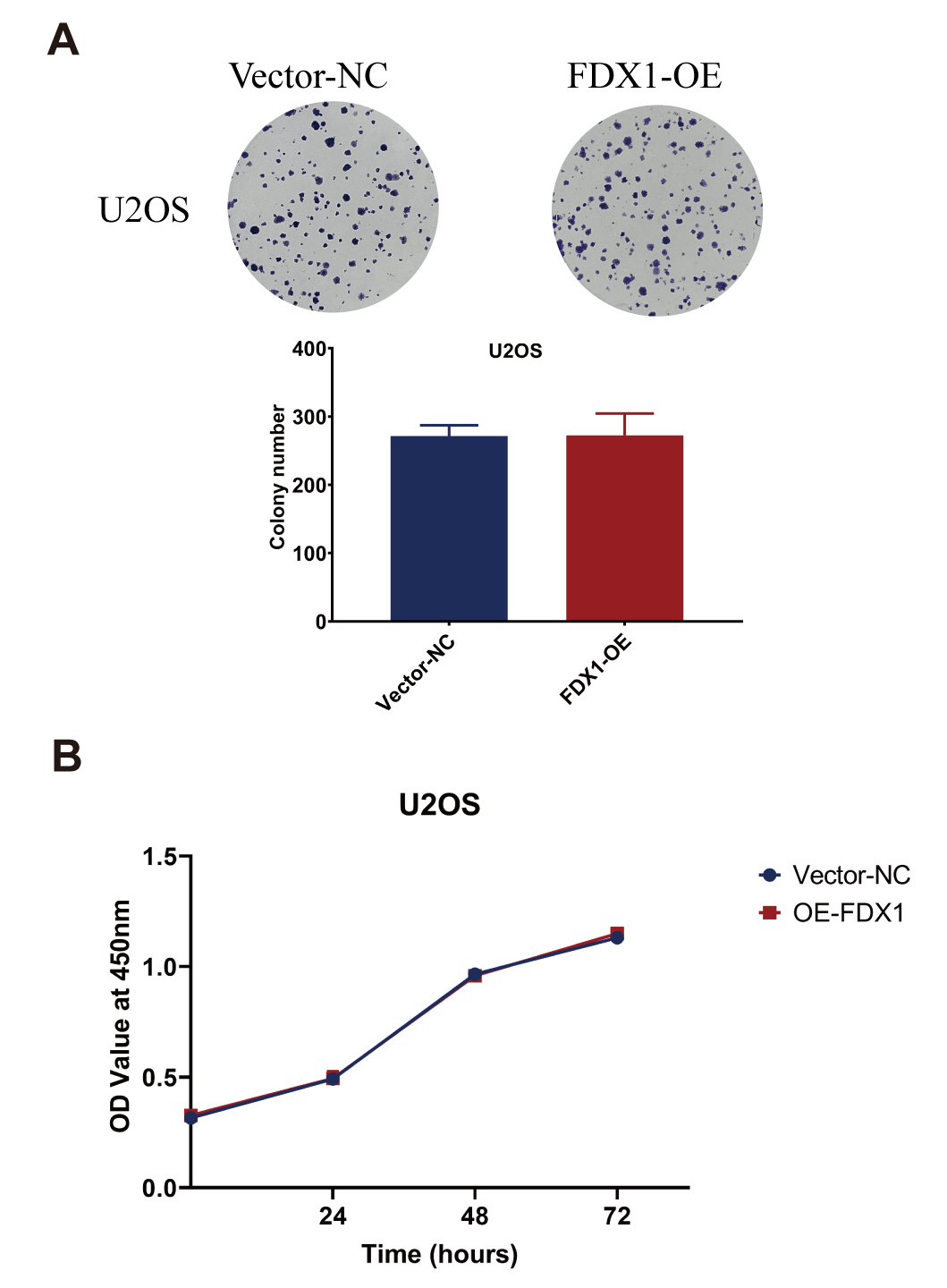

Supplement: S9 Fig — The proliferative effect of FDX1 in U2OS cells using colony formation assay (A) and CCK-8 assay (B). *p < 0.05, **p < 0.01, ***p < 0.001, ****p < 0.0001. each experiment was repeated three times. (TIF) [file pone.0288180.s009.tif]

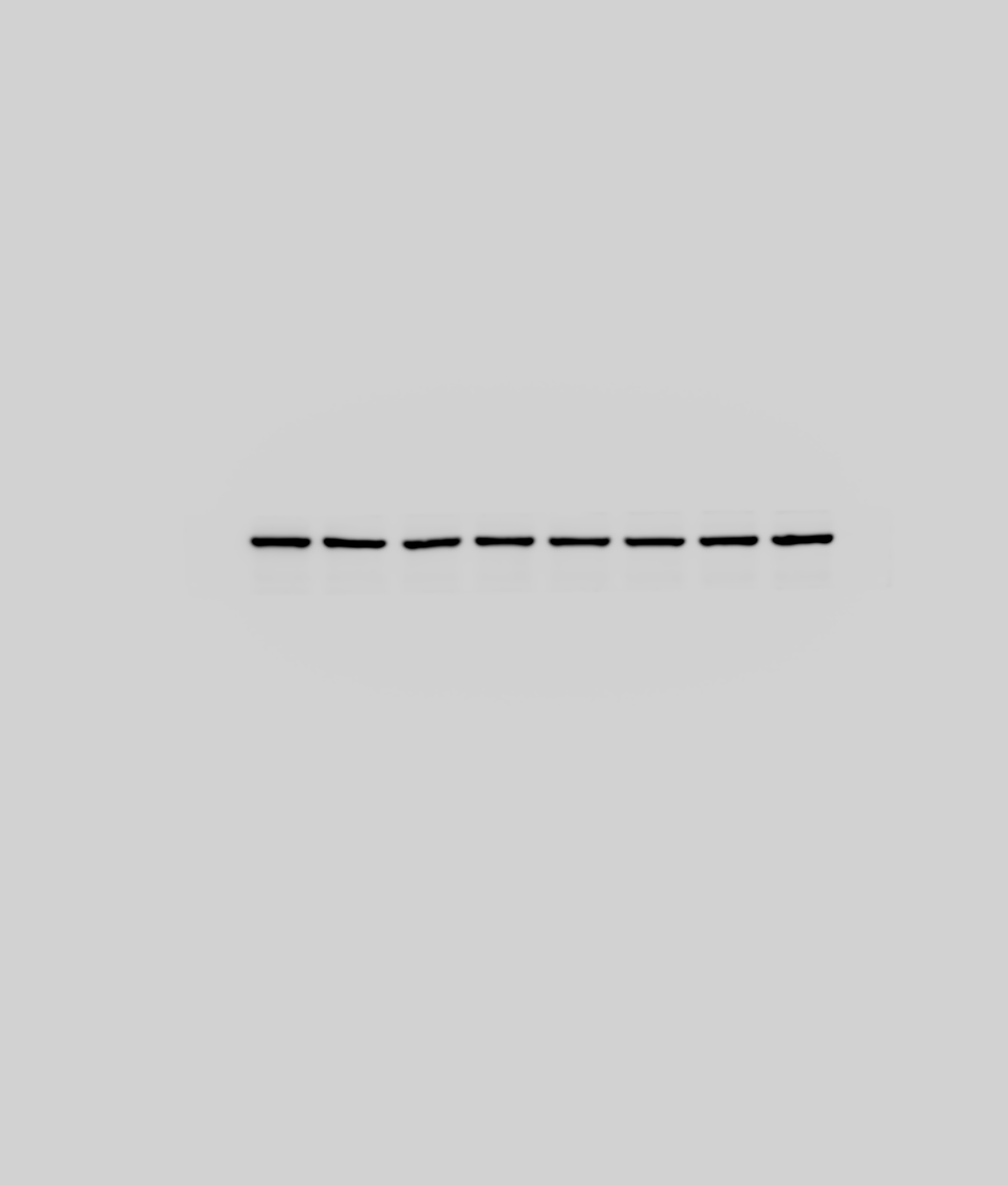

Supplement: S1 File — (ZIP) [file pone.0288180.s011.zip › supplementary Materials/WB/Actin-1sample.tif]

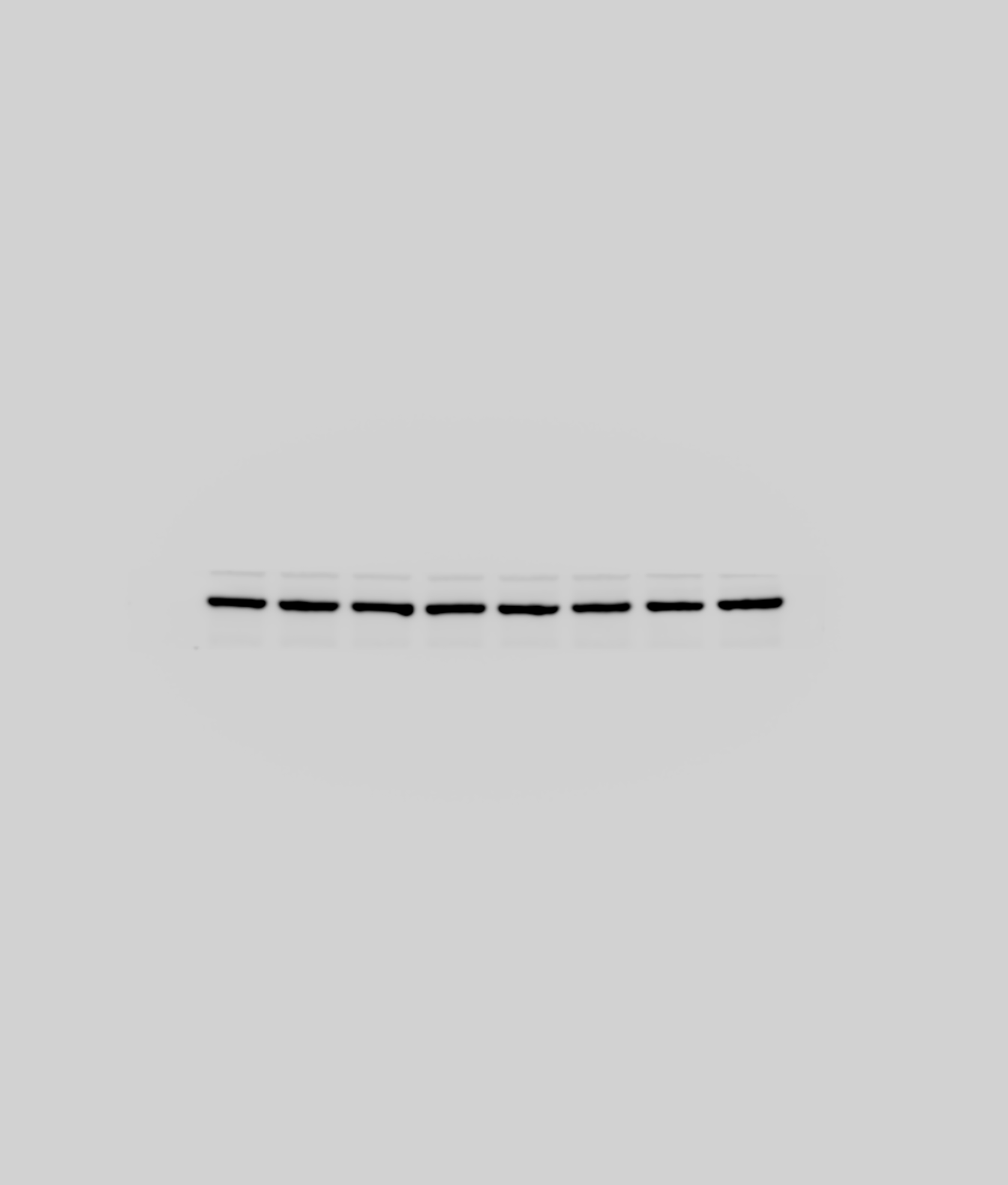

Supplement: S1 File — (ZIP) [file pone.0288180.s011.zip › supplementary Materials/WB/Actin-2.tif]

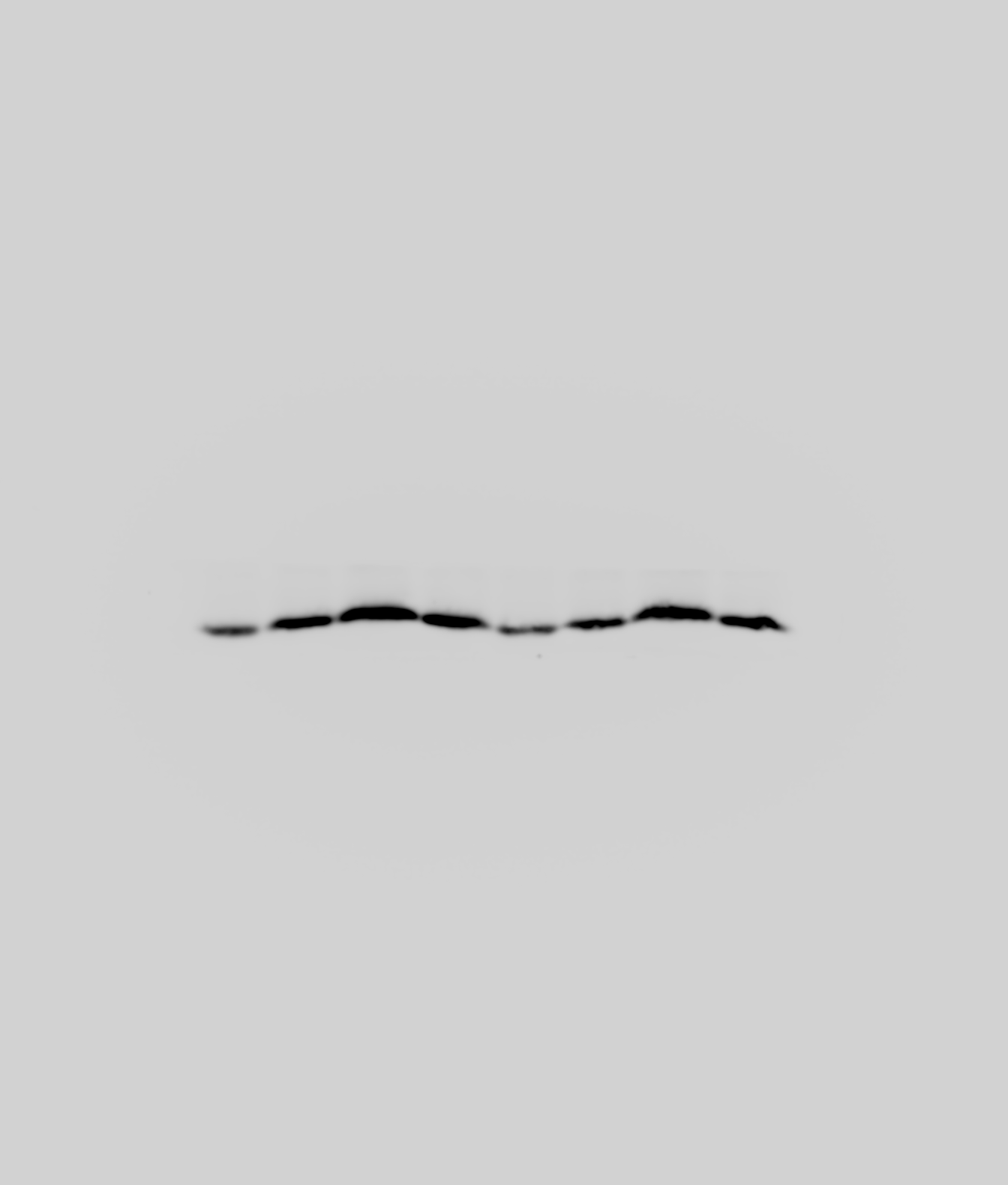

Supplement: S1 File — (ZIP) [file pone.0288180.s011.zip › supplementary Materials/WB/FDX1-1 wb-sample.tif]

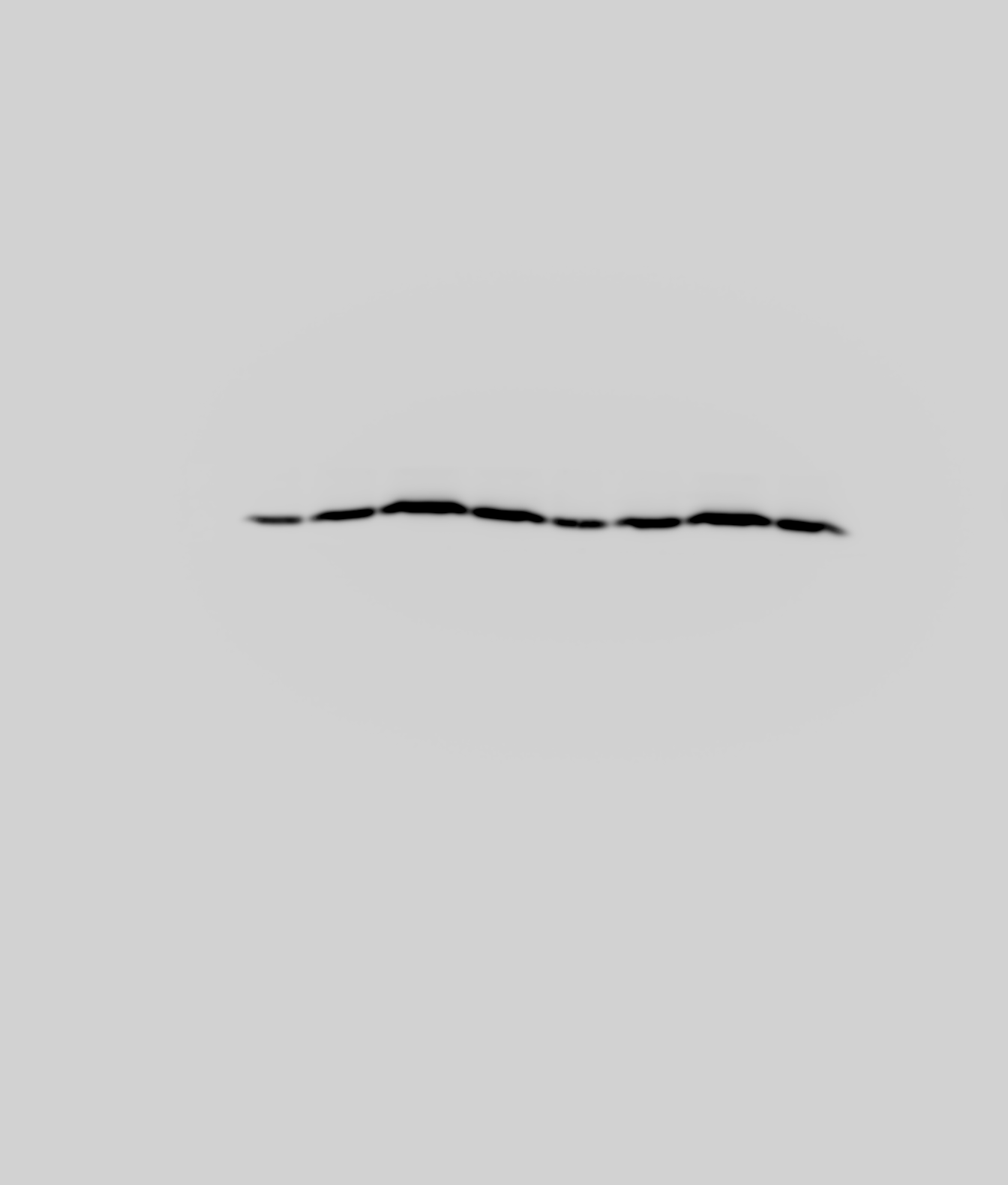

Supplement: S1 File — (ZIP) [file pone.0288180.s011.zip › supplementary Materials/WB/FDX1-2.tif]

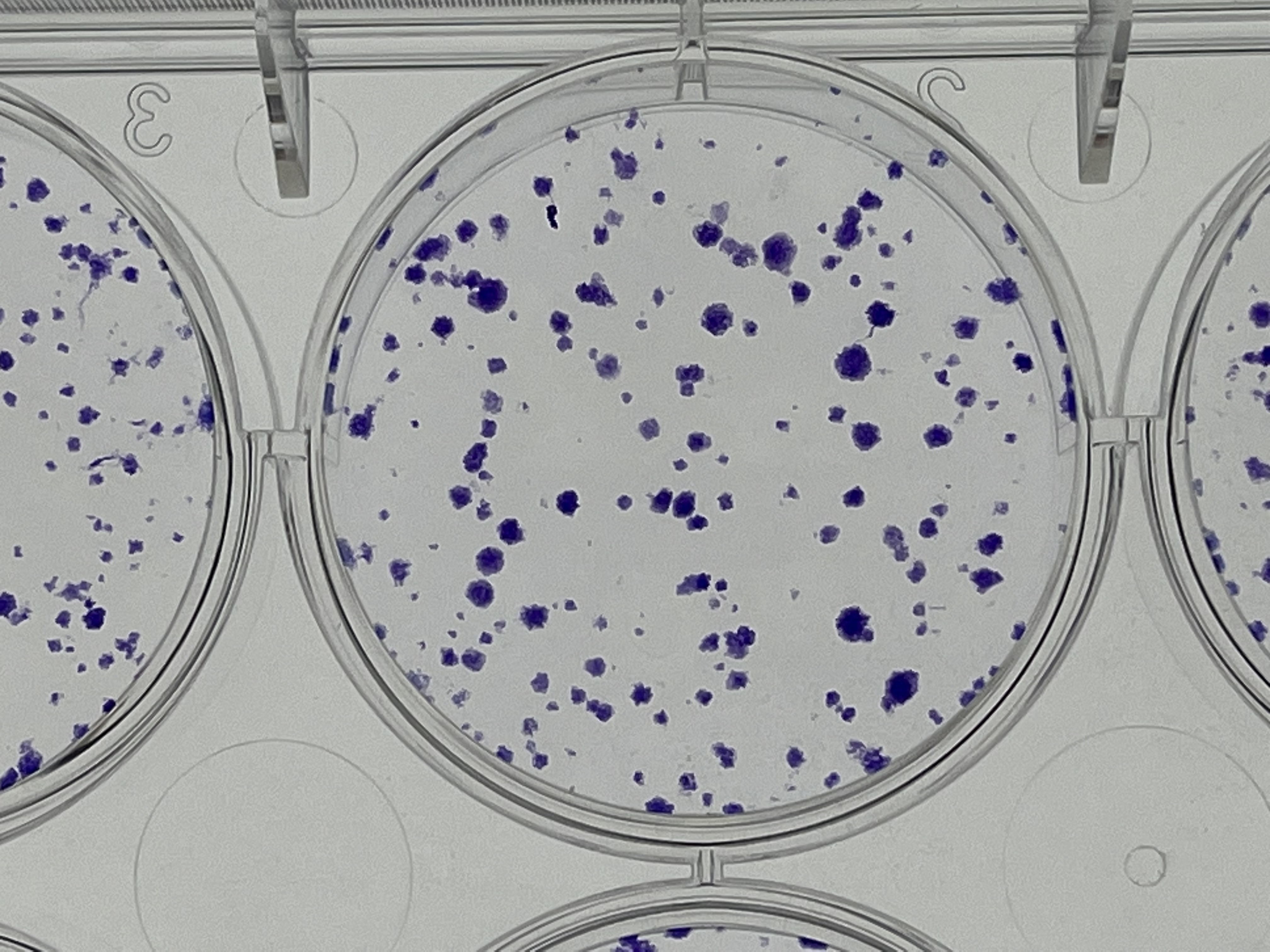

Supplement: S1 File — (ZIP) [file pone.0288180.s011.zip › supplementary Materials/colony formation assay/Repeat 1 U2OS_PCDH_FDX1.jpg]

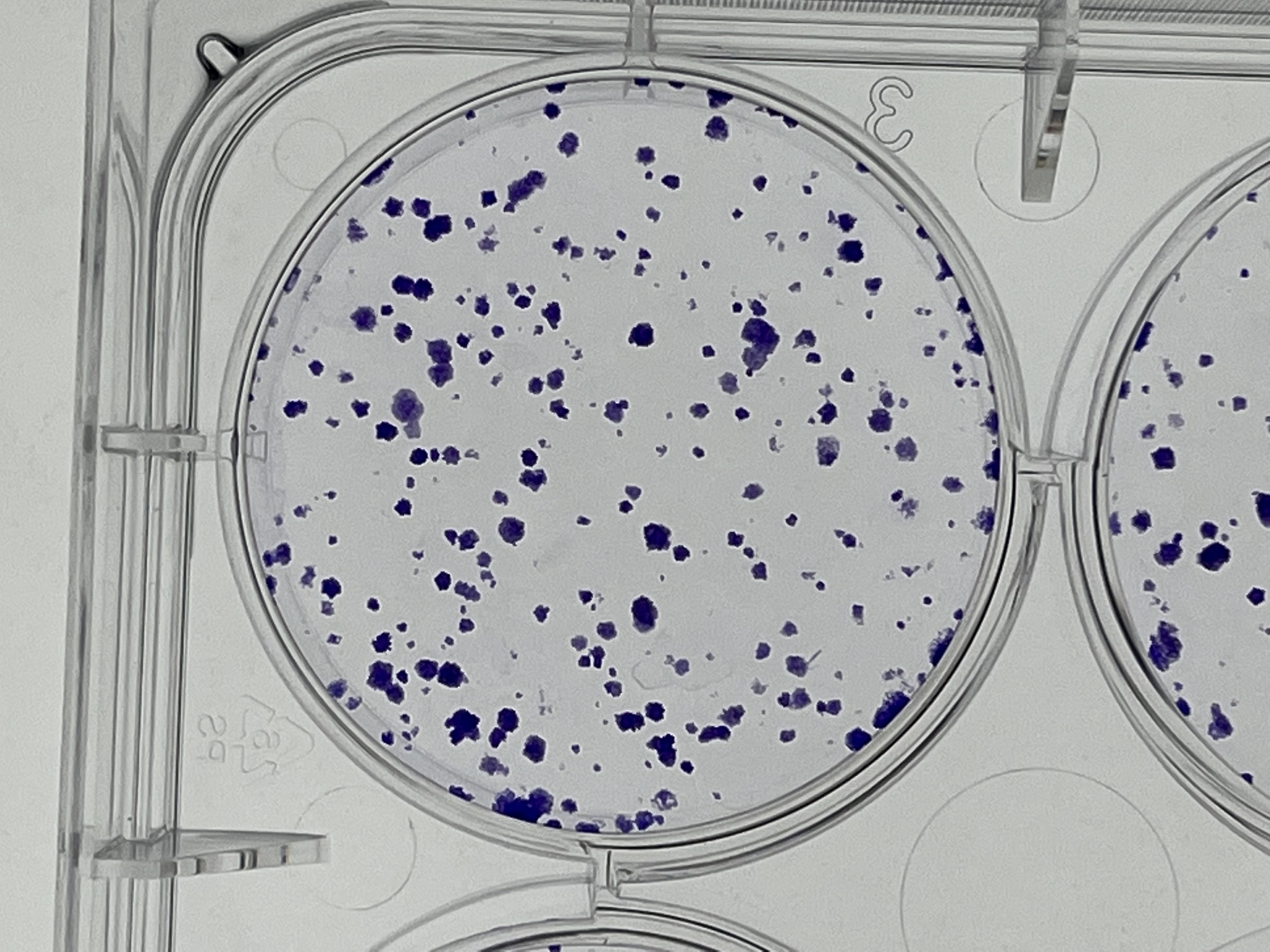

Supplement: S1 File — (ZIP) [file pone.0288180.s011.zip › supplementary Materials/colony formation assay/Repeat 1 U2OS_Veter.jpg]

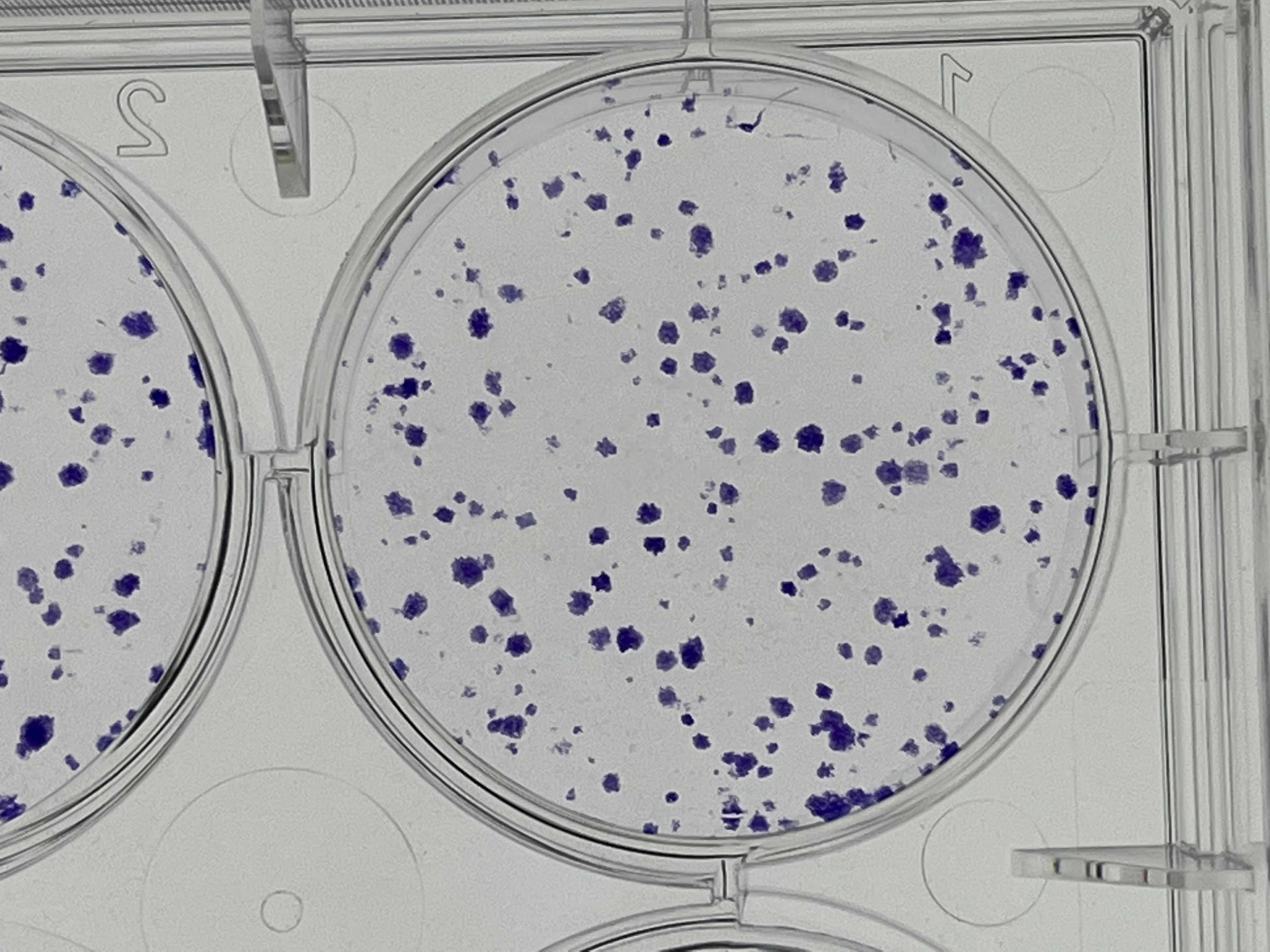

Supplement: S1 File — (ZIP) [file pone.0288180.s011.zip › supplementary Materials/colony formation assay/Repeat 2 U2OS_PCDH_FDX1.jpg]

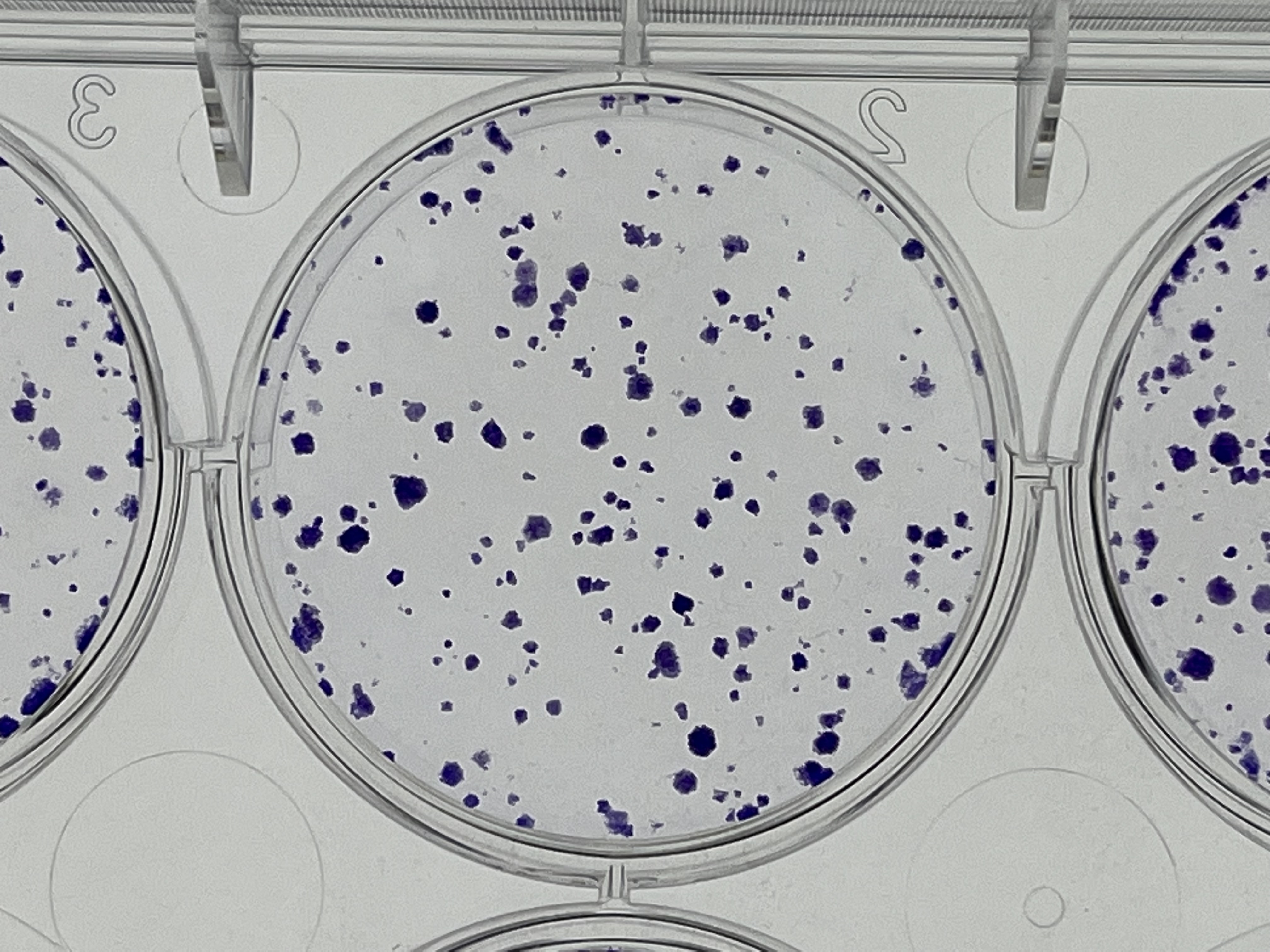

Supplement: S1 File — (ZIP) [file pone.0288180.s011.zip › supplementary Materials/colony formation assay/Repeat 2 U2OS_Veter.jpg]

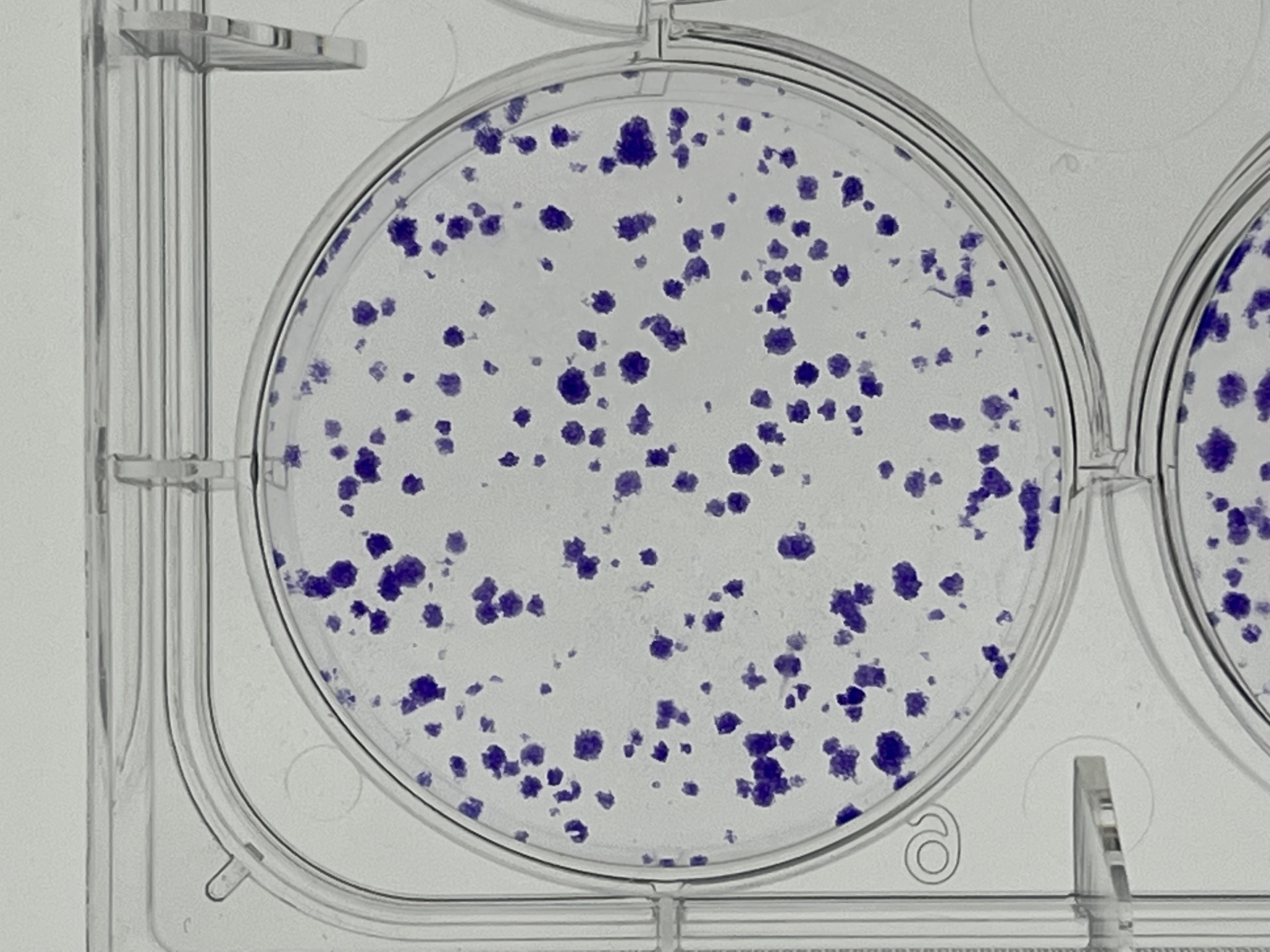

Supplement: S1 File — (ZIP) [file pone.0288180.s011.zip › supplementary Materials/colony formation assay/Repeat 3 U2OS_PCDH_FDX1.jpg]

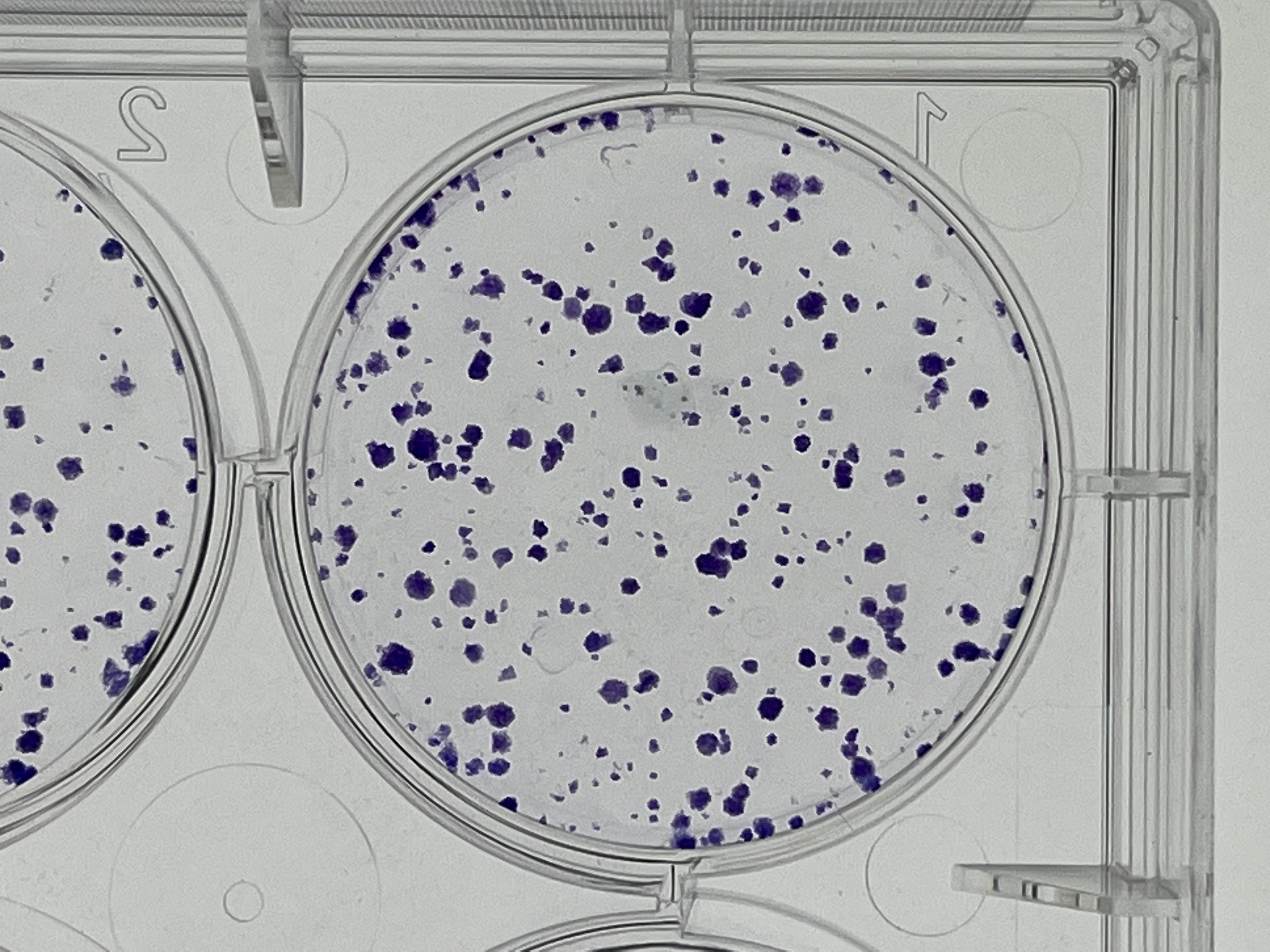

Supplement: S1 File — (ZIP) [file pone.0288180.s011.zip › supplementary Materials/colony formation assay/Repeat 3 U2OS_Veter.jpg]

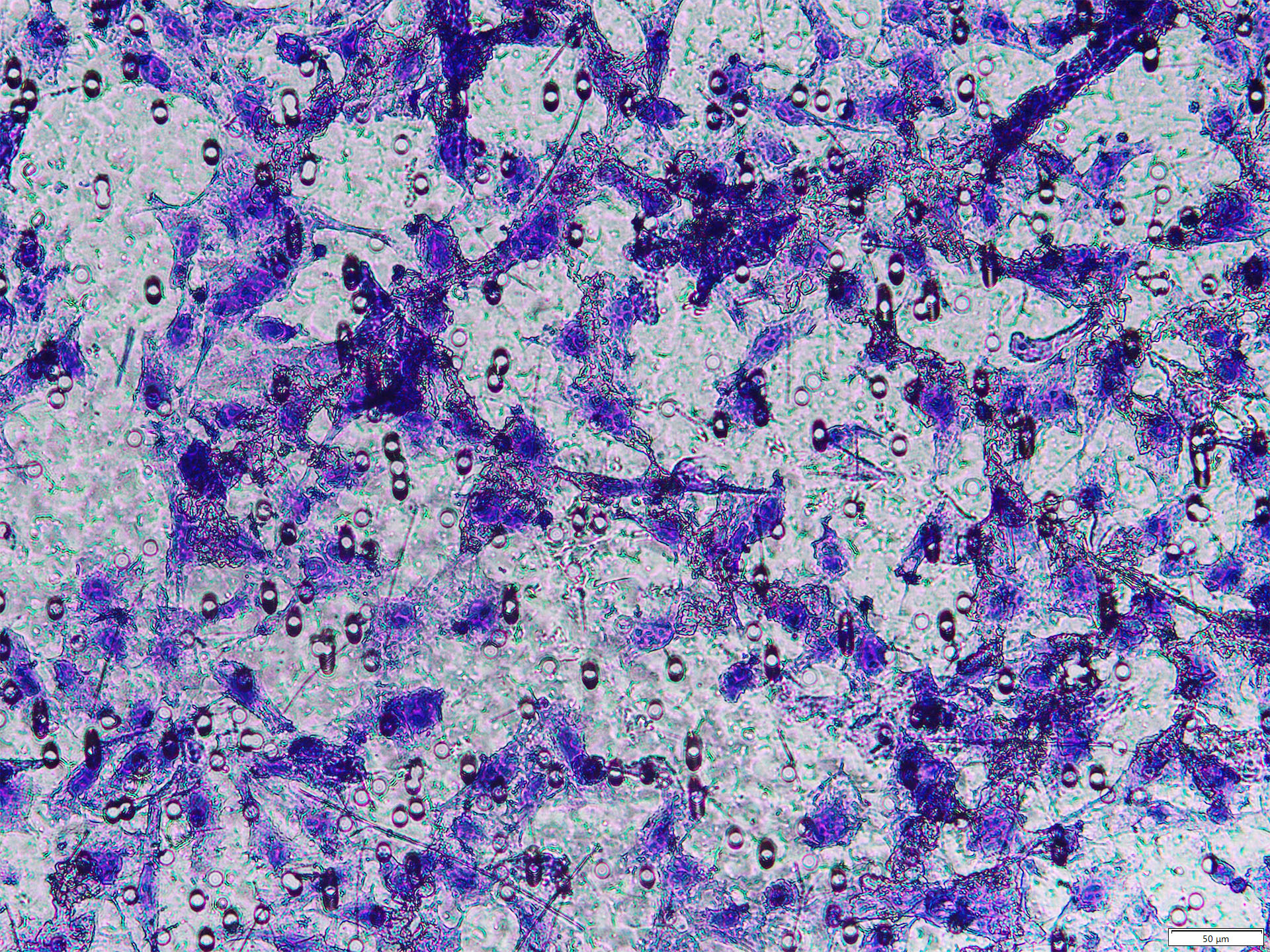

Supplement: S1 File — (ZIP) [file pone.0288180.s011.zip › supplementary Materials/transwell assay/U20S-NC-1.jpg]

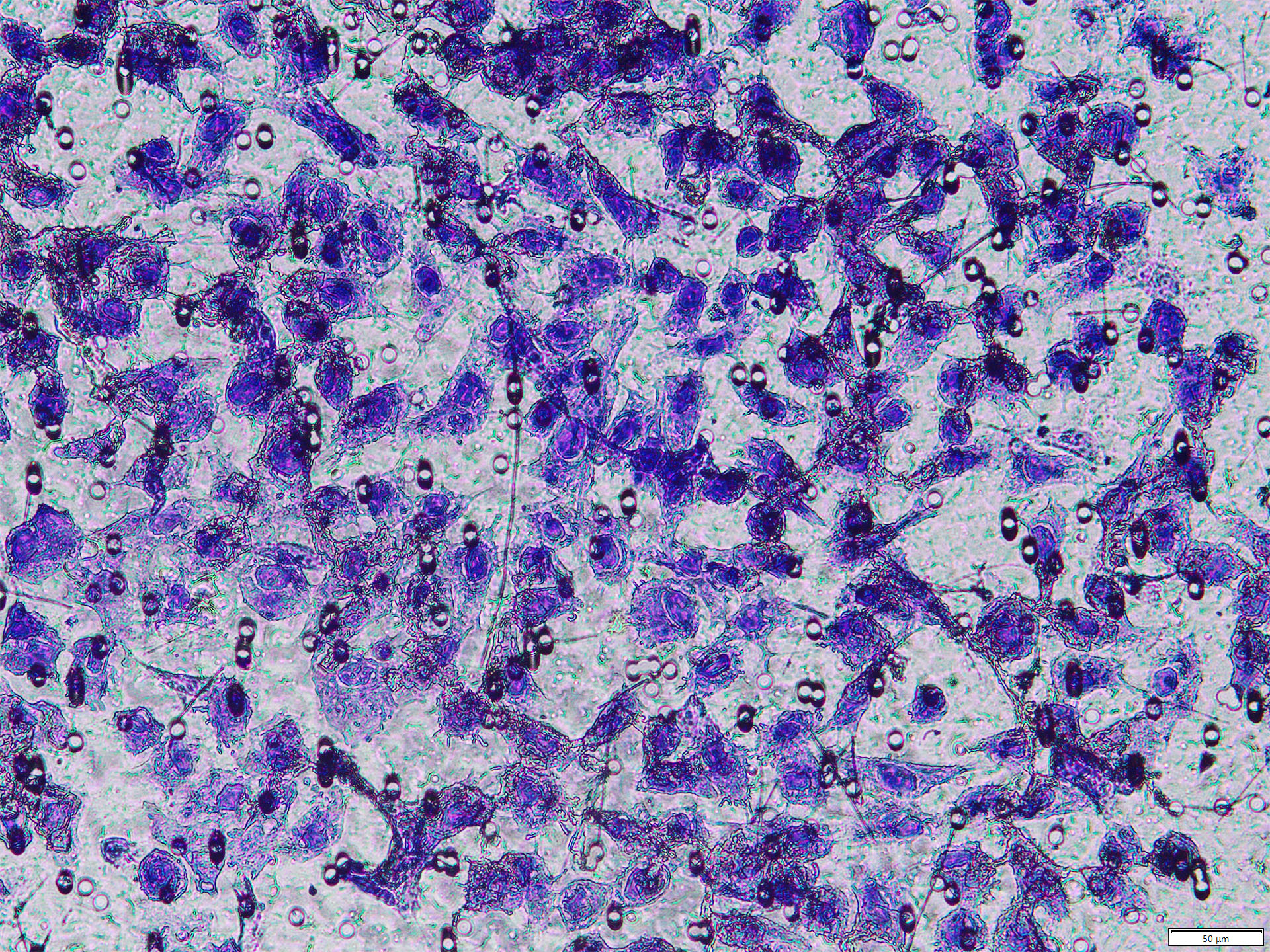

Supplement: S1 File — (ZIP) [file pone.0288180.s011.zip › supplementary Materials/transwell assay/U20S-NC-2.jpg]

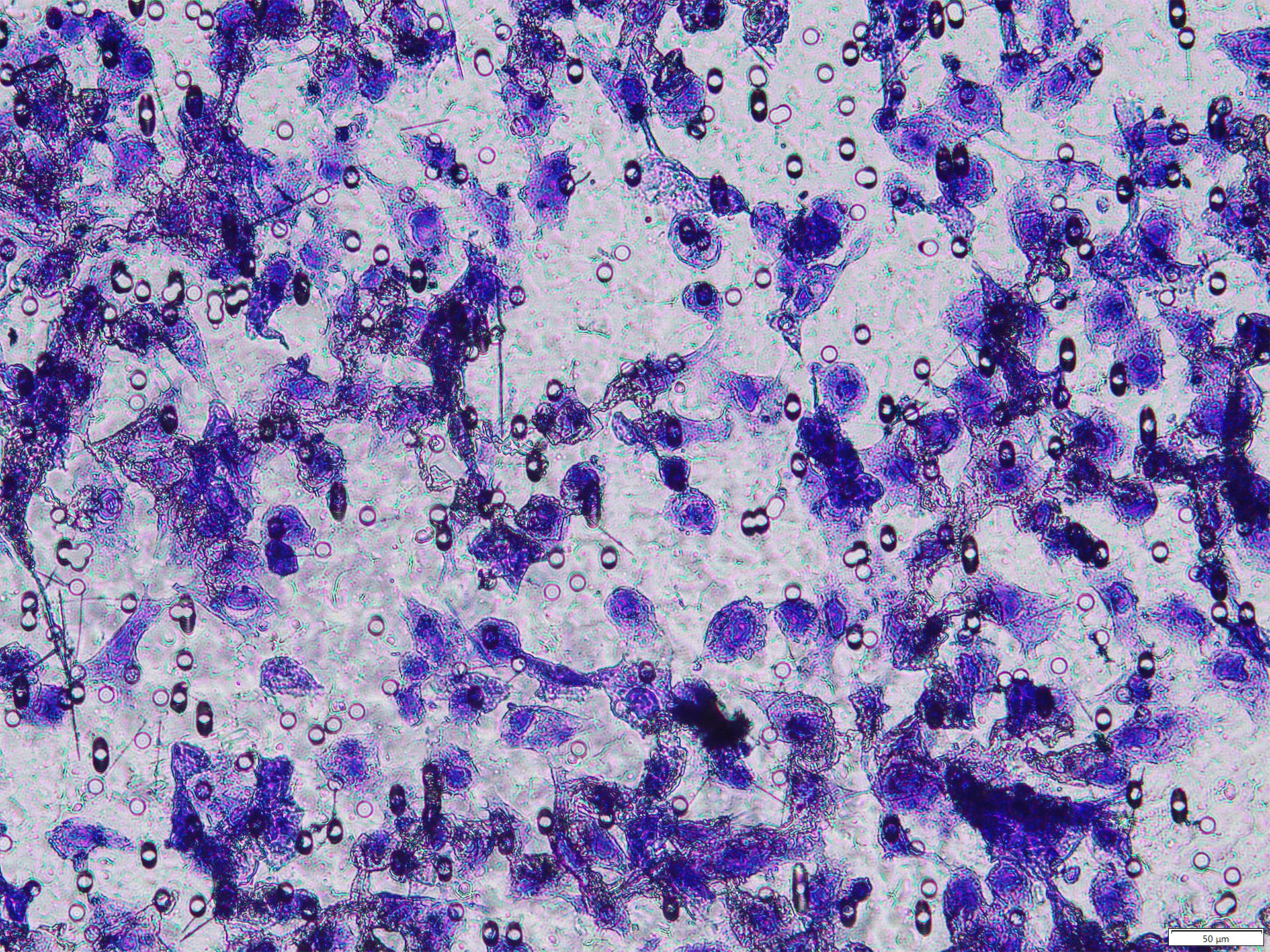

Supplement: S1 File — (ZIP) [file pone.0288180.s011.zip › supplementary Materials/transwell assay/U20S-NC-3.jpg]

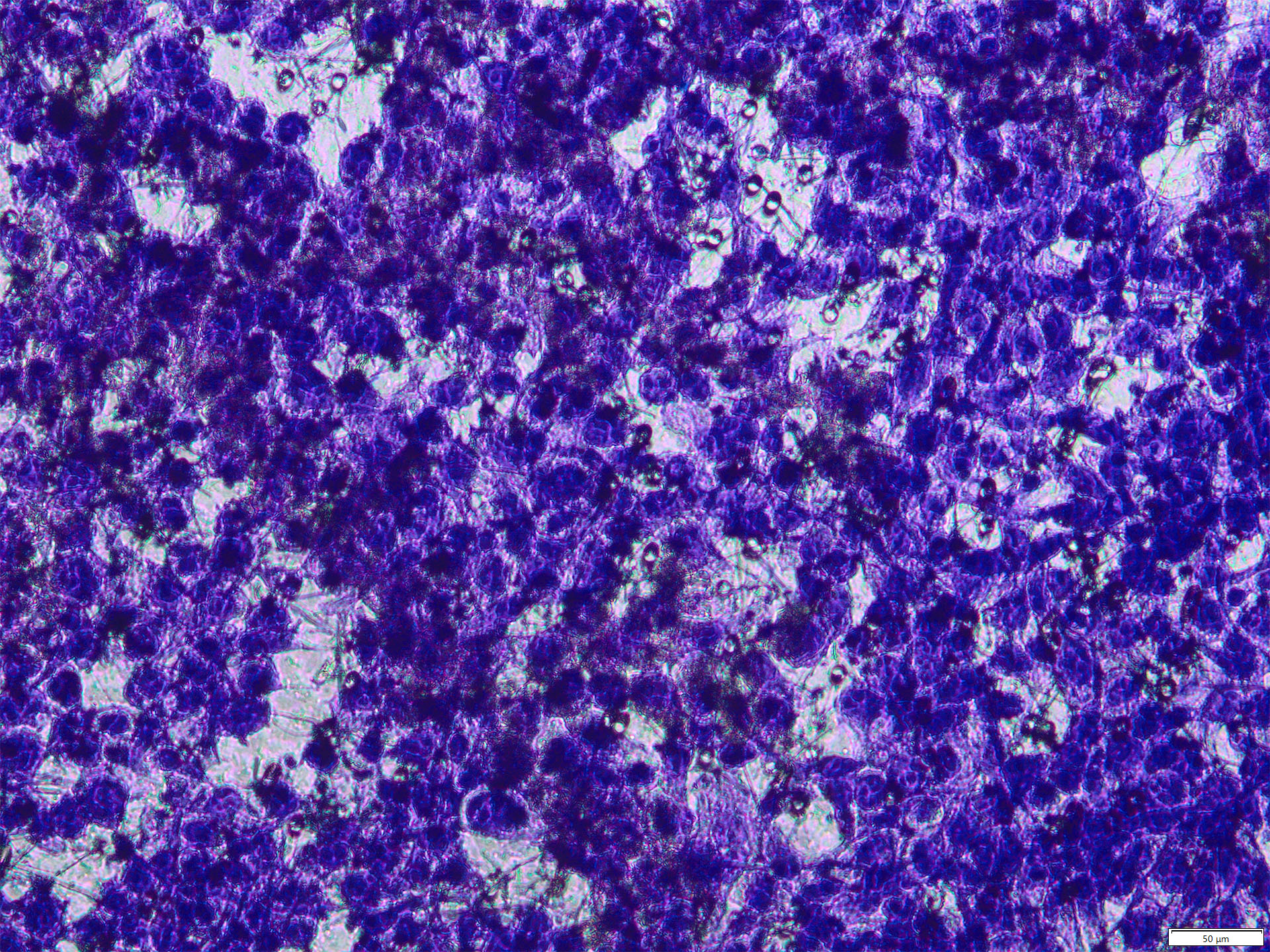

Supplement: S1 File — (ZIP) [file pone.0288180.s011.zip › supplementary Materials/transwell assay/U20S-OE-1.jpg]

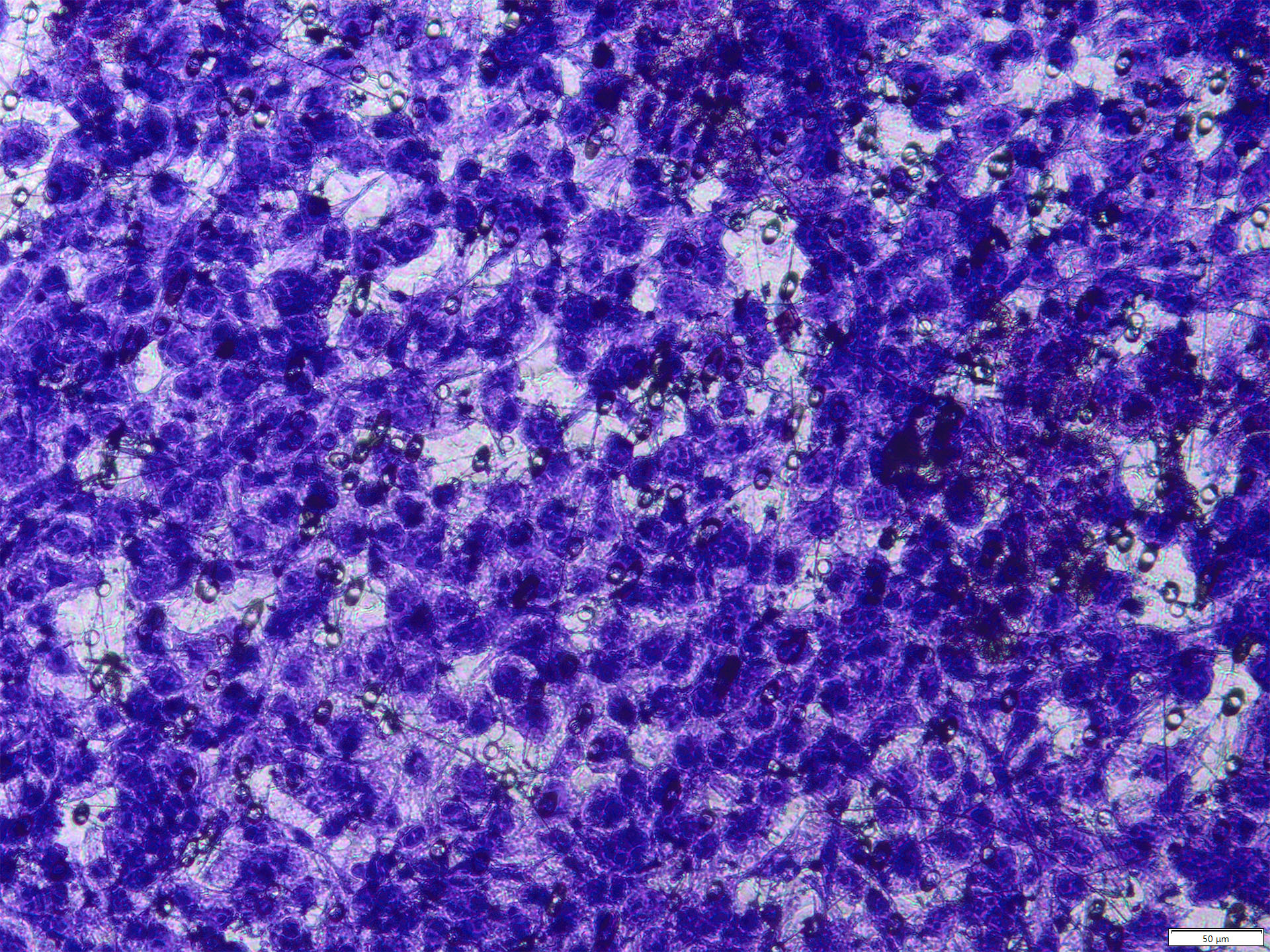

Supplement: S1 File — (ZIP) [file pone.0288180.s011.zip › supplementary Materials/transwell assay/U20S-OE-2.jpg]

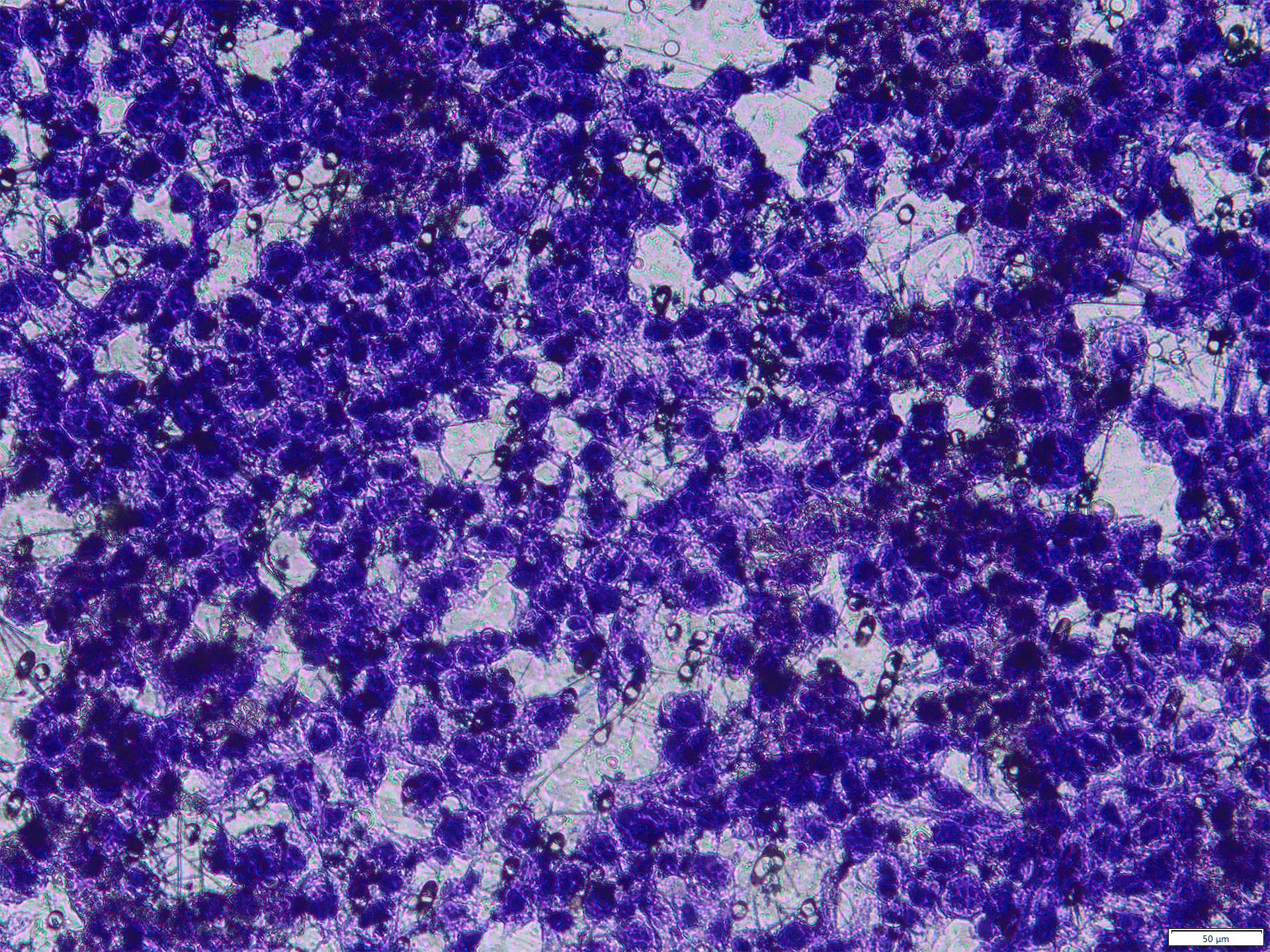

Supplement: S1 File — (ZIP) [file pone.0288180.s011.zip › supplementary Materials/transwell assay/U20S-OE-3.jpg]

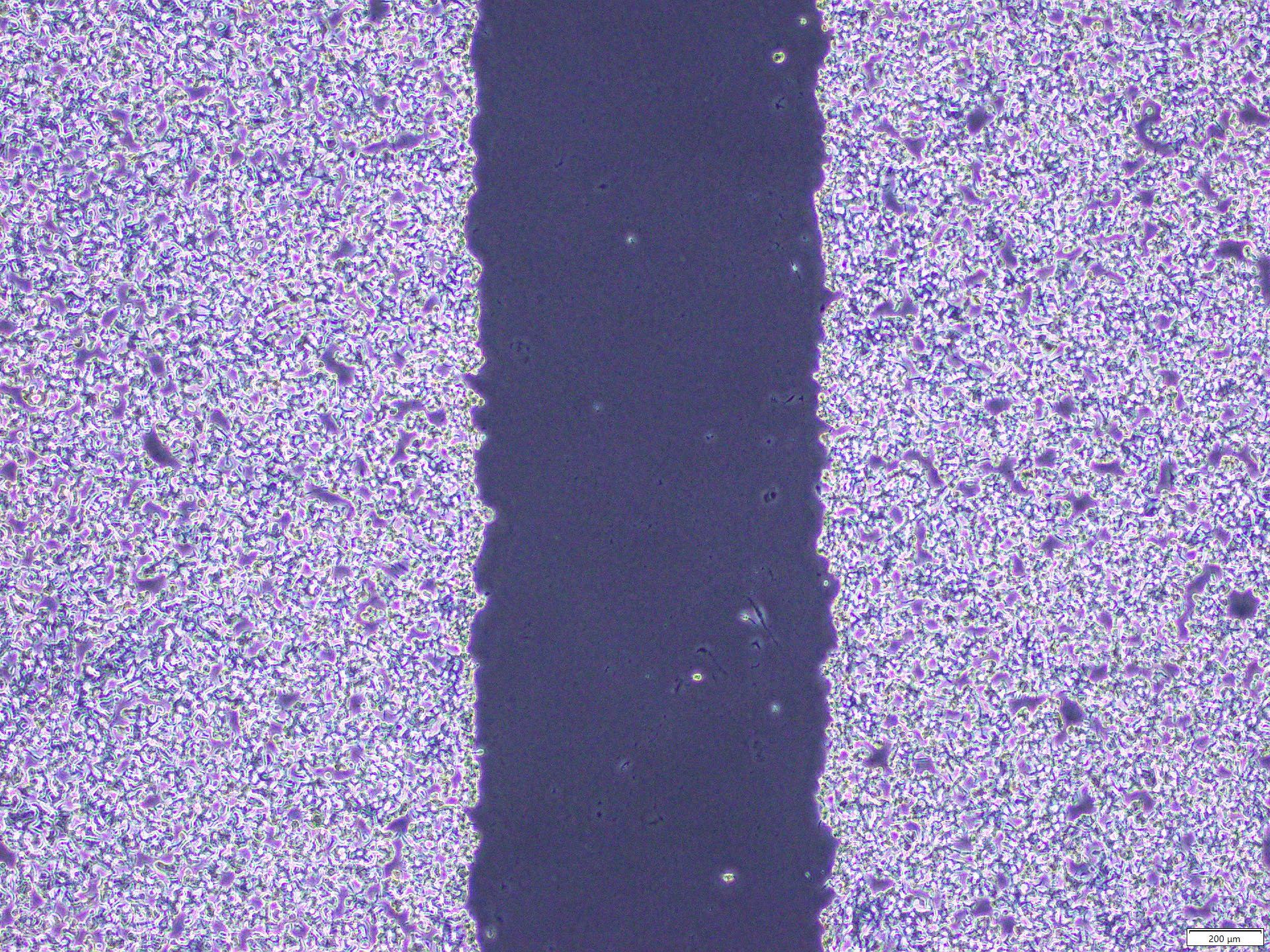

Supplement: S1 File — (ZIP) [file pone.0288180.s011.zip › supplementary Materials/wound healing assay/0h/OE-FDX1-1 U2OS.jpg]

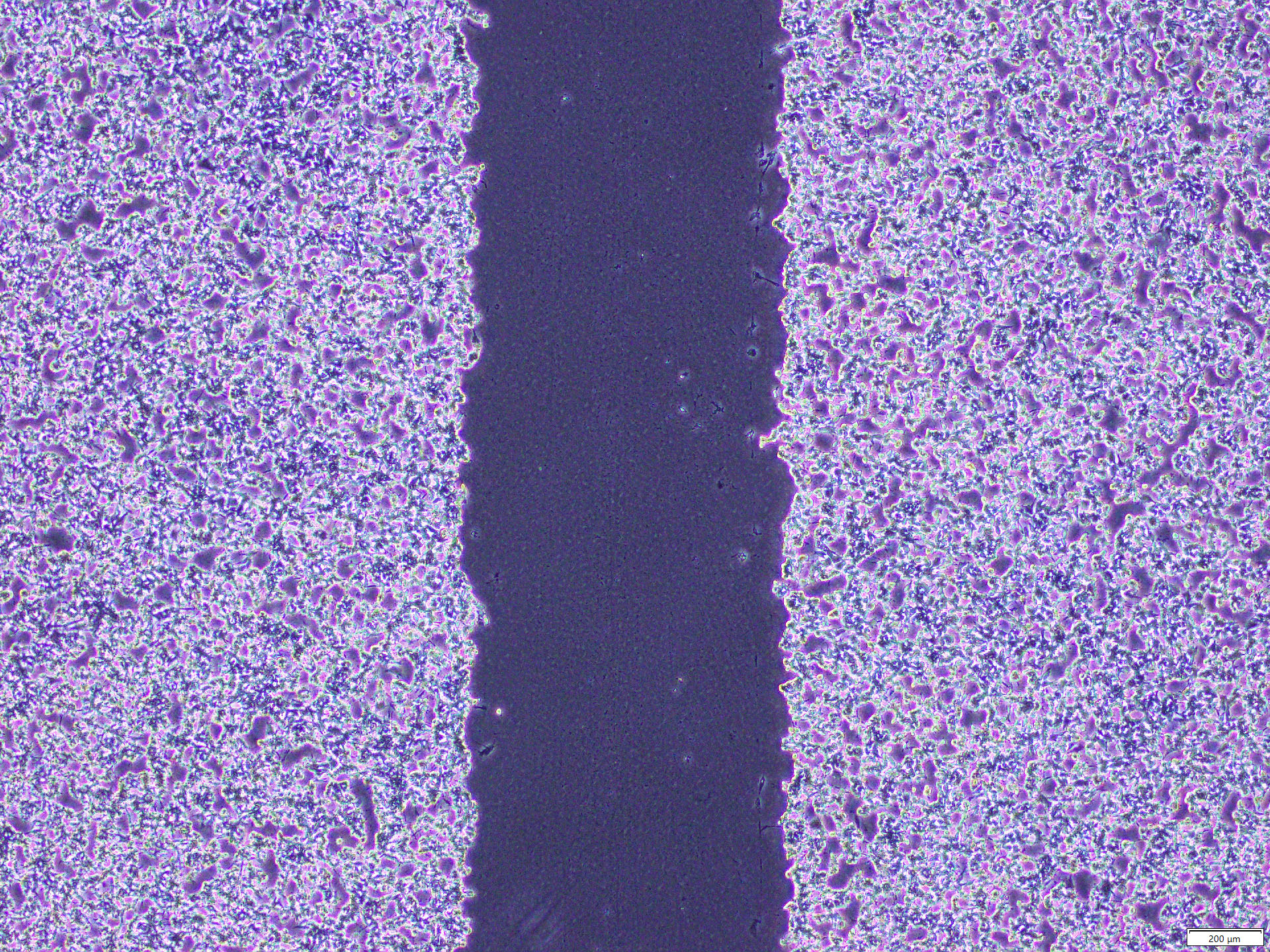

Supplement: S1 File — (ZIP) [file pone.0288180.s011.zip › supplementary Materials/wound healing assay/0h/OE-FDX1-2 U2OS.jpg]

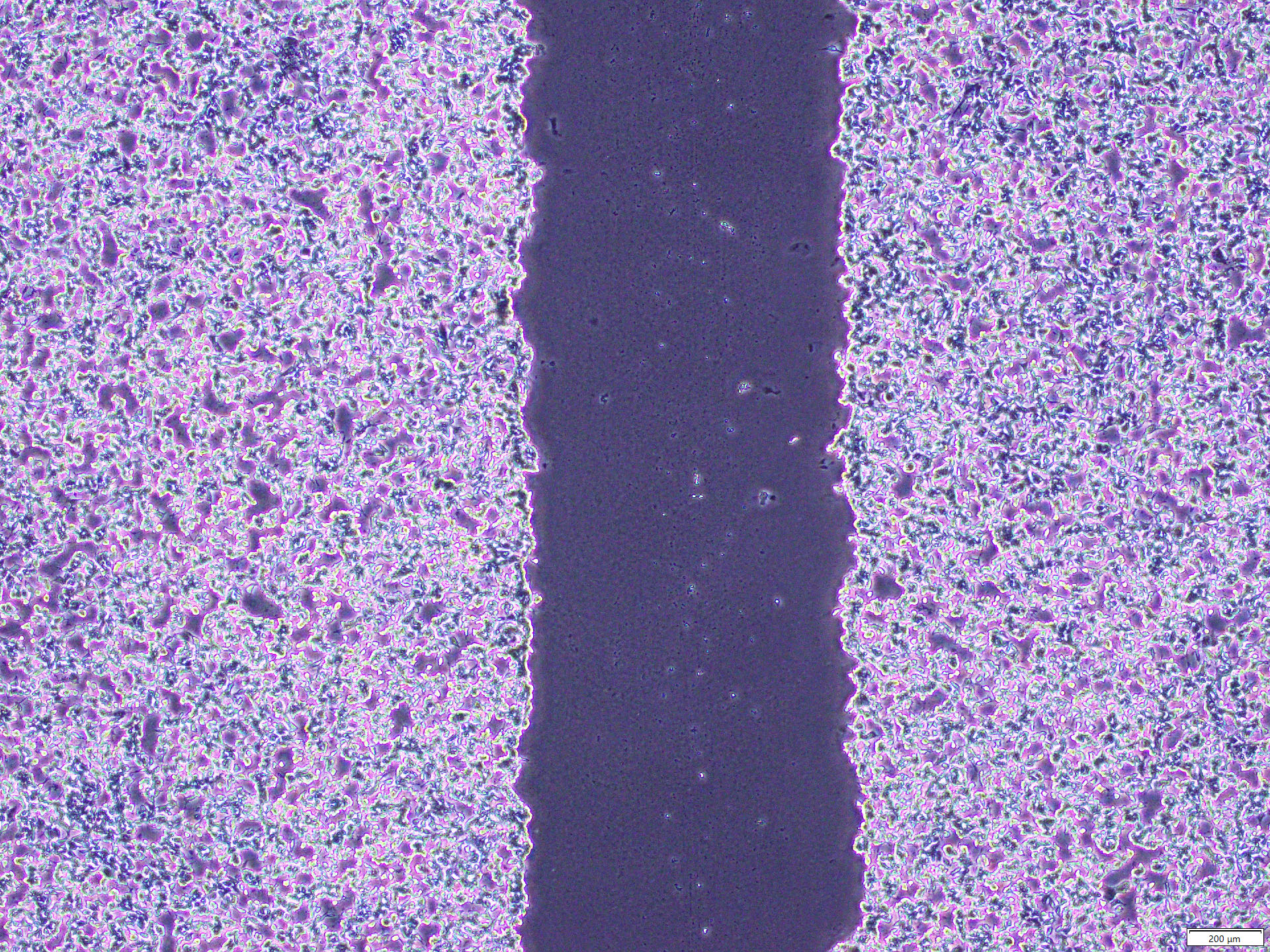

Supplement: S1 File — (ZIP) [file pone.0288180.s011.zip › supplementary Materials/wound healing assay/0h/OE-FDX1-3 U2OS.jpg]

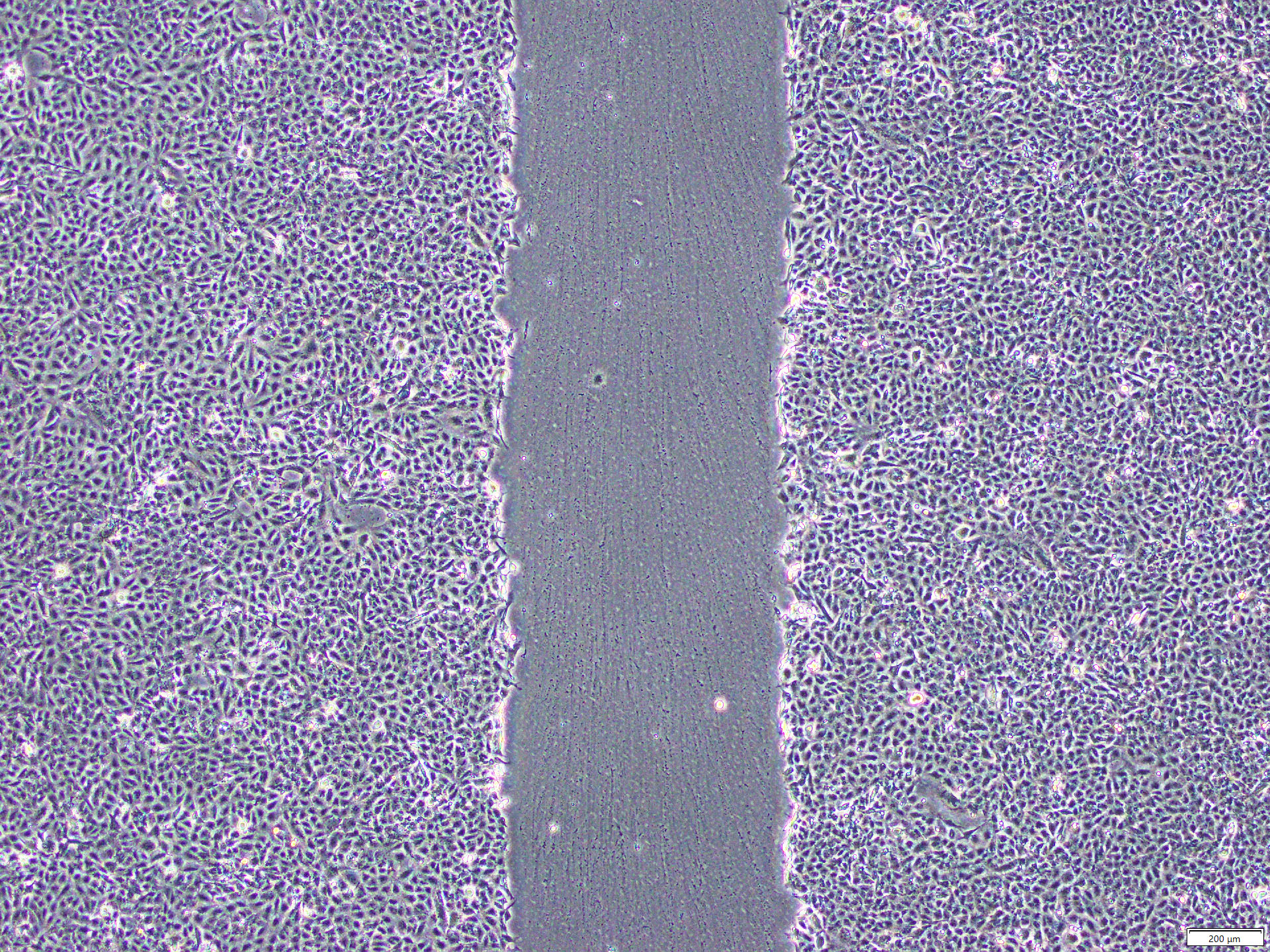

Supplement: S1 File — (ZIP) [file pone.0288180.s011.zip › supplementary Materials/wound healing assay/0h/U2OS-1.jpg]

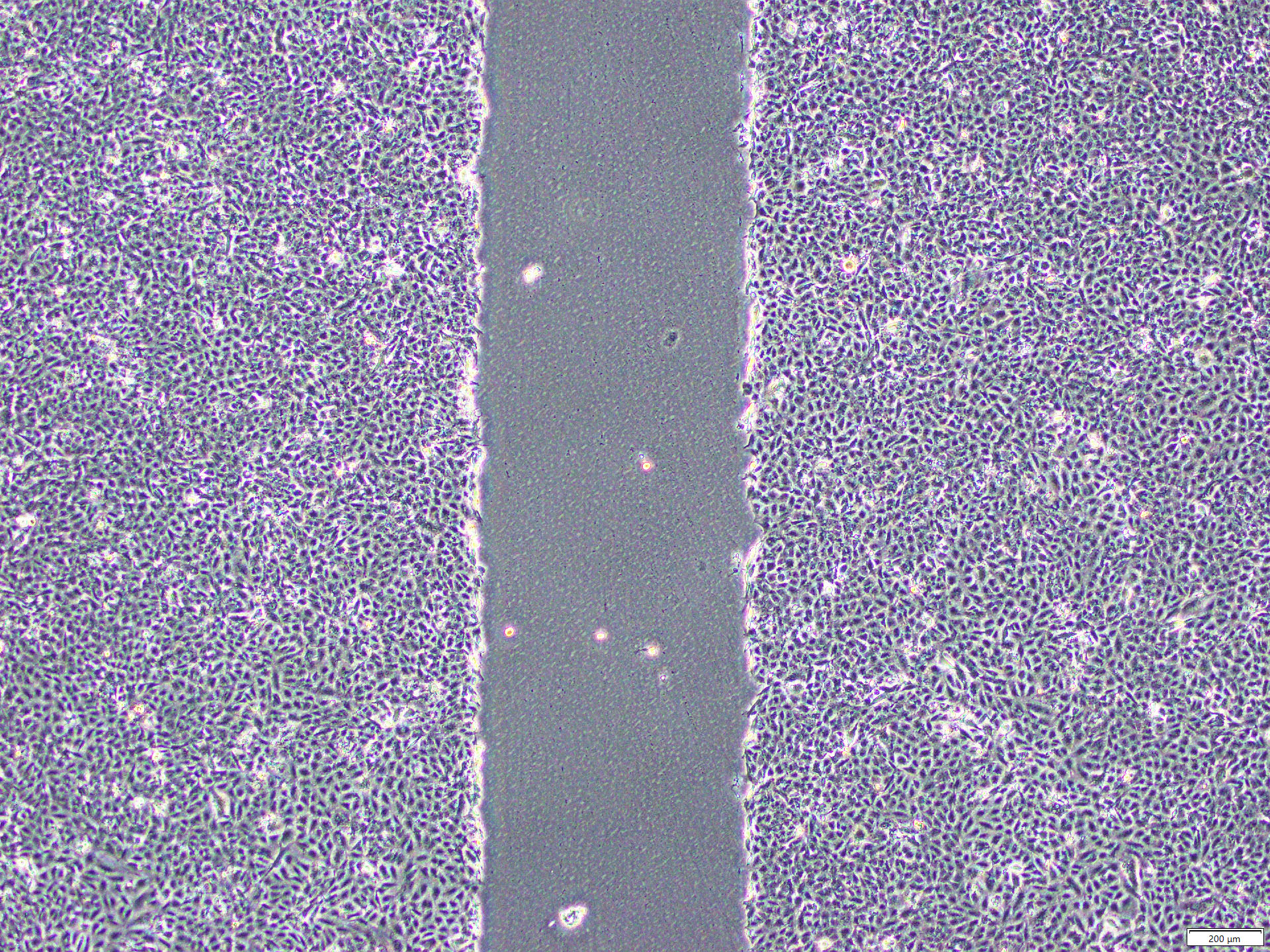

Supplement: S1 File — (ZIP) [file pone.0288180.s011.zip › supplementary Materials/wound healing assay/0h/U2OS-2.jpg]

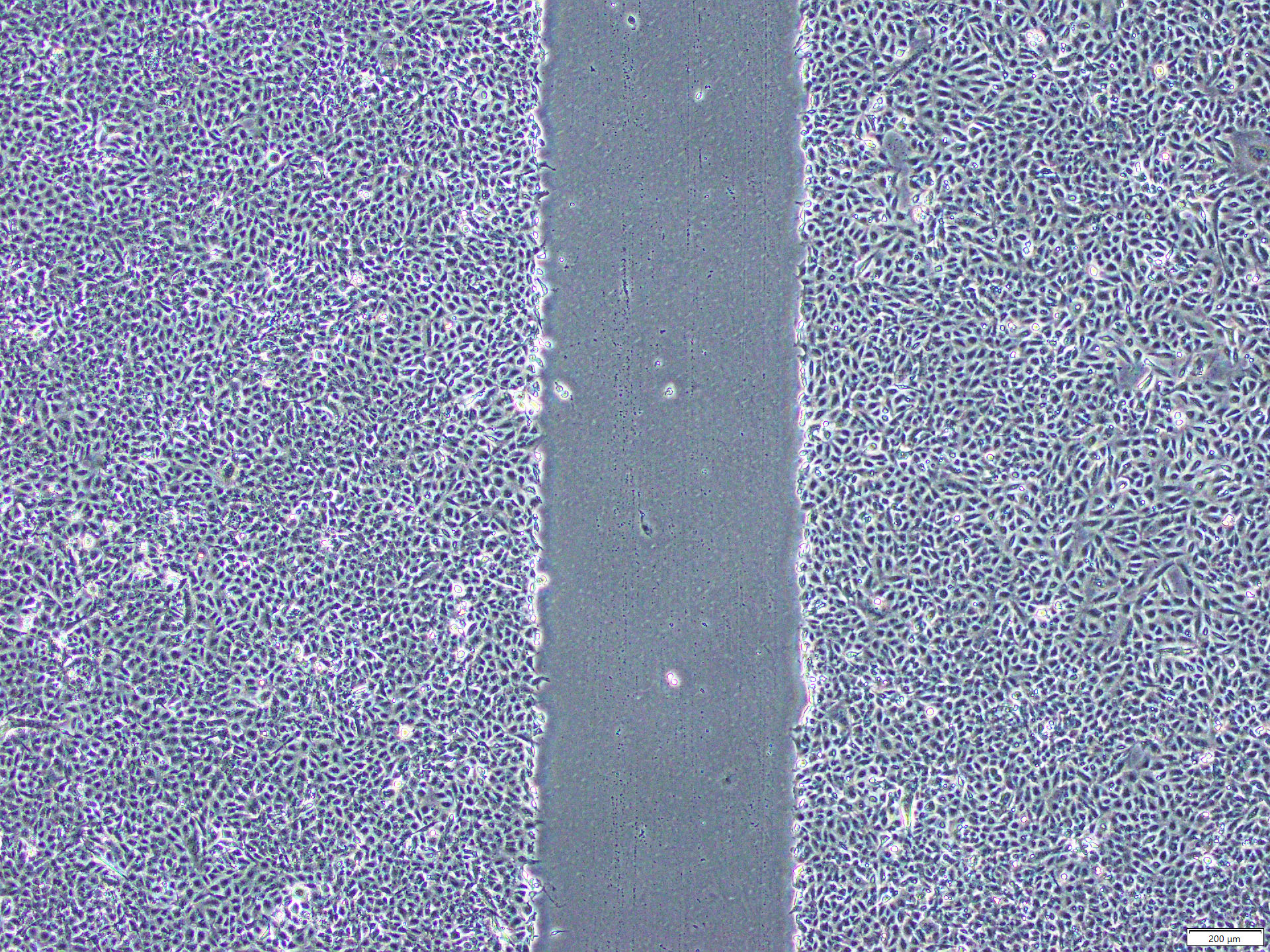

Supplement: S1 File — (ZIP) [file pone.0288180.s011.zip › supplementary Materials/wound healing assay/0h/U2OS-3.jpg]

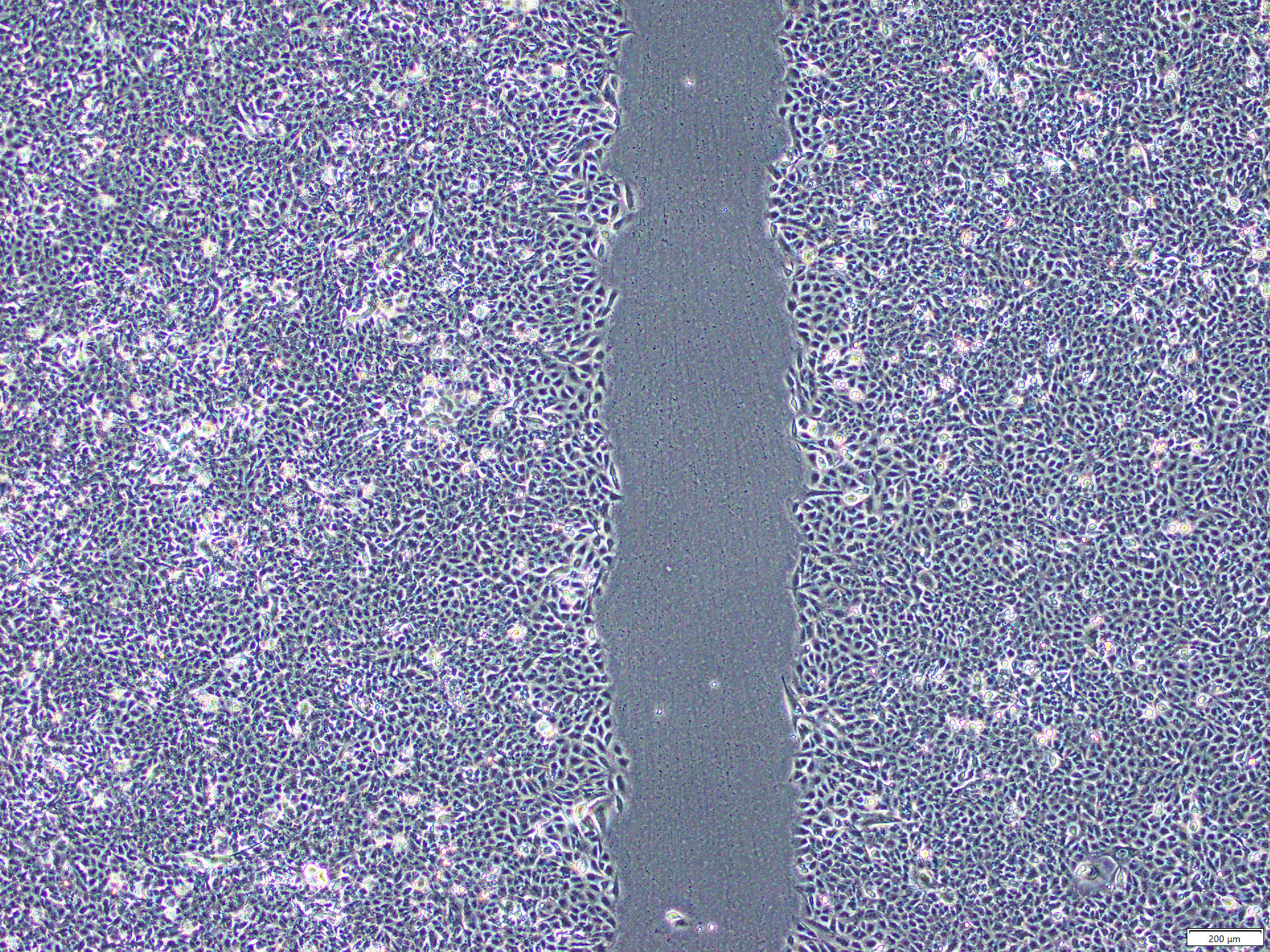

Supplement: S1 File — (ZIP) [file pone.0288180.s011.zip › supplementary Materials/wound healing assay/24h/U2OS-NC-1.jpg]

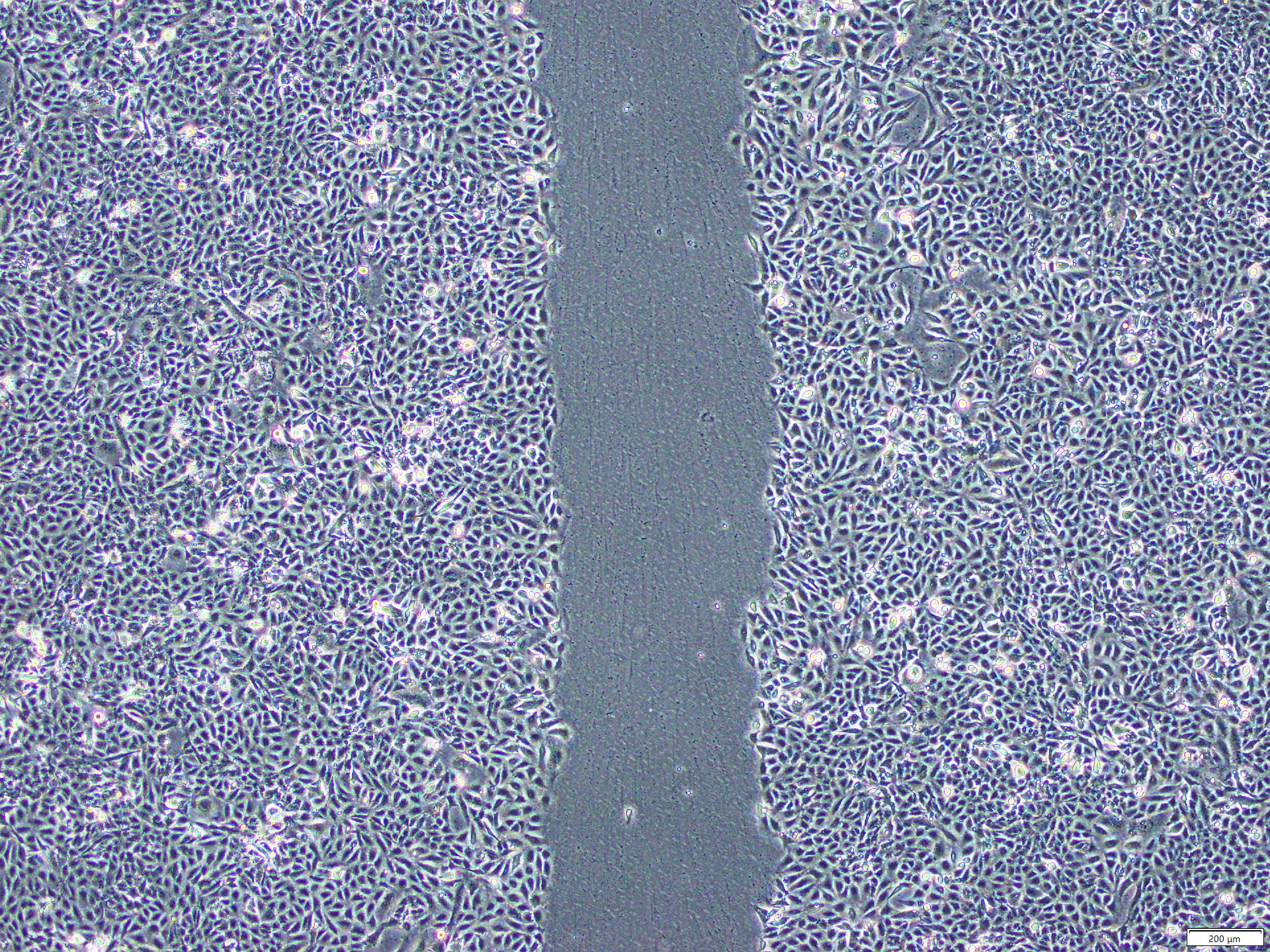

Supplement: S1 File — (ZIP) [file pone.0288180.s011.zip › supplementary Materials/wound healing assay/24h/U2OS-NC-2.jpg]

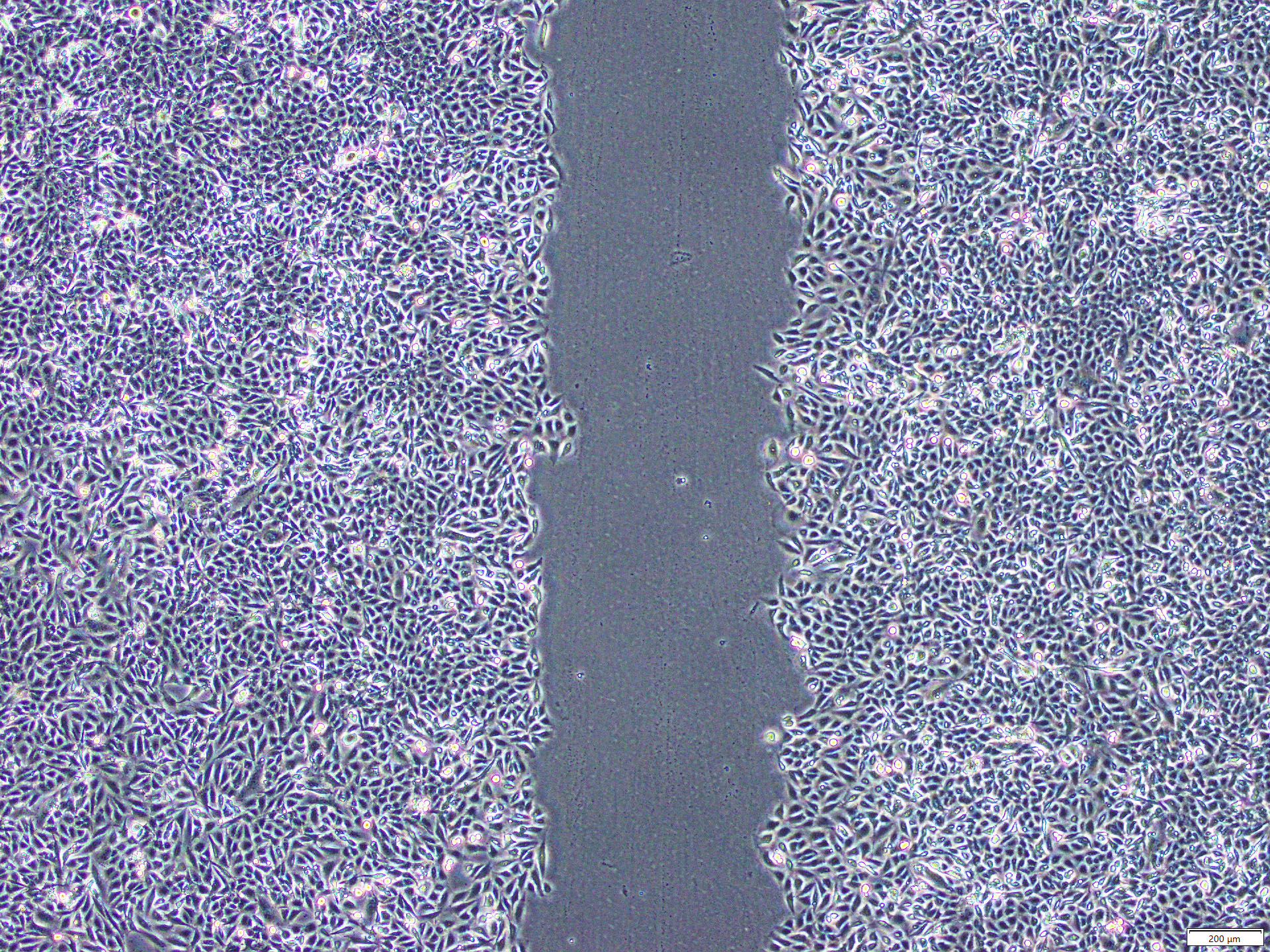

Supplement: S1 File — (ZIP) [file pone.0288180.s011.zip › supplementary Materials/wound healing assay/24h/U2OS-NC-3.jpg]

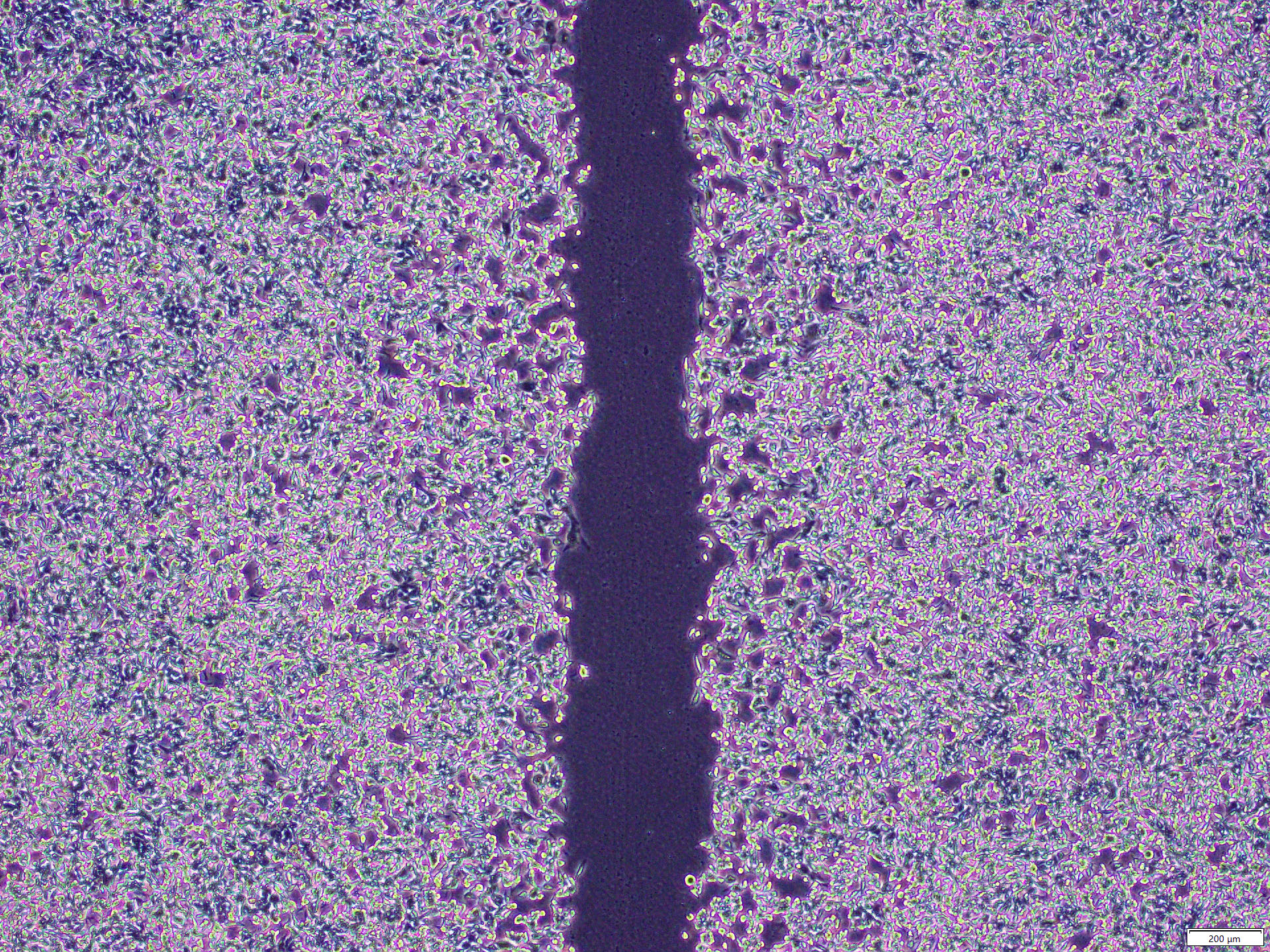

Supplement: S1 File — (ZIP) [file pone.0288180.s011.zip › supplementary Materials/wound healing assay/24h/U2OS-OE-FDX1-1.jpg]

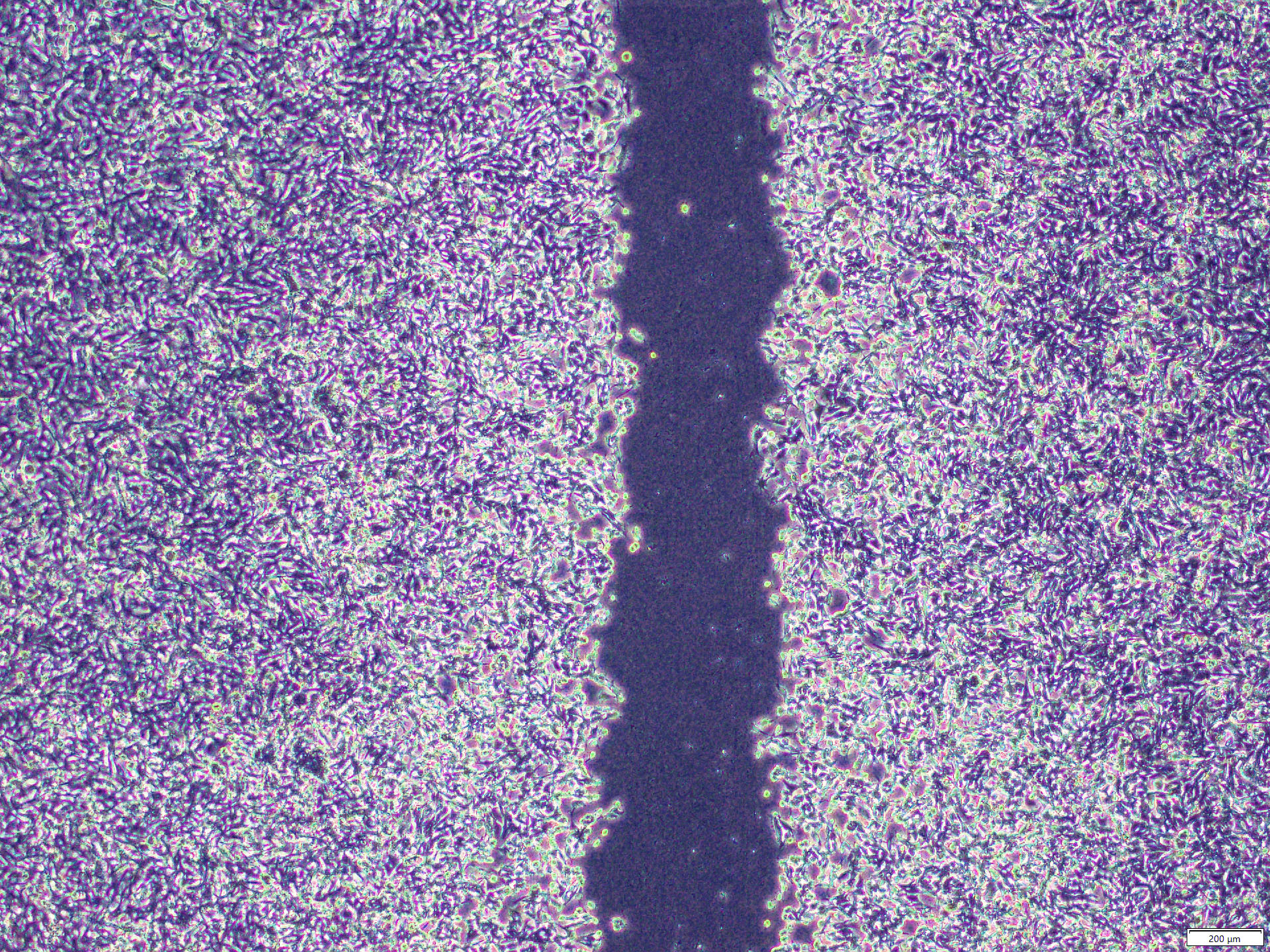

Supplement: S1 File — (ZIP) [file pone.0288180.s011.zip › supplementary Materials/wound healing assay/24h/U2OS-OE-FDX1-2.jpg]

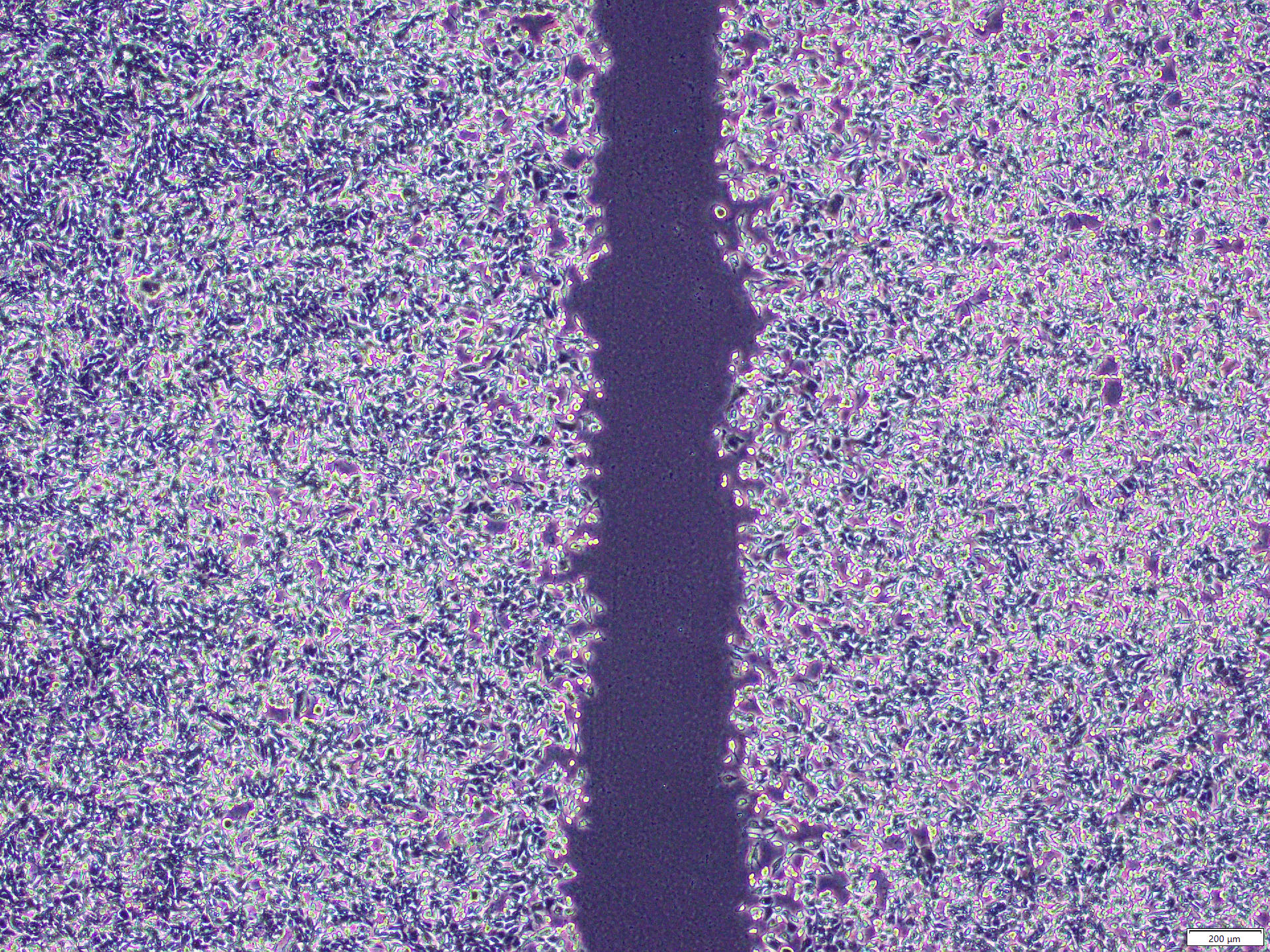

Supplement: S1 File — (ZIP) [file pone.0288180.s011.zip › supplementary Materials/wound healing assay/24h/U2OS-OE-FDX1-3.jpg]
