# Supplementary material for: Construction and validation of a novel cuproptosis-mitochondrion prognostic model related with tumor immunity in osteosarcoma
Source: PLoS One. 2023 Jul 5;18(7):e0288180. doi: 10.1371/journal.pone.0288180 (PMC10321638; doi:10.1371/journal.pone.0288180)
Supplement: S1 Table — (DOCX) [file pone.0288180.s010.docx]

**S1 Table: 33 cuproptosis-mitochondrion genes**

FDX1

LIAS

LIPT1

DLD

DLAT

PDHA1

PDHB

MTF1

GLS

CDKN2A

COX11

MFN2

TOMM20

NDUFB9

ATP6V1E1

NFE2L2

NLRP3

ATP7B

ATP7A

SLC31A1

LIPT2

DBT

GCSH

DLST

SURF1

NDUFB2

NDUFB6

NDUFA8

NDUFA1

NDUFC1

TIMMDC1

NDUFC2

NDUFV2
